# Unique binding pattern for a lineage of human antibodies with broad reactivity against influenza A virus

**DOI:** 10.1038/s41467-022-29950-w

**Published:** 2022-05-02

**Authors:** Xiaoyu Sun, Caixuan Liu, Xiao Lu, Zhiyang Ling, Chunyan Yi, Zhen Zhang, Zi Li, Mingliang Jin, Wenshuai Wang, Shubing Tang, Fangfang Wang, Fang Wang, Sonam Wangmo, Shuangfeng Chen, Li Li, Liyan Ma, Yaguang Zhang, Zhuo Yang, Xiaoping Dong, Zhikang Qian, Jianping Ding, Dayan Wang, Yao Cong, Bing Sun

**Affiliations:** 1 State Key Laboratory of Cell Biology, Shanghai Institute of Biochemistry and Cell Biology, Center for Excellence in Molecular Cell Science, Chinese Academy of Sciences; University of Chinese Academy of Sciences, Shanghai, 200031 China; 2 State Key Laboratory of Molecular Biology, National Center for Protein Science Shanghai, Shanghai Institute of Biochemistry and Cell Biology, Center for Excellence in Molecular Cell Science, Chinese Academy of Sciences, Shanghai, 200031 China; 3 Chinese National Influenza Center, National Institute for Viral Disease Control and Prevention, Chinese Center for Disease Control and Prevention, Beijing, 102206 China; 4 Shanghai Public Health Clinical Center, Fudan University, Shanghai, 201058 China; 5 The National Facility for Protein Science in Shanghai (NFPS), Shanghai, 201210 China; 6 School of Life Science and Technology, ShanghaiTech University, Shanghai, 201210 China; 7 State Key Laboratory for Infectious Disease Prevention and Control, National Institute for Viral Disease Control and Prevention, Chinese Center for Disease Control and Prevention, Beijing, 102206 China; 8 Unit of Herpesvirus and Molecular Virology, Key Laboratory of Molecular Virology & Immunology, Institut Pasteur of Shanghai, Chinese Academy of Sciences, University of the Chinese Academy of Sciences, Shanghai, 200031 China

**Keywords:** Influenza virus, Cryoelectron microscopy

## Abstract

Most structurally characterized broadly neutralizing antibodies (bnAbs) against influenza A viruses (IAVs) target the conserved conformational epitopes of hemagglutinin (HA). Here, we report a lineage of naturally occurring human antibodies sharing the same germline gene, V_H_3-48/V_K_1-12. These antibodies broadly neutralize the major circulating strains of IAV in vitro and in vivo mainly by binding a contiguous epitope of H3N2 HA, but a conformational epitope of H1N1 HA, respectively. Our structural and functional studies of antibody 28-12 revealed that the continuous amino acids in helix A, particularly N49_HA2_ of H3 HA, are critical to determine the binding feature with 28-12. In contrast, the conformational epitope feature is dependent on the discontinuous segments involving helix A, the fusion peptide, and several HA1 residues within H1N1 HA. We report that this antibody was initially selected by H3 (group 2) viruses and evolved via somatic hypermutation to enhance the reactivity to H3 and acquire cross-neutralization to H1 (group 1) virus. These findings enrich our understanding of different antigenic determinants of heterosubtypic influenza viruses for the recognition of bnAbs and provide a reference for the design of influenza vaccines and more effective antiviral drugs.

## Abstract (web summary)

While most broadly neutralizing antibodies (bnAb) against Influenza virus target conserved conformational epitopes of the glycoprotein hemagglutinin (HA), Sun et al. characterize a lineage of bnAbs that neutralize group 1 and 2 strains. Structural characterization shows that antibody 28-12 binds a continuous epitope within H3 (group 2) but requires a conformational epitope for H1 (group 1) binding. Comparison of germline-reverted Ab and intermediate mutants provides evidence for an evolutionary adaptation from group 2 to group 1 strain.

## Introduction

Influenza A virus (IAV) infection remains a serious and persistent threat to global public health^1^. Given the rapid antigen drift and shift of influenza viruses, the current seasonal influenza vaccines are insufficient to meet broad social needs. The emergence of drug-resistant influenza viruses limits the widespread use of anti-influenza drugs^2,3^. Therefore, there is an urgent medical need for a more universal solution to meet the challenge of influenza A viruses. Genetically, 16 IAV subtypes are classified into two distinct groups according to the phylogenetics of hemagglutinin (HA)^2,4,5^, with another two new analogous HAs isolated from bats (named H17 and H18)^6,7^. Usually, the H5 and H7 subtypes are highly pathogenic avian influenza viruses associated with sporadic severe human infection^8^. H1 and H3 subtypes circulate annually in human society and are therefore the components of seasonal influenza vaccines^1,9^. The envelope glycoprotein HA of IAV is the major target of neutralizing antibodies^10^. The globular head domain mediates the receptor binding process, and the HA stem leads to virus-cell membrane fusion induced by the low pH in endosomes^1,11^.

Several human antibodies have been isolated from vaccinated or naturally infected individuals that target conserved epitopes within HA that showed different levels of cross-reactivity towards group 1 and group 2 influenza A viruses. The majority of bnAbs target the HA stem region, thus neutralizing influenza A viruses by inhibiting the low pH-induced conformational rearrangement of HA, hence blocking membrane fusion; while a small fraction of them targets the conserved HA head region and block HA-sialic acid receptor binding^9,12–17^. V_H_1-69, V_H_1-18, V_H_3-30 and V_H_6-1 have shown dominant biased usage of germline genes for the stem-directed bnAbs against IAVs^18^. The characterized bnAbs have provided powerful tools to identify such dominant germline scaffolds, thus speeding up the development of broad-spectrum therapeutic agents^19^. Moreover, understanding how bnAbs overcome highly diversified HA antigenic variations to broadly neutralize divergent IAVs would also contribute to the development of universal influenza vaccines.

In the current study, we report a lineage of naturally occurring human antibodies isolated from a healthy donor who was vaccinated with a trivalent seasonal split vaccine. The representative antibody 28-12 exhibited broad neutralizing activity against both group 1 and group 2 IAV viruses in vitro and protected mice against H3N2 and H1N1 virus challenge in vivo. The character of this lineage of human antibodies is unusual. These antibodies (28-2, 28-4, 28-6, and 28-12) belong to the same lineage carrying V_H_3-48/D_H_2-2/J_H_6 and V_K_1-12/J_K_5 gene segments. It is worth noting that these antibodies showed different epitope determinants between different subtypes that they mainly bind a continuous epitope of H3N2 HA, while recognizing H1N1 HA in a conformation dependent pattern. The reason can be elucidated from our functional studies combined with our cryo-EM structures of 28-12 Fab in complex with HAs of H1N1 and H3N2. The binding activity of 28-12 to H1N1 is much more dependent on helix A, the fusion peptide and several HA1 residues, which are discontinuous in the primary sequence. Nevertheless, the continuous epitope within the helix A of H3N2 HA makes the major contribution to the binding of 28-12. We further revealed that N49_HA2_ is the critical residue to determine the epitope feature of H3N2 HA by 28-12; however, the equivalent residue is substituted by T49_HA2_ in H1N1. We further revealed that 28-12 was likely first primed by group 2 viruses (H3) and further enhanced the reactivity to H3 and acquired cross-neutralization against group 1 viruses (H1). We described the putative key somatic hypermutations of 28-12 to achieve broad and potent neutralization. This study improves our understanding of the significant flexibility of bnAbs in recognizing distinct antigenic determinants among different IAV subtypes. Our finding supports 28-12 as a potential therapeutic reagent for both prophylaxis and treatment of circulating H1N1 and H3N2 IAV infection in humans and provide a reference for the design of broad influenza vaccines and more effective antiviral drugs.

## Results

### Isolation and characterization of V_H_3-48/V_K_1-12 lineage antibodies

**Fig. 1:**
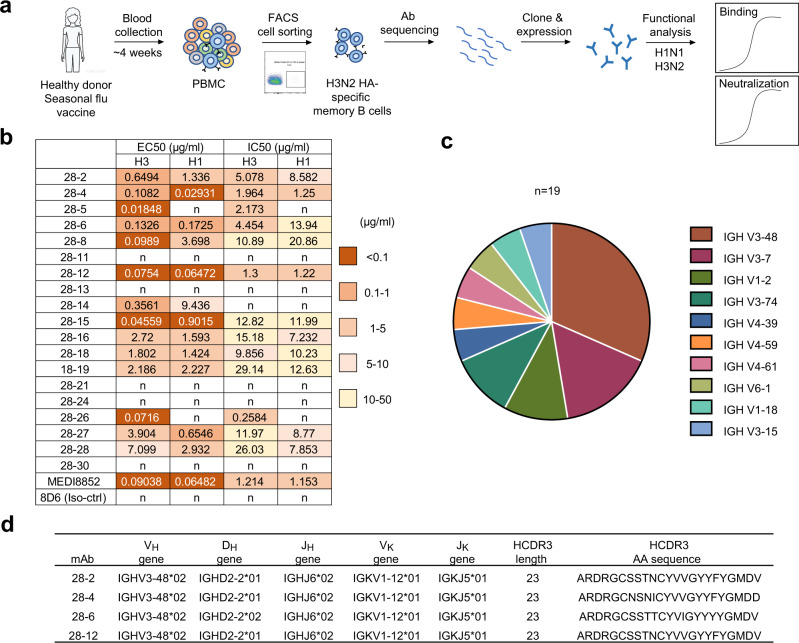
A lineage of antibodies with cross-reactivity to H3N2 and H1N1 subtypes were identified. **a** Schematic of isolation of influenza HA specific antibodies from an individual vaccinated with trivalent inactivated influenza vaccine. The isolated antibodies were tested for dose-dependent binding and neutralizing activities against group 2 HK/68 H3N2 and group 1 SC/09 H1N1 (**b**). 8D6, an unrelated HCV antibody was included as a negative control. The EC50 and IC50 values are shown into heatmap. n, not binding or not neutralizing. **c** Distribution of V gene families in heavy chains of all distinct clones (*n* = 19). **d** Summary of the germline (V-D-J for heavy chain and V-J for light chain) and HCDR3 of V_H_3-48/V_K_1-12 lineage antibodies.

We conducted single-cell PCR experiments to screen mAbs that might protect against heterosubtypic IAV infection from memory B cells of one healthy individual ~4 week after vaccination with seasonal trivalent influenza vaccine in 2016, which contains three components, A/California/7/2009(H1N1) pdm09-like virus, A/Victoria/361/2011(H3N2)-like virus and B/Wisconsin/1/2010-like virus (Fig. 1a). We performed memory B cell screening experiments using group 2 A/HongKong/01/1968 (HK/68) H3N2 HA protein as the bait. As the H1N1 and H3N2 subtypes are the most prevalent circulating strains, subsequent cloning and screening of antibodies for reactivity were conducted with soluble HA proteins and human infecting viruses of both HK/68 H3N2 and SC/09 H1N1(A/Sichuan/01/2009), which led to the identification of a series of antibodies with different levels of cross-reactivity towards H1N1 and H3N2. Among these antibodies, eleven mAbs cross-neutralizes both H3N2 and H1N1. Notably, 28-4, and 28-12 showed better cross-neutralizing activity with IC50 under 2 μg/ml as compared with other isolated antibodies in this study, which is comparable with a previously reported antibody MEDI8852 (Fig. 1b).

We performed gene sequence analysis of the isolated antibodies by using IMGT and IgBLAST. A large portion of antibodies utilized the V_H_3-48 germline genes (Fig. 1c), of which, 28-2, 28-4, 28-6 and 28-12, belong to the same lineage carrying V_H_3-48/D_H_2-2/J_H_6 and V_K_1-12/J_K_5 gene segments with cross-group neutralizing activity (Fig. 1b and Supplementary Fig. 1). We named 28-2, 28-4, 28-6 and 28-12 antibodies as V_H_3-48/V_K_1-12 lineage antibodies.

### Broad binding and neutralization profile of V_H_3-48/V_K_1-12 lineage antibodies

**Fig. 2:**
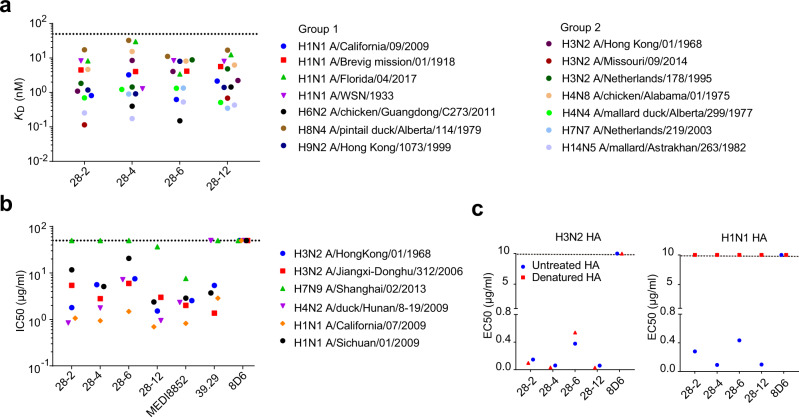
V_H_3-48/V_K_1-12 lineage antibodies broadly bind and neutralize multiple influenza subtypes. **a** Binding affinity (*K*_D_) of each mAb to HA proteins from a panel of group 1 and group 2 influenza A isolates as measured by BLI. Dashed line indicates 50 nM. **b** In vitro neutralizing activity of each mAb against multiple strains of H1, H3, H4, and H7 subtype IAVs, shown as IC50 values. MEDI8852 and 39.29 were included as positive controls while 8D6 was the negative control. Dashed line indicates 50 μg/ml. The hosts of non-human infecting IAV strains have been noted in the full name of IAV strains, while the other IAV strains without host annotation are human infecting. **c** ELISA binding EC50 values of each mAb to denatured or untreated HAs of H3N2 and H1N1. Representative data are shown from two independent experiments.

To verify the reactive breadth of the V_H_3-48/V_K_1-12 lineage antibodies, BLI-based *K*_D_ values were measured within each mAb to purified soluble HA proteins from divergent subtypes of group 1 and group 2 IAVs. Each V_H_3-48/V_K_1-12 lineage mAb displayed comparable binding constant (*K*_D_ value) to each HA protein from different subtypes that belong to group 2 (the human infecting H3, H7 strains and the avian H4, H14 strains) and group 1 (the human infecting H1, H9 strains and the avian H6, H8 strains) viruses with *K*_D_ values ranging from 0.001 to 32.5 nM (Fig. 2a).

To extend the evaluation, we directly compared the in vitro neutralization activity and breadth of V_H_3-48/V_K_1-12 lineage antibodies with those of MEDI8852 and 39.29 using a diverse panel of influenza strains from group 1 and group 2 viruses. All V_H_3-48/V_K_1-12 lineage antibodies neutralized the tested H3N2 (A/HongKong/01/1968 and A/Jiangxi-Donghu/312/2006) and H1N1 (A/Sichuan/01/2009 and A/California /07/2009) viruses. Most of the antibodies tested failed to neutralize the A/Shanghai/02/2013 H7N9 virus, except for MEDI8852 and 28-12, with IC50 values of 7.615 and 36.64 μg/ml, respectively. Antibody 39.29 failed to neutralize the A/duck/Hunan/8-19/2009 H4N2 virus at the highest concentration tested (50 μg/ml). 28-12 exhibited neutralizing potency against group1 H1 strains with an average IC50 values of 1.53 μg/ml, compared to values of 6.38, 3.02 and 11.02 μg/ml for 28-2, 28-4 and 28-6, respectively. They also neutralize group 2 H3 strains with average IC50 values of 3.61, 4.19, 6.72 and 2.256 μg/ml for 28-2, 28-4, 28-6 and 28-12, respectively. MEDI8852 and 39.29 were included as positive controls with comparable IC50s to H1 and H3 strains (Fig. 2b).

To further define whether the binding of the V_H_3-48/V_K_1-12 lineage antibodies to HA proteins was conformation dependent, we denatured the H3 and H1 HA proteins and performed ELISA. We found the V_H_3-48/V_K_1-12 lineage antibodies showed comparable reactivity with both untreated and denatured H3 HA. In contrast, they lost the ability to bind the denatured H1 HA (Fig. 2c). Further study showed that 28-12 mainly recognizes a continuous epitope in H3 clade subtypes, but a conformational epitope in H7 clade and some group 1 subtypes, indicating a rare and unique recognition pattern (Supplementary Fig. 2a–f).

As reported, some HA stem bnAbs are typically prone to polyreactive^20,21^. From the ELISA and immunofluorescence-based assays, we found the V_H_3-48/V_K_1-12 lineage antibodies are HA specific and have little or no binding affinity to other self-antigens and proteins in HEp-2 cells (Supplementary Fig. 3).

### Prophylactic and therapeutic efficacy of 28-12 in vivo

**Fig. 3:**
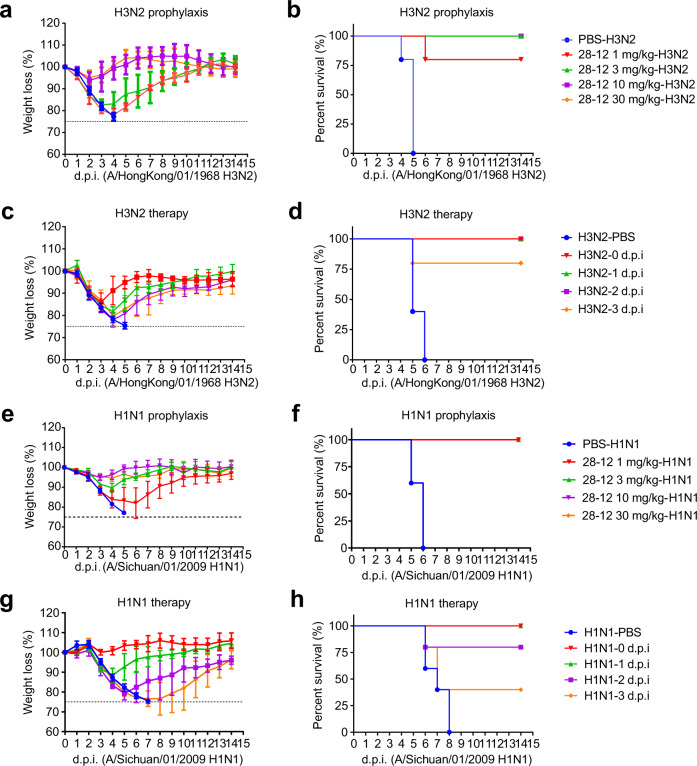
Prophylactic and therapeutic efficacy of 28-12 in mice. **a**, **b** Prophylactic efficacy of 28-12 against a lethal challenge with 68/HK H3. Mice were treated with 30, 10, 3 or 1 mg/kg 28-12 or PBS 24 h before intranasal inoculation with the influenza virus. The weight loss and survival data were collected daily for 14 days after inoculation (day 0). *n* = 5. **c**, **d** Therapeutic efficacy of 28-12 against a lethal challenge with 68/HK H3. Mice were treated with PBS buffer (at day 0) or 25 mg/kg 28-12 immediately after or 1, 2, or 3 days after intranasal inoculation with influenza virus. The weight loss and survival data were collected daily for 14 days after inoculation with viruses (day 0). *n* = 5. **e**, **f** Prophylactic efficacy of 28-12 against a lethal challenge with SC/09 H1. *n* = 5. **g**, **h** Therapeutic efficacy of 28-12 (25 mg/kg) against a lethal challenge with SC/09 H1. *n* = 5. Error bars represent the mean ± S.D (**a**, **c**, **e**, **g**). All data show a representative experiment from two independent experiments.

We further carried out in vivo protection studies of 28-12 in mice against lethal influenza challenges of two different strains, group 1 SC/09 H1 and group 2 HK/68 H3. In the prophylaxis assay, the mice were first administered different doses of 28-12 (30, 10, 3, or 1 mg/kg) and then challenged with a lethal dose of H1N1 and H3N2 viruses 24 h later. The administration of a lower dose of 28-12 (1 or 3 mg/kg) fully protected the mice from H1N1 (Fig. 3e, f) or H3N2 (Fig. 3a, b) infection. To evaluate the therapeutic efficacy of 28-12, we administered 28-12 (25 mg/kg) at different time points following infection with SC/09 H1 or HK/68 H3 viruses. The data showed that all the mice survived when treated with 28-12 at day 2 post infection with HK/68 H3, and 80% survival was achieved when inoculation was delayed for 3 days, with slightly reduced body weight, which recovered starting on day 3 or day 4 (Fig. 3c, d). In the challenge model of SC/09 H1, 80% or 100% of the mice survived when treated with 28-12 1 or 2 days’ post infection, while only 40% survival was observed when antibody treatment was delayed for 3 days (Fig. 3g, h). Overall, these results indicate that 28-12 could broadly protect mice against both H1N1 and H3N2 viruses in vivo.

### Cryo-EM structures of 28-12 Fab-HA complexes

**Fig. 4:**
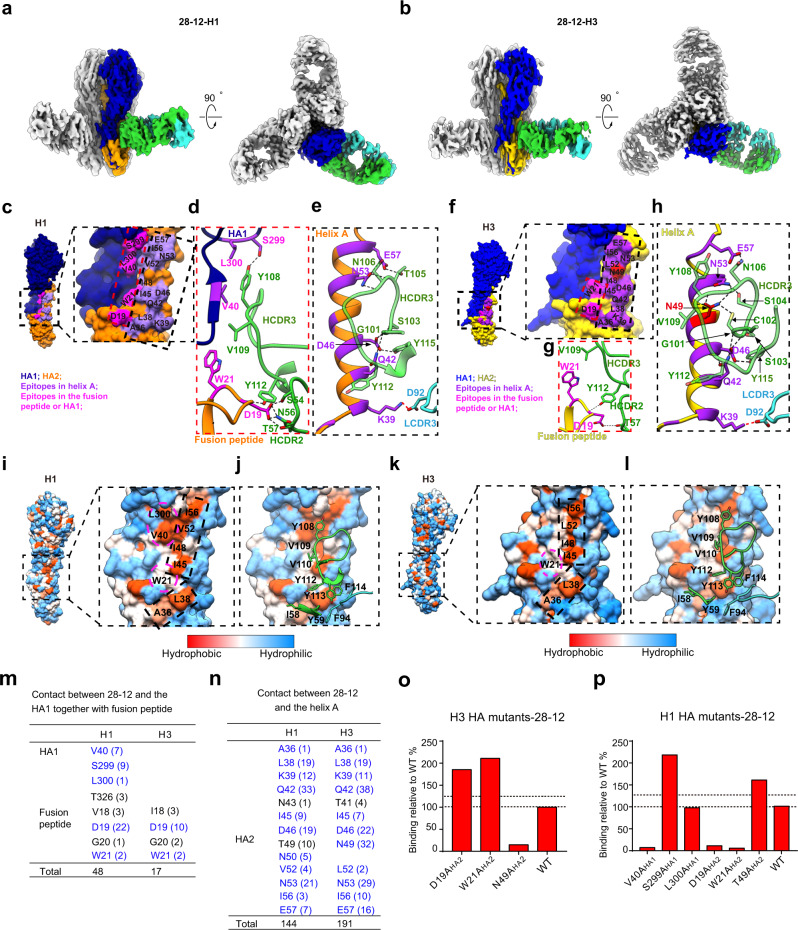
Cryo-EM structures of 28-12-H3 and 28-12-H1 and distinct recognition patterns of 28-12 Fab to H3 and H1 HAs. **a**, **b** Cryo-EM maps of the 28-12-H1 (**a**) and 28-12-H3 (**b**) complexes. One HA protomer and the cognate 28-12 Fab fragment are highlighted in color, and the other two copies in the trimer are colored gray. H1 HA1/HA2 and H3 HA1/HA2 are colored in navy blue, orange, blue, and gold, respectively. The heavy and light chains of 28-12 Fab are colored in lime green and turquoise, respectively. This color scheme was followed throughout. **c**, **f** Epitopes of the 28-12 Fab on H1 (**c**) and H3 (**f**), respectively. The epitopes in helix A (indicated by black dashed box) and fusion peptide/HA1 (indicated by red dashed box) are colored in purple and magenta, respectively. H3 residue N49_HA2_ is specifically showed in red. **d**, **g** Hydrogen bonds between 28-12 Fab and the fusion peptide/HA1 of H1 (**d**) or H3 (**g**). **e**, **h** Hydrogen bonds formed between helix A of H1 (**e**) or H3 (**h**) and 28-12 Fab. The salt bridge and hydrogen bond are shown as red and black dashed lines, respectively. **i**, **k** Hydrophobic epitopes in the H1 (**i**) and H3 (**k**) HAs. The hydrophobic epitopes are classified into two parts, helix A in the dashed black box and the fusion peptide and HA1 residues in the dashed magenta circle. **j**, **l** The Fab residues involved in hydrophobic contacts with H1 (**j**) and H3 (**l**) are shown with side chains. **m** The number of atom-to-atom contacts between 28-12 Fab and the fusion peptide/HA1 residues. **n** The number of atom-to-atom contacts between 28-12 Fab and the helix A. The epitopes involved in the hydrophobic interaction, hydrogen bond, and salt bridge are colored in blue. **o**, **p** 28-12 binding to H1 and H3 HA mutants with alanine substitutions. Each value is calculated as the binding ratio relative to that of the WT HA (%). The two black dotted lines represent 100 and 125% relative binding to the WT data, respectively. The mean values of duplicates are shown from two independent experiments (**o**, **p**).

Our biochemical and functional data suggested that V_H_3-48/V_K_1-12 lineage antibodies neutralize H3N2 mainly by targeting a continuous epitope of HA, while reacting to a conformational epitope to H1N1 (Fig. 2c). To provide the structural basis of the unique recognition pattern, we determined the cryo-EM structures of the 28-12 Fab in complex with the trimeric HAs from HK/68 H3 and WA/11 H1 at 3.7-Å- and 3.5-Å-resolutions, respectively, with imposed C3 symmetry (Fig. 4a, b and Supplementary Figs. 4–5). To overcome the preferred orientation problem (preferred top-view orientation) associated with the 28-12-H3 complex, also seen in the H3N2 Hong Kong HA trimer (HK/68 H3)^22^, we adopted the stage-tilt strategy for data collection^22^. We then built an atomic model for the 28-12-H3 and 28-12-H1 complexes, respectively, and the model fits in the corresponding map well (Supplementary Figs. 4e, f and 5d, e).

### The structural and functional basis for the unique recognition pattern of 28-12

Our two cryo-EM structures showed that the 28-12 Fab specifically recognizes the conserved HA stem region for both H3 and H1 (Fig. 4a, b). Consistently, 28-12 inhibited cell–cell fusion by blocking low-pH induced HA conformational change (Supplementary Fig. 6). We divided the amino acids that constitute the epitope of 28-12 Fab on H3 or H1 into two parts: amino acids within helix A of HA2, and amino acids within the fusion peptide and HA1 (Fig. 4c, f).

Interestingly, our structural comparison revealed that the binding pattern of 28-12 to H1 and H3 HAs is distinct to some extent. Overall, 28-12 appears to form more contacts with the fusion peptide and the HA1 residues adjacent to helix A in H1 than with those in H3 (Fig. 4c, f, m). For H1, the D19_HA2_ within the fusion peptide forms hydrogen bonds with S54_HCDR2_, N56_HCDR2,_ T57_HCDR2_, and Y112_HCDR3_; S299_HA1_ forms hydrogen bond with Y108_HCDR3_ (Fig. 4d and Supplementary Table 3). Moreover, W21_HA2_ on the fusion peptide forms hydrophobic interactions with V109_HCDR3_. Another two amino acids from HA1, including V40_HA1_ (which is a hydrophilic amino acid N40 in H3) and L300_HA1_ also form hydrophobic interactions with V109 and Y108 both from HCDR3 (Fig. 4d, i, j and Supplementary Table 2).

In contrast, H3 contributes only two epitope residues outside helix A to interact with 28-12. Specifically, H3 D19_HA2_ on the fusion peptide forms hydrogen bonds with T57_HCDR2_ and Y112_HCDR3_ (Fig. 4g and Supplementary Table 3), and H3 W21_HA2_ on the fusion peptide forms hydrophobic interactions with V109_HCDR3_ (Fig. 4g, k, l and Supplementary Table 2). Besides, we also calculated the atom-to-atom contacts between 28-12 and the fusion peptide/HA1 of H1 and H3, respectively, under a distance cut-off value of 4.5 Å (Fig. 4m and Supplementary Tables 4 and 5). It appears that 28-12 forms more contacts with H1 (48 contacts) than that with H3 (17 contacts). On the basis of our structural analysis, we performed further site-directed single mutation of the epitope residues in the fusion peptide and HA1 and determined the binding activity between HA and 28-12. The mutations D19A_HA2_, W21A_HA2_, and V40A_HA1_ in H1 significantly decreased H1 HA reactivity to 28-12 (Fig. 4p), while the mutations D19A_HA2_ and W21A_HA2_ in H3 did not affect H3 HA binding activity to 28-12 (Fig. 4o). Collectively, these results indicate that the epitope in the fusion peptide and HA1 is indispensable for 28-12 to react with H1.

Furthermore, helix A is another critical element for 28-12 to recognize both H3 and H1. Our structural analysis suggested that there are epitope amino acids shared by both H3 and H1, including Q42_HA2_, D46_HA2_, N53_HA2_, and E57_HA2_ forming hydrogen bonds with HCDR3, and K39_HA2_ forming salt bridge with D92_LCDR3_ (Fig. 4c–h and Supplementary Table 3). In addition, we found that A36_HA2_, L38_HA2_, I45_HA2_, I48_HA2_, I56_HA2_, and W21_HA2_ form hydrophobic interactions with HCDR2 or HCDR3 (Fig. 4i–l and Supplementary Table 2). Notably, H3 has a unique epitope residue, N49_HA2_, which forms strong hydrogen bonds with C102, N106, Y108, and V109 on HCDR3 (Fig. 4h and Supplementary Table 3). Overall, the atom-to-atom contact numbers between 28-12 and H1 or H3 helix A are 144 and 191, respectively (Fig. 4n and Supplementary Tables 4 and 5). It is worth noting that the H3 residue N49_HA2_ contributes much more contact numbers (32) with 28-12 than the substitute residues T49_HA2_ (10) of H1 (Fig. 4n and Supplementary Tables 4 and 5). The site-directed mutational binding assay also confirmed that N49_HA2_ is critical for H3 HA binding to 28-12, while T49A_HA2_ did not affect the binding between H1 HA and 28-12 (Fig. 4o, p). Collectively, these results indicate that the epitopes within helix A are critical for 28-12 to react with both H1 and H3, and form more interactions with H3.

Interestingly, antibody 28-12 harbors a long HCDR3 with 23 amino acids compared with many of the reported bnAbs^9,12,13,23^ (Supplementary Fig. 1 and 7), in line with our structural observation that HCDR3 heavily contributes to the interactions with HA proteins, which form multiple hydrogen bonds or hydrophobic interactions with antigens (Fig. 4c–n). We also compared the HCDR3 of 28-12 with 3I14 which also has a long HCDR3 (23aa) in length. For one single protomer (on the primary protomer), 3I14 HCDR3 mainly makes hydrophobic and hydrogen bonds with helix A and the fusion peptide, which is similar with 28-12 HCDR3 in recognizing H3 HA. Additionally, 28-12 HCDR3 also contacts with several HA1 residues of H1, but not H3, for the primary protomer. It seems that the HCDR3 of 28-12 is more significant than that of 3I14 to recognize HA as it contributes more amino acids to interact with H3 HA (12) and H1 HA (11), respectively, while 3I14 devotes 7 HCDR3 amino acids to interact with H3 or H6 (Supplementary Fig. 7).

**Fig. 5:**
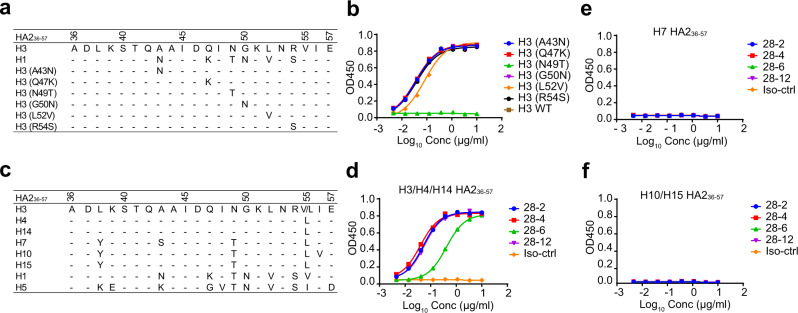
N49_HA2_ is critical for the recognition of H3 epitope features by V_H_3-48/V_K_1-12 lineage antibodies. **a** Sequence alignment of the conserved epitope 36-57_HA2_ of H1 and H3**. b** Dose-dependent binding of 28-12 to divergent mutants of H3 peptide 36-57_HA2_ compared with the wild-type peptide. **c** Sequence alignment of 36-57_HA2_ within multiple subtypes. H3/H4/H14, but not other subtypes have residue N in position 49_HA2_. **d**–**f** Dose-dependent reactivity of the V_H_3-48/VK1-12 lineage antibodies to peptide 36-57_HA2_ of multiple subtypes. 8D6 was included as the isotype control. Data are presented as mean values of duplicates (**b**, **d**, **e**, **f**). Representative data are shown from two independent experiments (**b**, **d**, **e**, **f**).

We also found that the cleavage of HA0 (the activated HA) doesn’t affect the binding activity of 28-12 to this epitope on H3N2 HA, but obviously impairs the reactivity to H1N1 HA, as compared with the binding to HA0 protein (Supplementary Fig. 8). These data further indicate the distinct intrinsic properties of HA proteins from different HA subtypes within 28-12 epitope.

### N49_HA2_ is critical for 28-12 binding to the continuous epitope within H3 helix A

To further confirm the critical residues composing the epitope for 28-12 binding to H3, we conducted sequence alignment of HA2 residues 36-57 between H1 and H3 and identified 6 different residues (Fig. 5a). We synthesized 6 peptides with single substitution mutations from H3 to H1 and found that the binding ability decreased significantly when there was a mutation from N to T at position 49 of HA2 compared with the wild-type peptide. Nevertheless, other single mutations did not affect binding (Fig. 5b). This result is also consistent with our structural data that H3 N49_HA2_, but not H1 T49_HA2_, strongly interacts with HCDR3 residues Y108 and V109 of 28-12, with hydrogen bonds and more atom-to-atom contacts (Fig. 4e, h, n and Supplementary Table 3). Sequence alignment of 36-57_HA2_ within divergent subtypes showed that all H3 clade IAVs (H3/H4/H14) share a residue N at position 49_HA2_ but a residue T in other subtypes (Fig. 5c). Similarly, the V_H_3-48/V_K_1-12 lineage antibodies reacted well to the peptides 36-57_HA2_ of H3/H4/H14 subtypes, but not other subtypes (Fig. 5d–f). In brief, these data demonstrated that the residue N49_HA2_ of helix A is critical for 28-12 binding to the continuous antigenic epitope of H3, and further indicate different epitope features of HA proteins between H1 and H3 subtypes for antibody binding.

**Fig. 6:**
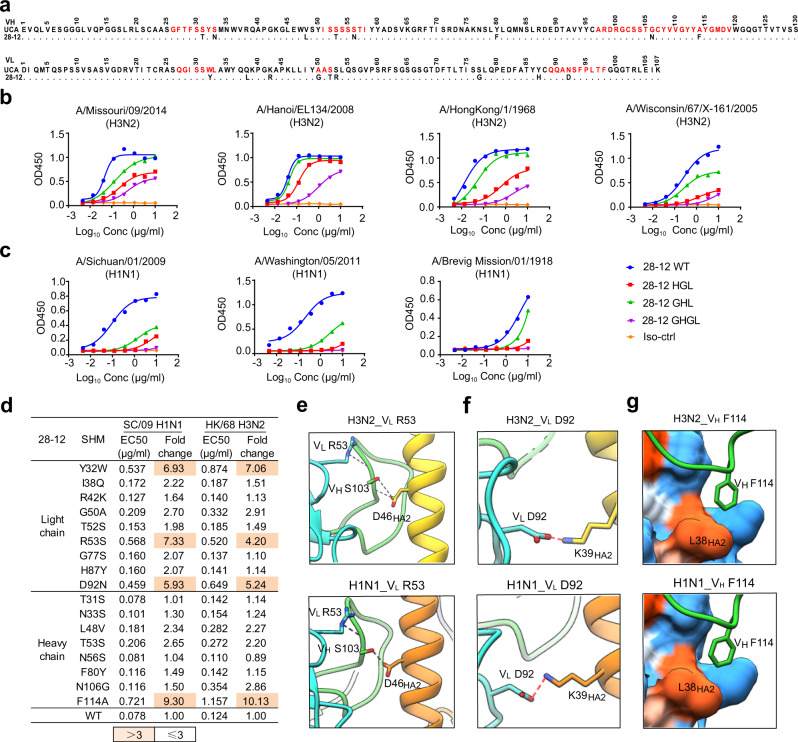
Somatic hypermutations of 28-12. **a** Sequence alignment of 28-12 and its UCA. Dots indicate identical residues. CDR regions according to IMGT are shown in red. **b**, **c** The reactivity of 28-12 germline variants to H3N2 (**b**) and H1N1 HAs (**c**). GHGL, V_H_ germline paired with V_L_ germline. HGL, 28-12 V_H_ paired with its V_L_ germline. GHL, 28-12 V_L_ paired with its V_H_ germline. **d** Heatmap showing the EC50 values of 28-12 variants with single somatic mutations to H3N2 and H1N1 HAs. **e** For both 28-12-H3 and 28-12-H1 complexes, V_L_ R53 forms hydrogen bonds with the main chain of V_H_ S103 in HCDR3, which also contacts the residue D46_HA2_ through hydrogen bonds. **f** For both 28-12-H3 and 28-12-H1 complexes, V_L_ D92 of 28-12 forms salt bridges with K39_HA2_ of helix A. **g** V_H_ F114 inserts into a hydrophobic groove formed by L38_HA2_ of both H3N2 and H1N1 HAs. The colors of HA1 and HA2 and the heavy chain and light chain of 28-12 are shown as in Fig. 4. The salt bridge and hydrogen bond are shown as red and black dashed lines, respectively. Data are presented as mean values of duplicates (**b**, **c**). Representative data are shown from two independent experiments (**b**, **c**).

### Role of somatic mutations in shaping antibody 28-12

To determine how the unmutated common ancestor (UCA) of 28-12 evolved to achieve cross-group neutralization with distinct recognition patterns, we analyzed the contribution of somatic mutations of 28-12 carrying 8 amino acid substitutions in V_H_ and 9 amino acid substitutions in V_L_ (Fig. 6a). We first expressed 28-12 germline versions (GHGLs) and versions formed by 28-12 light chains paired with germline heavy chains (GHLs) and vice versa (HGLs). The fully germline version (GHGL) of 28-12 reacted with a panel of group 2 H3N2 isolates but failed to recognize a panel of group 1 H1N1 isolates, indicating that the UCA antibody may have initially been selected by a H3N2 virus. 28-12 recognition required somatic mutations in both heavy and light chains, with the mutations in light chain dominating the contribution while heavy chain showing minor but indispensable roles (Fig. 6b, c). We then reverted the somatic mutations into germline sequences with single amino acid substitutions. The reversion of the somatic mutations at positions 32, 53, 92 (Y32W, R53S, and D92N) of the light chain and position 114 (F114A) of the heavy chain significantly decreased the binding activity to H3N2 and affected the potency to H1N1 (Fig. 6d).

We further elucidated the structural basis for the effects of somatic mutations. For both 28-12-H3 and 28-12-H1 complexes, V_L_ R53 forms hydrogen bonds with the main chain of V_H_ S103 in HCDR3, in the meanwhile, V_H_ S103 also contacts with residue D46_HA2_ through hydrogen bonds (Fig. 6e). Mutation of V_L_ R53 to germline residues may destabilize HCDR3 conformation, thus decreasing the reactivity to residue D46_HA2_ of both H3N2 and H1N1 HAs by 28-12. V_L_ D92 and V_H_ F114 are also within the antibody interface. Mutation of V_L_ D92 to germline residue N abolished the salt bridge with K39_HA2_ for both H1N1 and H3N2 (Fig. 6f). Moreover, V_H_ F114 forms important hydrophobic interactions with L38_HA2_ for both H3N2 and H1N1, which may be abolished by mutation of V_H_ F114 to A (Fig. 6g). Mutation of V_L_ Y32 to germline residue W is not involved in the binding interface and it may change the antibody conformation to decrease the reactivity to HAs.

These findings indicate that naive B cells that generated 28-12 could recognize H3N2 and that successional somatic mutations enhanced the reactivity to H3N2 and concurrently evolved the cross-reactivity to H1N1.

## Discussion

H1N1 and H3N2 IAVs circulate annually. Recently, coinfection with IAV was reported to enhance SARS-CoV-2 infectivity, thus posing another threat to public health during the COVID-19 pandemic^24–26^. HA stem region-directed bnAbs provide an option for antibody-based therapeutics against novel emerging IAVs from zoonotic origin and seasonal circulating IAV strains. In addition, universal vaccine design has also been inspired by the epitopes and recognition patterns of bnAbs. Structural analysis of antibody/HA complexes enhances our understanding of cross-group heterosubtypic binding features.

In this study, we identified a linage mAbs, 28-2, 28-4, 28-6 and 28-12 belonging to the same lineage V_H_3-48/D_H_2-2/J_H_6 and V_K_1-12/J_K_5, which is rarely reported. In addition to V_H_3-48, broad neutralizing antibodies using V_H_1-18, V_H_1-69, V_H_6-1 and V_H_3-30 germline genes have also been reported, which indicates the diverse germline selection of broad-spectrum antibodies against IAVs.

Their recognition pattern is also unique, as they mainly bind a continuous epitope within H3N2 HA, but a fully conformational epitope within H1N1 HA. The antigenic determinant differences, especially the N49_HA2_ (H3) / T49_HA2_ (H1) revealed distinct antigenicity of HA proteins between H1 and H3. The epitope dependency on the distant HA1 residues and the fusion peptide in the primary sequence probably makes the epitope in H1 HA more conformational by 28-12. Another reason might be attributed to the biophysical features of V_H_3-48/V_K_1-12 lineage antibodies. The long and flexible variable regions, especially the 23 amino acids of HCDR3, might adopt different conformations to fit structurally divergent antigens. HCDR3 residues 108Y and 109 V make hydrophobic interaction with L52_HA2_ (H3) or V52_HA2_ (H1). 108Y and 109 V also makes hydrophobic contacts with some HA1 residues V40, S299 and L300 of H1, but not H3. 106N_HCDR3_ forms hydrogen bond with N53_HA2_ in H1, and all N53_HA2_, N49_HA2_ and E57_HA2_ in H3. In addition, the observations of the differential binding of 28-12 to H1 and H3 cleaved HAs also indicate the distinct intrinsic properties of HAs within this epitope. We hypothesis that cleavage of H1 HA0 may cause conformational change and destroy the integrity of epitope in H1 HA, thus resulting in reduced binding activity to antibody 28-12. However, cleavage of H3 HA0 may not change the overall epitope structure, therefore the cleaved H3 HA still maintains the reactivity to 28-12. Thus, the epitope feature differences among distinct IAV subtypes should be carefully evaluated in the design of HA stem-based immunogens during universal influenza vaccine development.

Comparison of 28-12 with previously reported cross-group anti-HA stem bnAbs revealed that the helix A-targeting bnAbs bind HAs with overlapping but distinct binding sites and multiple approaching angles towards the HA stem region. The epitopes of antibodies CR6261, CT149, 39.29, CR9114 and FI6v3 all span HA1 and HA2 regions on one single protomer (for the primary protomer). 3I14 only binds HA2 of the primary protomer. However, here 28-12 shows different binding dependency on HA1 epitope residues, which is important for binding to H1 (HA1 residues V40, S299 and L300), but not H3 (Supplementary Figs. 9, 11 and Fig. 4c–e). The cross-binding on HA1 and HA2 regions on one protomer probably makes the epitope more conformation dependent. Consistent with the structural analysis, both the non-HA1/HA2 spanning mAbs (for one single protomer) 28-12 and 3I14 showed binding activity to the continuous epitope of H3 peptide 36-57_HA2_, but not to H1 (Supplementary Fig. 10). A critical reason for this unique binding feature might be attributed to the critical residue N49_HA2_ in H3 for both 28-12 and 3I14, which is T49_HA2_ in H1 (Fig. 6a). Of noting, 28-12 displayed different germline with 3I14 (V_H_3-30) and engaged less epitopes in the adjacent protomer (Supplementary Fig. 9). 28-12 is also unique as it has a long HCDR3 (23aa), which makes more extensive interaction with HA protein than other bnAbs. Although 3I14 also has a long HCDR3 (23aa), it seems that the HCDR3 of 28-12 is more significant than 3I14 to recognize HA as it contributes more amino acids to interact with H3N2 HA (12) and H1N1 HA (11), respectively, while 3I14 devotes 7 HCDR3 amino acids to interact with H3 and H6 HAs (Supplementary Fig. 7). Thus, 28-12 represents a unique example among the helix A targeting bnAbs. The HCDR3 of 28-12 might be developed as a potential template to produce small proteins or peptide-based antivirals as recently reported^27,28^.

We quantified the contribution of 28-12 lineage in broad-reactive B cells by analyzing four reported HA specific human antibody datasets^20,29–31^. Generally, 1.619% (17 out of 1050) cross-reactive B cells utilized V_H_3-48 germline genes among the four analyzed datasets. However, antibodies using both V_H_3-48 and V_K_1-12 germline genes are not found in the four analyzed datasets. From B cell repertoires of healthy donors with paired heavy chain and light chain using a dataset from DeKosky et al., which contained 134,345 sequences in total from three healthy donors^32^, B cells engaging V_H_3-48 and V_K_1-12 concurrently are rare with frequency ranging from 0.060% to 0.078%. We thus consider this as a special case in our study. We also found the 28-12 epitope-targeting antibodies in the serum of vaccinated human donors could be detected but exist as a relatively low frequency (Supplementary Fig. 12).

The recognition pattern of the antibodies to group 1 or group 2 IAVs embodies the flexibility of bnAbs against influenza A viruses: the continuous encounter of HA antigens with similar but slightly different B cell epitopes results in the in vivo evolution of V_H_3-48/V_K_1-12 lineage antibodies. Similar to previously reported models of V_H_6-1 and other lineages of bnAbs that developed from group 2-specific germline precursors^9,16^, we revealed that 28-12 UCAs showed group 2 viruses (H3) tendentious and subsequently enhanced their reactivity to group 2 viruses and acquired cross-reactivity to group 1 viruses (H1) by somatic mutations. These findings also reaffirmed the possibility of inducing cross-reactive antibodies via rationally designed sequential immunization with different HA immunogens.

In summary, the human antibody 28-12 itself is of high neutralizing potency and not polyreactive, therefore could serve as a potential antiviral drug for both prophylaxis and treatment of circulating H1N1 and H3N2 IAV infection. Additionally, the unique epitope targeted by 28-12 and the paratope of 28-12, especially the HCDR3 sheds some lights on the design of potential universal vaccines and more effective antiviral drugs against IAVs.

## Methods

### Ethics statement

The procedures in this study involving in the isolation of HA-specific memory B cells from the volunteer and the influenza virus-related experiments were approved by the Ethics Review Committee of Institut Pasteur of Shanghai, Chinese Academic of Sciences. The animal experiments in a biosafety level 2 (BSL-2) facility were approved by Institutional Animal Care and Use Committee at Institut Pasteur of Shanghai. The collection and study of the 50 vaccinated donors’ sera were approved by the Ethical Review Committee of National Institute for Viral Disease Control and Prevention, China CDC. All manipulations were strictly conducted in compliance with animal ethics guidelines and approved protocols.

### Viruses and cells

The wild-type influenza viruses A/Sichuan/1/2009 (H1N1), A/California/07/2009 (H1N1), A/duck/Hunan/8-19/2009 (H4N2), and A/Jiangxi-Donghu/312/2006 (H3N2) and the recombinant influenza viruses A/Shanghai/02/2013 (HA, NA) x A/Puerto Rico/8/34 (H7N9) and A/HongKong/01/1968 (HA, NA) x A/Puerto Rico/8/34 (H3N2) were all grown on Madin-Darby canine kidney (MDCK) cells, while A/canine/Beijing/362/2009 (H3N2) was amplified in embryonated eggs. These viruses were used in the in vitro microneutralization assay. The A/Hong Kong/01/1968 (HA, NA) x A/Puerto Rico/8/34 (H3N2) and A/Sichuan/1/2009 (H1N1) viruses were used in the in vivo assay. MDCK, HeLa and HEK293T cells were obtained from the American Type Culture Collection (Manassas, VA, USA) and cultured in Dμlbecco’s modified Eagle’s medium (DMEM, Gibco) with 10% foetal bovine serum (FBS). CHO cells (Thermo Fisher Scientific) were cultured in Expi™ CHO expression medium.

### Recombinant HA proteins

The gene encoding the trimeric ectodomains of the HK/68 H3 and WA/11 H1 HA proteins with a T4 fibritin trimerization motif in the C-terminus were expressed in a Bac-to-Bac Baculovirus Expression System (Invitrogen) and used for structural study. HA proteins were purified by affinity chromatography using a His trap Excel column (GE, EDTA-resistant) and size-exclusion chromatography using a Superdex 200 10/300 column (GE Healthcare).

The following recombinant HAs expressed either from baculovirus or mammalian expression systems were all utilized for the binding affinity assay: A/Hong Kong/01/1968 (H3N2) HA (Sino Biological, cat. no. 40116-V08B), A/Netherlands/219/2003 (H7N7) HA (Sino Biological, cat. no. 11082-V08B), A/Brevig Mission/1/1918 (H1N1) HA (Sino Biological, cat. no. 11068-V08H), A/Florida/04/2017 HA (Sino Biological, cat. no. 40683-V08H), A/WSN/1933 (H1N1) HA (Sino Biological, cat. no. 11692-V08H), A/chicken/Guangdong/C273/2011 (H6N2) HA (Sino Biological, cat. no. 40398-V08B), A/pintail duck/Alberta/114/1979 (H8N4) HA (Sino Biological, cat. no. 11722-V08B), A/Hong Kong/1073/1999 (H9N2) HA (Sino Biological, cat. no. 40040-V08B), A/Missouri/09/2014 (H3N2) HA (Sino Biological, cat. no. 40494-V08B), A/Netherlands/178/1995 (H3N2) HA (Sino Biological, cat. no. 40486-V08B), A/chicken/Alabama/01/1975 (H4N8) HA (Sino Biological, cat. no. 40025-V08B), A/mallard duck/Alberta/299/1977 (H4N4) HA (Sino Biological, cat. no. 40008-V08B), A/Mallard/Astrakhan/263/1982 (H14N5) HA (Sino Biological, cat. no. 40192-V08B) A/California/06/2009 (H1N1) HA (Sino Biological, cat. no. 11055-V08B).

### The isolation of HA-specific memory B cells

This study was approved by the Ethics Review Committee of Institut Pasteur of Shanghai, CAS (IPS-2016004). The informed consent was obtained by the participant. Blood was collected from a female volunteer previously ~ 4 weeks after inoculating with seasonal split influenza vaccine after she had signed the informed consent form. The vaccine was produced by Shanghai Institute of Biological Products Co., Ltd. in 2016, which contains three components, A/California/7/2009(H1N1) pdm09-like virus, A/Victoria/361/2011(H3N2)-like virus and B/Wisconsin/1/2010-like virus. Fresh PBMCs were isolated from the collected blood by using the Ficoll-Paque gradient (GE Healthcare, cat. no. 17144002). The PBMCs were stained with a FITC-labeled mouse anti-human CD19 antibody (BD Pharmingen^TM^, cat. no. 555412) and an APC-labeled mouse anti-human IgG antibody (BD Pharmingen^TM^, cat. no. 550931). The recombinant HK/68 H3N2 HA protein was labeled with biotin using the EZ-Link Sμlfo-NHS-LC Biotin biotinylation reagent (Thermo Fisher Scientific, cat. no. 21335), which could bind to the streptavidin-Cy3 conjugate (Sigma-Aldrich, cat. no. S6402). Single FITC-CD19^+^/APC-IgG^+^/streptavidin-Cy3 conjugate-HA-specific memory B cells were isolated with a BD Influx^TM^ Cell Sorter and sorted into 96-well plates. The data were analyzed by FlowJo V10 software.

### Cloning, expression and purification of antibodies

The amino acid sequences of heavy and light chain variable regions of MEDI8852 (5JW4^9^), CR8020^23^ (3SDY), CR9114 (4FQY^13^), CT149 (4UBD^15^), 39.29 (4KVN^14^), FI6v3 (3ZTJ^16^), 3I14 (6WF0^33^), S139/1^34^ (4GMS) and 5j8^35^ (GenBank: JF791168: and JF791169) were downloaded from PDB or NCBI GenBank, codon optimized in mammalian cells and synthesized by Shanghai Generay Biotech. The cloning of the IgG1 V_H_ and V_K_ chain expression plasmids was previously described^36^. The plasmids encoding the V_H_ and V_K_ chain of antibodies were cotransfected into Expi™ CHO cells, and cultured according to the manufacturer’s instructions (Thermo Fisher Scientific, cat. no. A29129). Antibodies were purified from the Expi™CHO cell supernatants using Protein A Agarose (MabSelect Prism A, GE Healthcare).

### Microneutralization assay

The microneutralization assay was performed as previously described^36^. Briefly, MDCK cells were seeded into 96-well plates the day before the experiment, washed twice with PBS, and then incubated in DMEM supplemented with 2 µg/ml trypsin (Sigma-Aldrich, cat. T1426) on the day of the experiment. Antibodies were serially diluted twofold in 50 µl DMEM and then mixed with 100 TCID50 of influenza virus in 50 μl DMEM for 1 h at 37 °C in a cell culture incubator. Positive control wells contained viruses only, while negative control wells contained no viruses. One hour later, the virus-antibody mixture was transferred to MDCK cells and incubated for 24 h at 37 °C in a 5% CO_2_ humidified atmosphere. After 24 h of infection, the virus-antibody mixture was removed, the cells were washed with PBS and fixed in 80% acetone for 30 min, and viral antigen was detected by ELISA with a polyclonal antibody against NP protein (prepared in our lab) as previously used^12^. The optical density at 450 nm (OD450) was recorded with a plate reader (Bio-Tek Epoch 2). The half maximal inhibitory concentration (IC50) was analyzed with GraphPad Prism 7.0.

### Protective efficacy of 28-12 in mice

The animal experiments described in this study were approved and in compliance with the BSL-2 laboratory guidelines of the Institut Pasteur of Shanghai, CAS. For the prophylaxis experiment, 7-week-old female BALB/c mice (*n* = 5) received 1, 3, 10, or 30 mg/kg 28-12 or PBS buffer via the intraperitoneal (i.p.) route in a 0.2 ml volume 24 h before intranasal challenge with a lethal dose of A/Sichuan/1/2009 (H1N1) or A/Hong Kong/01/1968 (H3N2) virus, respectively. For the therapeutic experiment, the mice (*n* = 5) were intranasally infected with a lethal dose of viruses 24 h before i.p. administration of 25 mg/kg 28-12 at 0, 1, 2 and 3 days post infection. Mice were weighed and monitored daily for 14 days after influenza virus infection.

### HA conformational change inhibition assay

To activate HA0 into HA1 and HA2, HK/68 H3 HA-histidine protein (Sino Biological) was mixed with trypsin in PBS buffer at room temperature for 2 h. Trypsin activity was stopped by adding aprotinin (Sigma, cat. A1153). The above mixture was divided into 3 groups and incubated with 28-12, the anti-HCV antibody 8D6, or no antibody in PBS buffer at 37 °C for 2 h. Samples were treated with PBS or buffer with low pH (100 mM sodium acetate, 1% n-dodecyl β-D-maltoside (Sigma-Aldrich, cat. no. D4641), pH = 4.8) at 37 °C for 1 h through buffer exchange using Zeba™ Spin Desalting Plates, 7 K MWCO (Thermo Fisher Scientific, cat. 89882). Samples were neutralized with Tris-HCl (pH 9.0) or PBS to a pH of 8.0. The above samples were mixed with 0.0025 μg trypsin individually and incubated at room temperature for 1 h. Samples were then run on a 10% SDS–polyacrylamide electrophoresis gel under nonreducing conditions and blotted using an HRP-conjugated 6x His mouse monoclonal antibody (Proteintech, Cat No. HRP-66005). A GE healthcare ImageQuant LAS4000 mini biomolecular imager was used for the detection and quantitation of chemiluminescence. The conformational change inhibition assay of the SC/09 H1 HA protein (Sino Biological) was performed as described above.

### Membrane fusion inhibition assay

HeLa cells in 12-well plates were transfected with FΜGW plasmids encoding the HA gene fragment of the HK/68 H3 or SC/09 H1 strain using Lipofectamine^TM^2000 transfection reagent. Twenty-four hours after transfection, the HeLa cells were incubated with 28-12, 8D6 (100 μg/ml) or no antibody at 37 °C for 1 h. The supernatants were removed, and the cells were treated with trypsin (2.5 μg/ml) for 10 min at 37 °C to cleave HA0 into HA1 and HA2. After washing twice, the cells were incubated with 28-12, 8D6 or no antibody at 37 °C for 1 h. Afterwards, the supernatants were removed, and the cells were treated with citric acid (pH 4.8) at 37 °C for 10 min. The acidic medium was replaced with DMEM supplemented with 10% FBS, and the cells were incubated for 3 h, followed by 4% polyoxymethylene fixation and staining with 0.5% crystal violet. Syncytium formation was observed with the Olympus IX73 inverted microscope and the images were collected by Olympus cellSens Entry software.

### ELISA

The binding affinity of antibodies to HAs was measured by ELISA. The HA protein (0.5 μg/ml in 100 μl/well) was captured onto a 96-well plate (Thermo Fisher Scientific, cat. no. 446469) overnight. The wells were blocked with 1% bovine serum albumin in PBS at 37 °C for 2 h. Antibodies were serially diluted threefold in blocking buffer (starting from a concentration of 10 μg/ml) and were incubated in the wells at 37 °C for 2 h. The samples were washed three times, and an anti-human Fc HRP antibody (Sigma-Aldrich, cat. no. A0170) was used to detect the binding affinity, followed by incubation with tetramethylbenzydine (Beyotime, cat. P0209), and the reaction was stopped by adding 1 M HCl. The absorbance at 450 nm was recorded by a plate reader (Bio-Tek Epoch 2), and the data were analyzed with GraphPad Prism 7.0 software.

To evaluate the reactivity between antibodies and peptides, peptides (0.5 μg/ml in 100 μl/well) were captured onto 96-well plates, and ELISA was performed as described above.

The antibody binding activity to untreated or denatured HA was measured as described above. The HA protein was denatured with 0.1% SDS, 50 mM DTT and a metal bath at 100 °C for 10 min.

### Biolayer interferometry (BLI) analysis

To test the binding affinity between 28-12 and different HA proteins, 28-12 (15 µg/mL) was immobilized on an anti-human IgG-Fc-coated biosensor (AHC) surface for 300 s. The baseline interference phase was then read for 180 s in kinetics buffer (KB: 1× PBS), followed by subsequent association phase immersion of the sensors into wells containing two-fold serial dilutions of HA protein (100-50-25-12.5-6.25-3.125 nM) in KB for 600 s. Then, the sensors were immersed in KB for dissociation for up to 600 s. The mean *K*_on_, *K*_off_ and apparent *K*_D_ values were calculated using the global fit to a 1:1 Langmuir binding model with an R^2^ value of ≥0.95. The data were collected using OCTET DATA acquisition 9.0 and the data were analyzed by Fortebio Data Analysis 9.0.

### HA mutagenesis and binding measurements

The DNA sequences encoding the extracellular domain of HA proteins of HK/68 H3 or WA/11 H1 were fused to a C-terminal 8X His tag. Site-directed mutagenesis was induced with a commercialized KOD-Plus mutagenesis kit (TOYOBO). The plasmids were transfected into CHO cells in 12-well plates. The supernatants were harvested 96 h after transfection. ELISA was performed to measure expression of individual HA proteins in the cell supernatant. In brief, the HA head antibody 139/S (H3) or 5j8 (H1) was coated onto a plate, and 20-, 100-, 500-fold dilutions of cell supernatant were then added and captured by the anti-6 X His antibody. Serially diluted purified HA-8 X His was used as a standard, followed by detection with an HRP-conjugated mouse anti-His antibody (Proteintech). The concentration of the sample was calculated according to a standard curve. Another ELISA was performed to analyze the relative binding activities between these HA mutants and 28-12. HA mutants at 200 ng/ml were incubated with plates precoated with 28-12, followed by detection with an HRP-conjugated mouse anti-6 X His antibody. The binding activity of the mutants to 28-12 was compared to that of wild-type HA.

### Preparation of 28-12 Fab

Papain (Sigma-Aldrich, cat.no. P4762) was dissolved in PBS with 20 mM L-cysteine to activate papain. Antibody 28-12 was digested with papain at an antibody-to-papain ratio of 200:1 at 37 °C for 6 h. Protease activity was stopped by iodoacetamide (Sigma-Aldrich, cat. no. I6125). 28-12 was purified using protein A to remove the Fc region and undigested IgG antibody, followed by 2–3 rounds of dialysis using PBS at 4 °C for 6 h. Size-exclusion gel filtration chromatography was performed to further purify 28–12 Fab.

### Preparation of 28-12 Fab/trimeric HA complexes

Trimeric HK/68 H3 and WA/11 H1 HAs were expressed in the insect baculovirus expression system, while 28-12 was expressed in the Expi^TM^CHO expression system, as described previously^12^. Fab 28-12 was mixed with trimeric HA at a Fab-to-monomer HA molar ratio of 2:1 and incubated at 4 °C overnight for complex formation. The Fab-trimeric HA complex was purified by size-exclusion gel filtration chromatography in elution buffer (20 mM Hepes, 50 mM NaCl, pH 8.0), followed by concentrating the complex to 5–6 mg/ml.

### Cryo-EM sample preparation and data acquisition

To prepare vitrified samples, the purified 28-12-H1 or 28-12-H3 complex was diluted to 0.5 mg/ml, and a 2 µl aliquot of sample was applied to a plasma-treated holey carbon grid (Quantifoil, R1.2/1.3, 200 mesh). The grid was blotted with a Vitrobot Mark IV (Thermo Fisher Scientific) and then plunged into liquid ethane cooled by liquid nitrogen.

The movie stacks for both complexes were collected on a Titan Krios electron microscope (FEI TEM user interface 2.15.3, Thermo Fisher Scientific) operated at an acceleration voltage of 300 kV, with a nominal magnification of 18,000× (yielding a pixel size of 1.318 Å; Supplementary Table 1). The movies were recorded on a K2 Summit direct electron detector (Gatan) operated in super resolution mode under low-dose conditions in an automatic manner using SerialEM^37^. Each frame was exposed for 0.2 s and the total accumulation time was 7.6 s, leading to a total accumulated dose of 38 e^–^/Å^2^ for the specimen.

### Cryo-EM data processing and 3D reconstruction

Single-particle analysis was mainly executed in Relion^38^ unless otherwise specified. All images were aligned and summed using MotionCor 2 software^39^. After CTF parameter determination using CTFFIND^40^, particle autopicking, manual particle checking, and reference-free 2D classification, particles with 28-12-H3 trimer features remained for further processing.

For the 28-12-H3 dataset, 240,827 and 180,686 particles remained after 2D classification for the non-tilt and 40°-tilt micrographs, respectively (Supplementary Fig. 4a). For the tilted data, we used goCTF software to perform per-particle CTF estimation to determine their defocus, and these particles were re-extracted with corrected defocus^41^. We then combined the particles together and performed one round of refinement. After that, we re-extracted the particles coordinating the refinement result to recenter the particles. We then performed 3D classification, and a class of 191,540 particles with better structural features was selected. After refinement, CTF refinement and Bayesian polishing, the map was refined to 4.1-Å-resolution with imposed C3 symmetry. We then carried out 3D classification, generating a better class with 105, 383 particles. After refinement, we obtained a 3.7-Å-resolution 28-12-H3 map. For this and the following 28-12-H1 cryo-EM maps, the post-process was carried out by DeepEMhacer^42^, and the resolution estimation was based on the gold-standard Fourier Shell Correlation (FSC) 0.143 criterion, and the local resolution was estimated using Resmap^43^.

For the 28-12-H1 dataset, 403,055 particles remained after 2D classification (Supplementary Fig. 5a). After refinement, we re-extracted and recenter the particles, subsequently performed multiple rounds of 3D classification, leading to a better class with 61% of the particles. We then carried out heterogeneous refinement in cryoSparc^44^ and a better class with 124,947 particles was selected. We then performed nonuniform refinement in CryoSPARC. After CTF refinement and Bayesian polishing, the structure was refined to 4.1-Å-resolution with imposed C3 symmetry in Relion. We then performed the nonuniform refinement in CryoSPARC using polished particles and obtained a 3.5-Å-resolution cryo-EM map of 28-12-H1.

### Atomic model building

We first built the homology model for the 28-12 Fab through the SWISS-MODEL server^45^. Since the heavy chain of 28-12 has a particular long CDR3, we used the heavy chain of the VRC 31513-1b02 antibody (PDBID: 5TY6)^30^ as template, which has a similar CDR3 sequence and shares 83% sequence identity with that of 28-12. For the light chain of Fab 28-12, we used the light chain of the complement C5 antibody (PDBID: 5B71)^46^ as template, which shares 92% sequence identity with that of 28-12.

To generate the atomic model of the 28-12-H3 complex, we first fitted the HK68/H3 X-ray structure (PDBID: 3SDY)^47^ and the homology model of 28-12 Fab into the 28-12-H3 cryo-EM map as a rigid body using *fit in map* module in UCSF Chimera^48^. Afterwards, we refined the model against the corresponding cryo-EM map in Rossetta^49^, then Phenix^50^. Furthermore, to improve the fit between the model and map, we performed real space refinement using COOT^51^. Finally, we used Phenix again for the last round of flexible fitting of the entire complex. For the 28-12-H1 complex, a similar flexible-fitting procedure was applied, except that we used WA11/H1 X-ray structure (PDBID: 5gjt)^12^ to fit the H1 trimer structure.

We used UCSF Chimera and ChimeraX for generating figures and performing electrostatic surface property calculations^52,53^. Interaction surface analysis was conducted by using the PISA server^54^.

### Affinity depletion of anti-H3 peptide 36-57_HA2_ antibodies

The collection and study of the 50 vaccinated donors’ sera were approved by the Ethical Review Committee of National Institute for Viral Disease Control and Prevention, China CDC. The informed consent was obtained by the participants. Selected synthetic biotinylated H3 peptide 36-57_HA2_ (Genescript) were added at 150 ng/well to streptavidin-coated plates and incubated at room temperature for 1 h in PBS containing 0.1% Tween-20. The sera were added into the plates and incubated for 20 min at RT for absorption. The unbound antibodies were collected after 24 rounds of absorption. Adsorbed samples were then mixed with HK/68 H3N2 virus for neutralization assay. A non-specific peptide control, OVA, was also included.

### Polyreactivity of antibodies

To detect the polyreactivity of mAbs, the ELISA and immunofluorescence-based assays were performed as previously described^20^. For ELISA-based detection of polyreactivity, plates were coated with poly I:C (15 mg/ml) (Life Technologies), LPS (15 mg/ml) (Sigma), or recombinant human insulin (10 mg/ml) (Sino Biological) in carbonate buffer. Plates were blocked with PBS with 0.05% Tween. Antibodies were serial diluted and incubated on the plate for 1 h at 37 °C followed by detection with goat anti-human IgG Fc at 1:8000. Absorbances were measured at OD450. An influenza antibody CR9114 and an anti-dsDNA antibody 3H9 was used as a positive control. For immunofluorescence-based detection of polyreactivity, HEp-2 cells were plated the day before experiment. HEp-2 cells were fixed with 4% PFA for 10 min followed by permeabilization with 0.3% Triton X-100 in PBS for 15 min. Biotinylated antibodies were incubated with cells for 1 h at the concentration of 50, 10 and 2 μg/ml, respectively and a FITC-linked streptavidin was then incubated with the cells for 1 h. The immunofluorescence was read and analyzed by Operetta (PerkinElmer).

### Reporting summary

Further information on research design is available in the Nature Research Reporting Summary linked to this article.

## Supplementary information

Supplementary information

Peer Review File

Reporting Summary

## Data Availability

All data presented in this study are available within the figures and in the Supplementary Information. Cryo-EM maps for the H3-28-12 complex and H1-28-12 complex have been deposited at the Electron Microscopy Data Bank with accession codes EMD-33023, and EMD-33024, respectively. Associated atomic models have been deposited in the Protein Data Bank with accession codes 7X6L and 7X6O for H3-28-12 and H1-28-12, respectively. The sequences used in Fig. 5 for analysis were: H3 A/HongKong/01/1968 (UniProtKB/Swiss-Prot: Q91MA7.1), H7 A/Netherlands/219/2003 (GenBank: AAR02640.2), H4 A/swine-ontario/01911-1/1999 (GenBank: AAG17429.1), H14 A/Mallard/Astrakhan/263/1982 (UniProtKB/Swiss-Prot: P26136.1), H15 A/duck/AUS/341/1983 (GenBank: ABB88132.1), H1 A/California/06/2009 (GenBank: ACP41105.1) and H10 A/Jiangxi-Donghu/346/2013 (GISAID Accession:EPI497477, https://platform.epicov.org/epi3/frontend#412409). Source data are provided with this paper.

## Source data

Source data
