# Supplementary material for: Unique binding pattern for a lineage of human antibodies with broad reactivity against influenza A virus
Source: Nat Commun. 2022 May 2;13:2378. doi: 10.1038/s41467-022-29950-w (PMC9061721; doi:10.1038/s41467-022-29950-w)
Supplement: Supplementary file 1 — Supplementary information [file 41467_2022_29950_MOESM1_ESM.pdf]

Supplementary information

Unique binding pattern for a lineage of human antibodies with broad reactivity against influenza A virus

Xiaoyu Sun, Caixuan Liu, Xiao Lu, Zhiyang Ling*, Chunyan Yi, Zhen Zhang, Zi Li, Mingliang Jin, Wenshuai Wang, Shubing Tang, Fangfang Wang, Fang Wang, Sonam Wangmo, Shuangfeng Chen, Li Li, Liyan Ma, Yaguang Zhang, Zhuo Yang, Xiaoping Dong, Zhikang Qian, Jianping Ding, Dayan Wang*, Yao Cong* and Bing Sun*

*Correspondence: Zhiyang Ling (lingzhiyang@sibs.ac.cn), Dayan Wang (wangdayan@ivdc.chinacdc.cn), Yao Cong (cong@sibcb.ac.cn) and Bing Sun (bsun@sibs.ac.cn).

Supplementary information

1. Supplementary Figure 1 (page 2)

2. Supplementary Figure 2 (page 3)

3. Supplementary Figure 3 (page 4)

4. Supplementary Figure 4 (page 5)

5. Supplementary Figure 5 (page 6)

6. Supplementary Figure 6 (page 7)

7. Supplementary Figure 7 (page 8)

8. Supplementary Figure 8 (page 9)

9. Supplementary Figure 9 (page 10)

10. Supplementary Figure 10 (page 11)

11. Supplementary Figure 11 (page 11)

12. Supplementary Figure 12 (page 12)

13. Supplementary Table 1 (page 13)

14. Supplementary Table 2 (page 14)

15. Supplementary Table 3 (page 15)

16. Supplementary Table 4 (page 16)

17. Supplementary Table 5 (page 17)

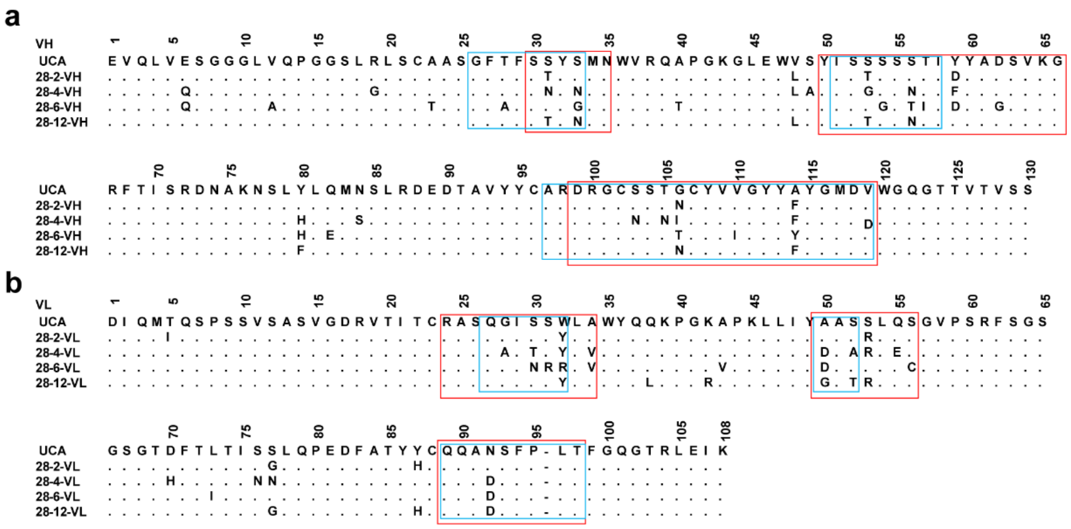

Supplementary Figure 1. Sequence alignment of V_H3-48/V_K1-12 lineage antibodies and the UCA. Alignment of the variable region of heavy chain (V_H, **a**) and light chain (V_L, **b**) amino-acid sequences of V_H3-48/V_K1-12 lineage antibodies with their unmutated common ancestor (UCA). Residue positions are numbered in order. Dots indicate identical residues. Boxes indicate CDR borders according to IMGT (blue line) and Kabat (red line).

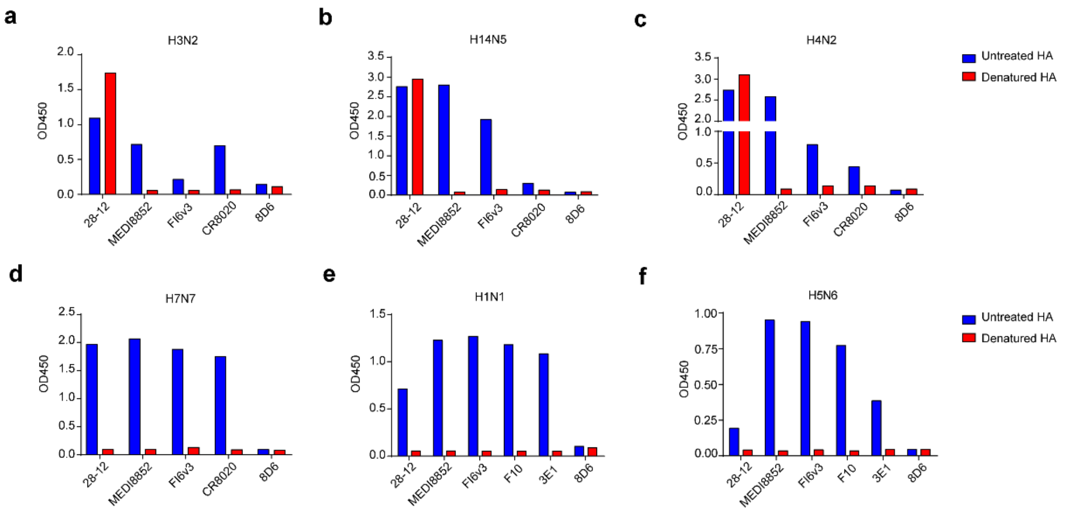

Supplementary Figure 2. Antibody 28-12 mainly recognizes a continuous epitope in H3 clade HAs, but a conformational epitope in H7 clade and group 1 subtypes. a-f, The binding activity of antibody 28-12 to untreated or denatured HAs of group 2 H3 clade IAVs: A/HongKong/01/1968 H3N2 **(a)**, A/Mallard/Astrakhan/263/1982 H14N5 **(b)** and A/duck/Hunan/8-19/2009 H4N2 **(c)**; group 2 H7 clade IAV: A/Netherlands/219/2003 H7N7 **(d)**; group 1 IAVs: A/California/06/2009 H1N1 **(e)** and A/Sichuan/26221/2014 H5N6 **(f)**, as measured by ELISA. The denatured HA protein was prepared with a metal bath at 100°C for 10 min before incubation with 0.1% SDS and 50 mM DTT. Results are depicted as the mean values from duplicates examined over two independent experiments.

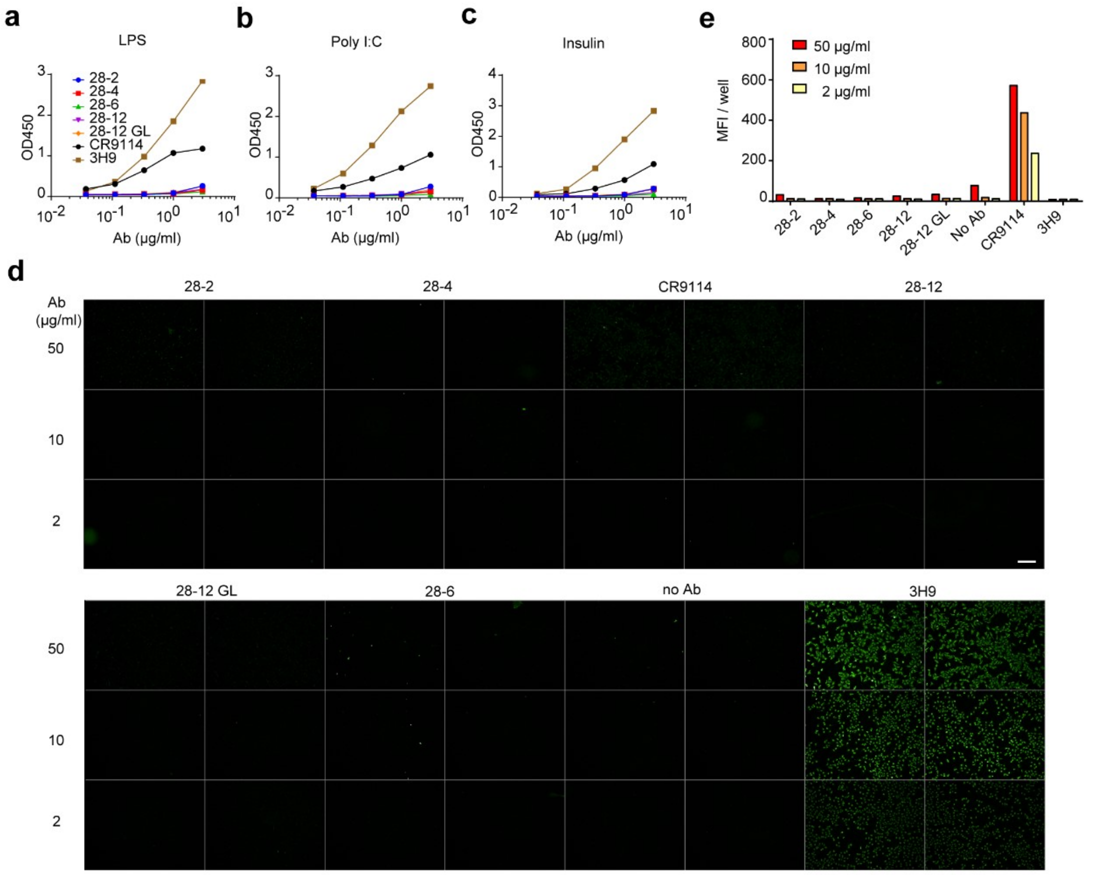

Supplementary Figure 3. VH3-48/VK1-12 mAbs are not polyreactive. ELISA-based binding curves of each mAb to antigens LPS **(a)**, Poly I:C **(b)** or insulin **(c)**. 28-12 GL, the germline version of 28-12. CR9114, a reported influenza HA stem mAb with polyreactivity to above antigens. 3H9, a high-affinity anti-nuclear antibody 3H9. **d,** Binding image of the each mAb at a concentration of 2, 10 or 50 μg/ml to HEp-2 cells by immunofluorescence. Scale bar, 200 μm. **e,** Mean fluorescence intensity (MFI) of each mAb binding to HEp-2 cells by immunofluorescence. Data are presented as mean values of duplicates (**a-c,e**). Representative data are shown from two independent experiments (**a-e**).

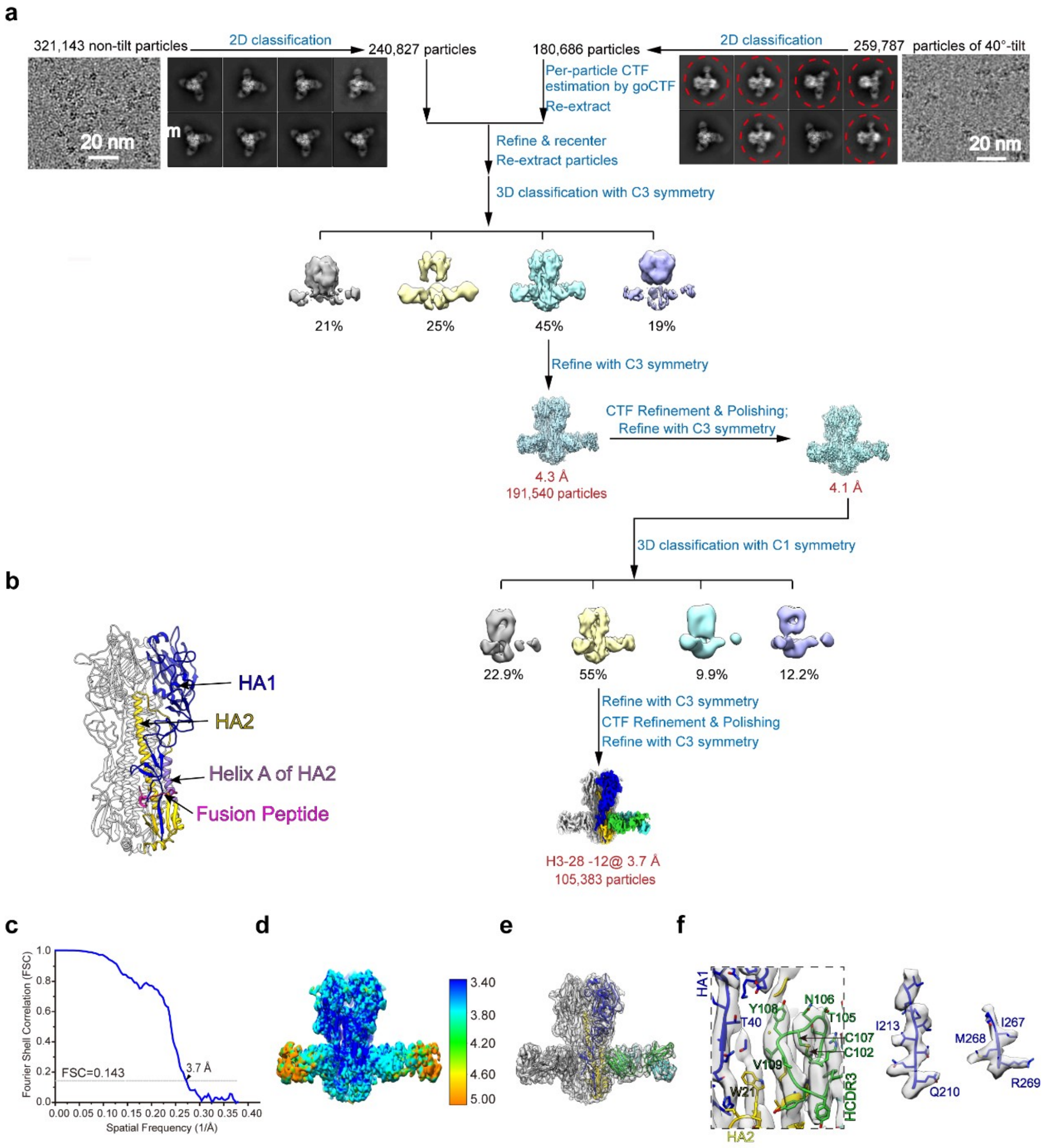

Supplementary Figure 4. Workflow for cryo-EM data processing for the 28-12-H3 complex. a, Cryo-EM data processing procedure for 28-12-H3. The representative cryo-EM micrograph and reference-free 2D class averages for non-tilt and 40°-tilt data are shown. Side views of the 28-12-H3 complex in the 2D class averages of the 40°-tilt data are indicated by red dashed circles. **b**, Structural element for HA. **c-d**, Resolution estimation of H3-28-12 according to the gold-standard FSC criterion of 0.143 (c), and local resolution evaluation (d). **e**, Good match between the atomic model and the map of H3-28-12. **f**, Representative high-resolution structural features of the 28-12-H3 complex.

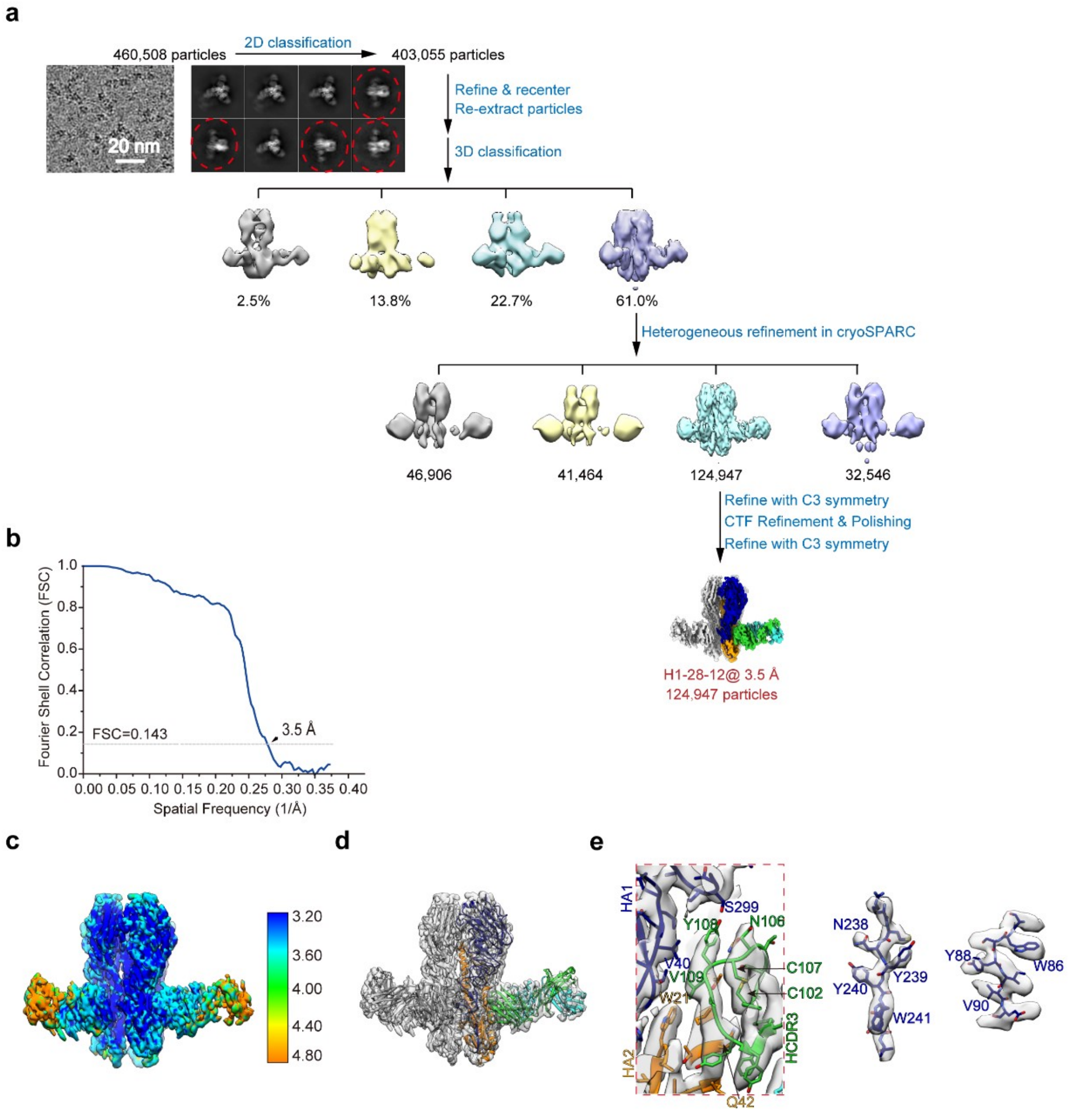

Supplementary Figure 5. Workflow for cryo-EM data processing for the 28-12-H1 complex. a, Cryo-EM data processing procedure for 28-12-H1. The representative cryo-EM micrograph, reference-free 2D class averages are shown. Side views of 28-12-H1 in the 2D class averages are indicated by red dashed circles. **b-c**, Resolution estimation of 28-12-H3 according to the gold-standard FSC criterion of 0.143 (b), and local resolution evaluation (c). **d**, Good match between the atomic model and map of H1-28-12. **e**, Representative high-resolution structural features of the 28-12-H1 complex.

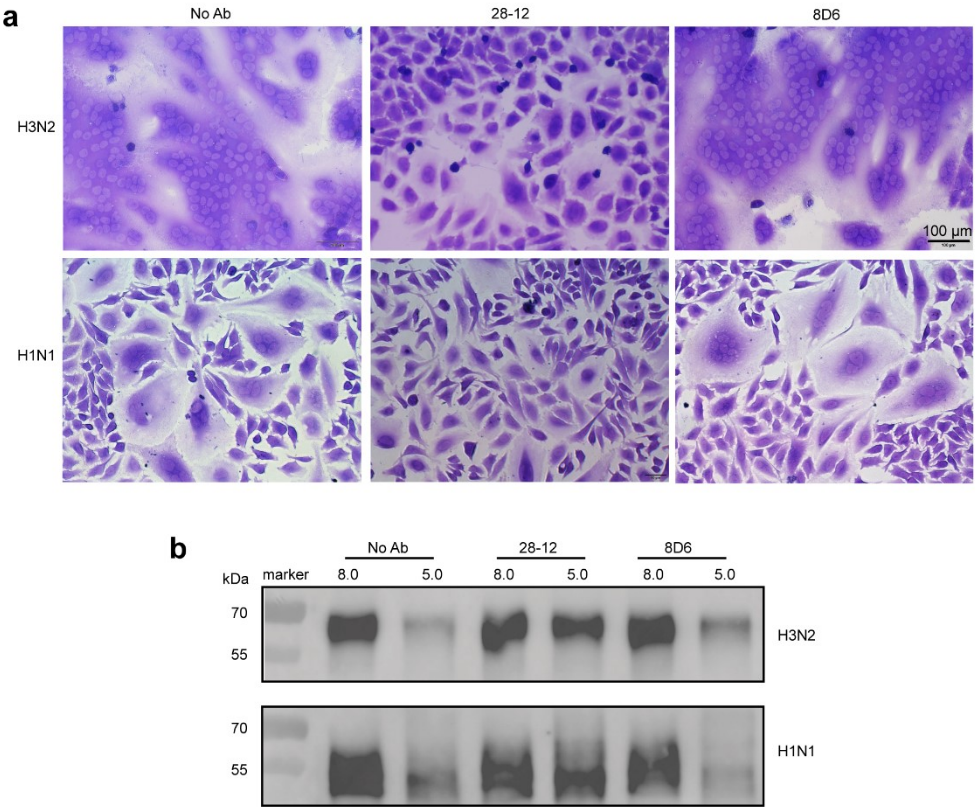

Supplementary Figure 6. 28-12 inhibits the low-pH induced HA conformational change. a, 28-12 blocks trypsin-activated, low pH-triggered, HA-mediated cell-cell fusion. HeLa cells were transfected with plasmids encoding full-length H3 or H1 HAs and treated with trypsin, and then 28-12 or isotype control mAb (8D6) was added, followed by low-pH treatment. Scale bar, 10 µm. **b**, Protease sensitivity assay of HA in the presence of 28-12. Exposure of HA to low pH converts HA to a protease-susceptible, postfusion state (lanes 2, 6). Treatment of HA with 28-12, but not the control mAb, before low-pH treatment blocks the pH-induced conformational change, retaining HA in the protease-resistant, prefusion state (lane 4). Representative data are shown from two independent experiments (**a,b**).

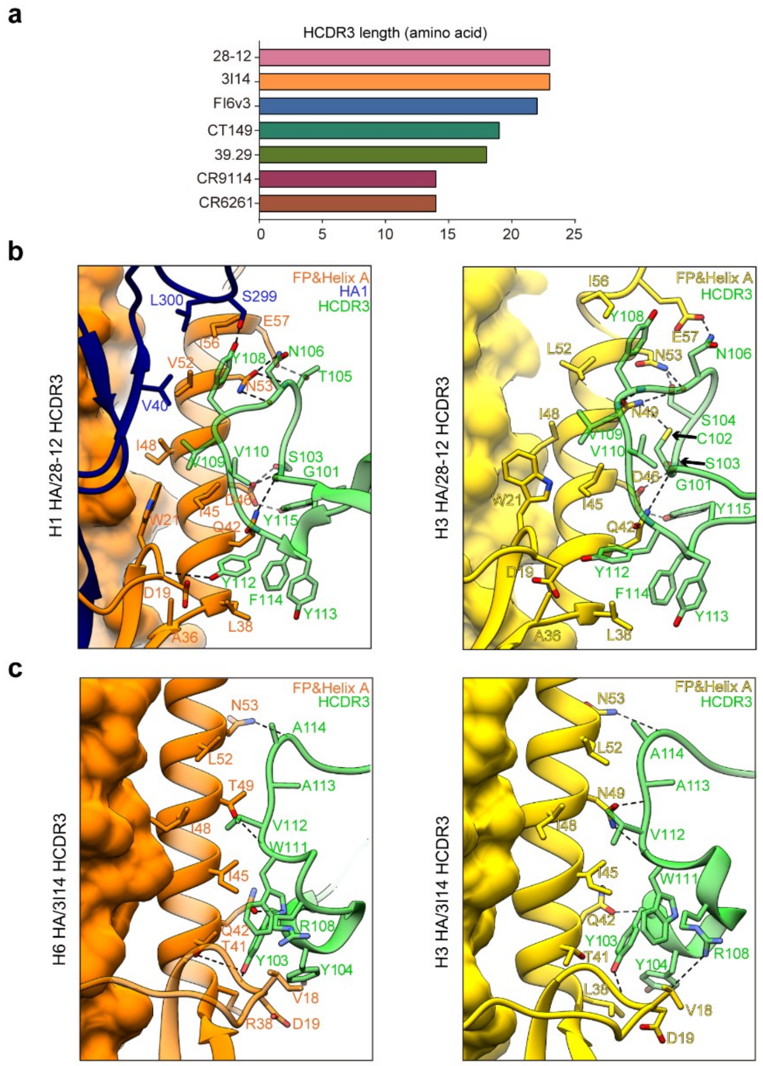

Supplementary Figure 7. The interaction network between HA and the HCDR3 of 28-12 or 3I14. a, The HCDR3 length of the helix A-targeting bnAbs. **b,** The interaction network between HCDR3 of 28-12 and group 1 H1 or group 2 H3 HAs. **c,** The interaction network between HCDR3 of 3I14 and group 1 H6 or group 2 H3 HAs. HCDR3 is shown in cartoon and colored in green. HA1 is shown in dark blue. The fusion peptide and helix A (HA2) of group 1 and group 2 is colored in orange and gold, respectively. Residues that make either hydrogen bonds or hydrophobic interactions are shown as sticks. Dashed lines indicate hydrogen bonds.

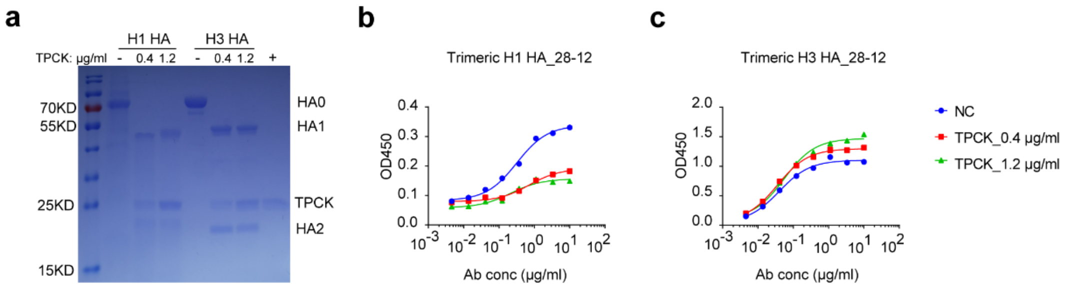

Supplementary Figure 8. Differential binding activity of 28-12 to the cleaved H1 and H3 HAs. **a,** SDS-PAGE analysis of HA0 and the cleaved HA proteins of H1 and H3 subtypes. Trimeric HA0 proteins (2 μg/ml, 100 μl) were treated with TPCK (0.4 or 1.2 μg/ml) for 15min at room temperature. The cleavage was stopped by adding protein loading buffer and a metal bath at 100°C for 10 min. **b- c,** ELISA-based binding activity of 28-12 to HA0 or cleaved HA of H1N1 **(b)** or H3N2 **(c)**. HA0 proteins (200 ng/well, 100 μl) coated onto the ELISA plates were treated with TPCK (40 or 120 ng/well, 100 μl) for 15min at room temperature. After blocking with 1%BSA and washing three times with PBST, the plates were incubated with serial diluted antibodies, followed by detection with an anti-human IgG Fc-HRP antibody. Results are presented as mean values of duplicates. Representative data are shown from two independent experiments (**b-c**).

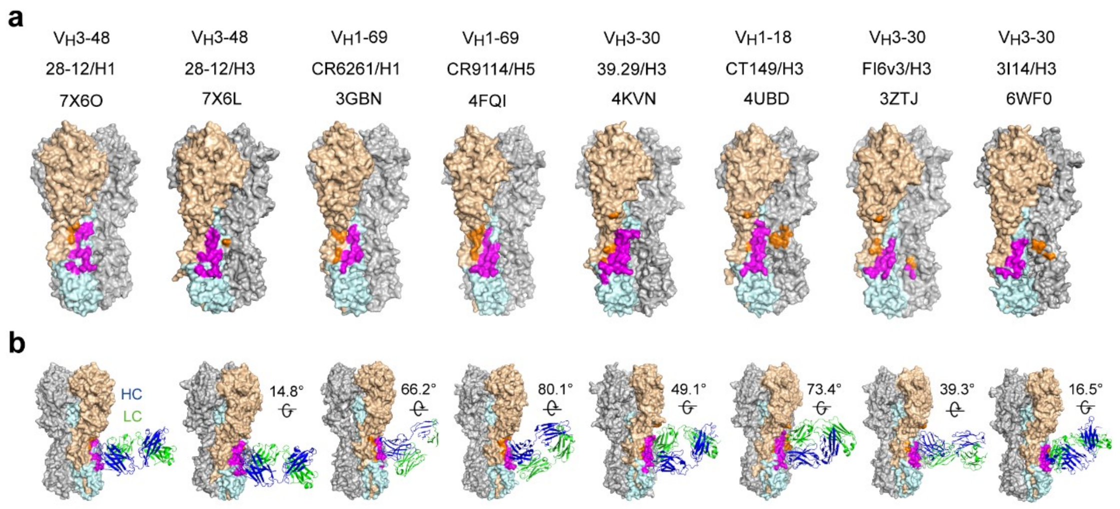

Supplementary Figure 9. Comparison of epitope and approaching angle for bnAbs that interact with Helix A. a, The epitope of Helix A-targeting bnAbs on the HA. HA is displayed in surface representation with HA1 and HA2 from the primary protomer in wheat and light blue, respectively. The epitope residues in HA1 are colored in orange and those in HA2 are colored in magenta. The color scheme is followed throughout. The V_H germline of each bnAb is also listed. **b,** The change of the Fab approaching angle compared with that of 28-12/H1. Each Fab is shown in cartoon representation with the heavy chain (HC) and light chain (LC) colored in blue and green, respectively.

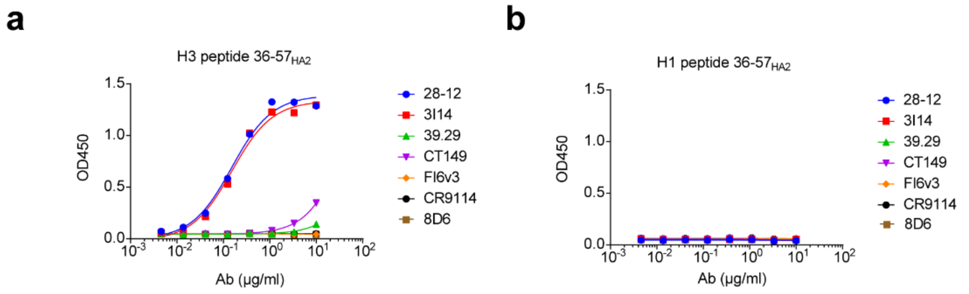

Supplementary Figure 10. The binding pattern of helix A-targeting bnAbs to the peptides 36-57$_{HA2}$ of H1 and H3. a-b, The reactivity of multiple reported stem bnAbs to peptide 36-57$_{HA2}$ of H3N2 **(a)** and H1N1 **(b)**. Data are presented as mean values of duplicates. Representative data are shown from two independent experiments (**a-b**).

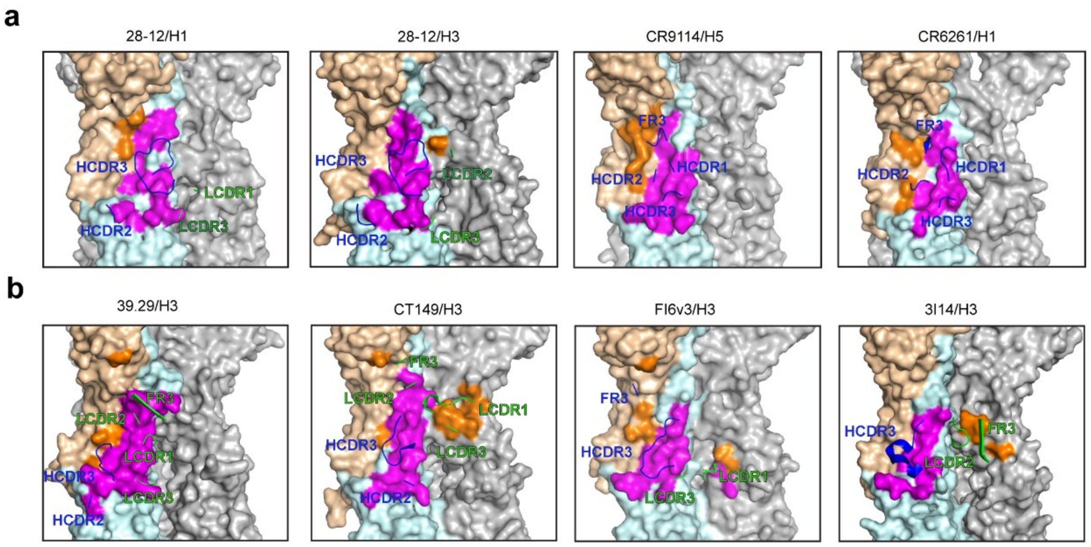

Supplementary Figure 11. Interactions between 28-12 and H3/H1 HAs and comparison with other helix A bounding antibodies. The epitope residues in HA1 are colored in orange and those in HA2 are colored in magenta, with the heavy chains in blue and light chains in green.

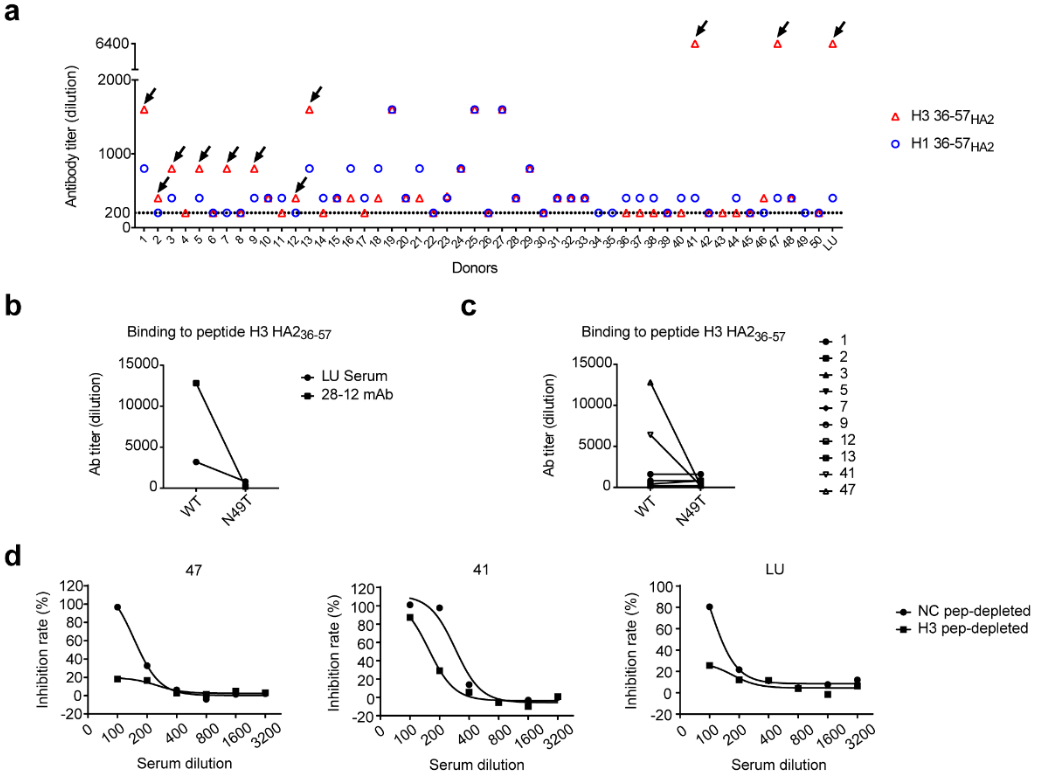

Supplementary Figure 12. Frequency of 28-12 epitope-targeting serum antibodies in vaccinated donors. a, ELISA-based binding activity of sera from 50 donors vaccinated with seasonal trivalent inactivated vaccine in 2020 to peptides 36-57$_{HA2}$ of H3N2 and H1N1, showing as antibody endpoint titer. 10 sera showed higher binding activity to the peptide 36-57$_{HA2}$ of H3N2 than H1N1, which is marked with black arrows. Serum LU, the source of 28-12, was the positive control. **b,** ELISA-based binding activity of antibody 28-12 and serum LU to H3N2 peptide 36-57$_{HA2}$ with N49T mutation as compared to WT peptide, showing as antibody endpoint titer. **c,** ELISA-based binding activity of 10 sera to H3N2 peptide 36-57$_{HA2}$ with N49T mutation as compared to WT peptide. **d,** Neutralization curves of H3 peptide-depleted sera against H3N2 virus as compared with the unrelated NC (negative control) peptide-depleted sera. Figure is representative of two independent experiments. Data represent as mean values of duplicates.

Supplementary Table 1. Statistics of cryo-EM data collection, processing, and model validation

	28-12-H3	28-12-H1
Data collection		
EM equipment	FEI Titan Krios	
Voltage (kV)	300	
Detector	K2	
Pixel size (Å)	1.318	
Electron dose ($e^-/Å^2$)	38	
Exposure time (s)	7.6	
Frames	38	
Defocus range (μm)	-1.0~-3.0	
Reconstruction		
Software	Relion 3.1& Cryosparc	
Final particles	105,383	124,947
Symmetry	C3	C3
Final overall resolution (Å)	3.7	3.5
Map-sharpening B factor ($Å^2$)	-122.8	-119.5
Refinement		
Software	Phenix& Rosetta& Coot	
Rms deviations		
Bond length (Å)	0.0035	0.0036
Bond angle (°)	0.92	0.96
Ramachandran plot statistics (%)		
Favored	98.11	97.97
Allowed	1.89	2.03
Outlier	0.00	0.00

Supplementary Table 2. Hydrophobic interaction between 28-12 Fab and H3 and H1 HAs

	Subunit	Domain	HA[a]	28-12[b]
H3	HA2	Helix A	Ala 36	Ile 58, Tyr 112
			Leu 38	Tyr 59, Tyr 112, Tyr 113, Phe 114, Phe 94 (Vκ)
			Ile 45	Val 109, Val 110, Tyr 112
			Ile 48	Val 109
			Leu 52	Tyr 108, Val 109
			Ile 56	Tyr 108
		FP	Trp 21	Val 109
H1	HA2	Helix A	Ala 36	Ile 58, Tyr 112
			Leu 38	Tyr 59, Tyr 112, Tyr 113, Phe 114, Phe 94 (Vκ)
			Ile 45	Val 109, Val 110, Tyr 112
			Ile 48	Val 109
			Val 52	Tyr 108, Val 109
			Ile 56	Tyr 108
		FP	Trp 21	Val 109
	HA1		Val 40	Tyr 108, Val 109
			Leu 300	Tyr 108

a, All the amino acids are shown according H3N2 HA numbering. FP, fusion peptide.

b, All the amino acids shown represent the heavy chain of 28-12 except for Phe 94, which is specifically annotated as the light chain.

Supplementary Table 3. Hydrogen bonding between 28-12 Fab and H3 or H1 HAs.

	Subunit	Domain	HA[a]	28-12[b]	
H3	HA2	FP	Asp 19	Thr 57, Tyr 112	V_H
		Helix A	Gln 42	Gly 101, Tyr 112	
			Asp 46	Tyr 115, Ser 103	
			Asn 49	Cys 102, Asn 106, Tyr 108, Val 109	
			Asn 53	Ser 104, Asn 106	
			Glu 57	Asn 106	
			Lys 39	Asp 92 (Salt bridge)	V_K
H1	HA2	FP	Asp 19	Ser 54, Asn 56, Thr 57, Tyr 112	V_H
		Helix A	Gln 42	Gly 101, Tyr 112	
			Asp 46	Tyr 115, Ser 103	
			Asn 53	Asn 106	
			Glu 57	Thr 105	
			Lys 39	Asp 92 (Salt bridge)	V_K
	HA1		Ser 299	Tyr 108	V_H

a, All the amino acids are shown according H3N2 HA numbering. FP, fusion peptide.

b, All the amino acids shown represent the heavy chain or light chain of 28-12.

Supplementary Table 4. Atom-to-atom contacts between 28-12 Fab and H3 HA

	H3 HA	28-12	Contacts[a]	
VH	I18	S54	3	HA2: fusion peptide (17)[b]
	D19	S54, T57, Y112	3, 3, 4	
	G20	Y112	2	
	W21	V109	2	
	A36	Y112	1	HA2: helix A (182)
	L38	Y59, P114, Y113, Y112	5, 9, 1, 4	
	K39	P114	2	
	T41	Y112	4	
	Q42	Y115, P114, Y112, G111, G101, C102	5, 8, 14, 5, 4, 2	
	I45	G111, V109, V110	2, 4, 1	
	D46	Y115, S103, C102	12, 7, 3	
	N49	V109, Y108, C107, N106, S104, C102	6, 7, 7, 4, 3, 5	
	L52	Y108, V109	1, 1	
	N53	N106, T105, S104, C107, Y108	10, 4, 8, 1, 6	
	I56	Y108	10	
	E57	T105, N106, Y108	2, 10, 4	
VK	K39	D92, S93	5, 4	HA2: helix A (9)

a. Numbers represent the number of atom-t-atom contacts between the antibody residues and the HA residues (the distance cut-off is 4.5 angstrom).
b. Numbers in parentheses represent the total number of atom-to-atom contacts.

Supplementary Table 5. Atom-to-atom contacts between 28-12 Fab and H1 HA

	H1 HA	28-12	Contacts[a]	
V_H	V40	Y108, V109	6, 1	HA1 (20)[b]
	S299	Y108	9	
	L300	Y108	1	
	T326	V109	3	
	V18	S54, N56	2, 1	HA2: fusion peptide (28)
	D19	S52, S54, N56, T57, Y112	2, 4, 8, 1, 7	
	G20	Y112	1	
	W21	V109	2	
	A36	Y112	1	HA2: helix A (136)
	L38	Y59, P114, Y112, Y113	1, 10, 7, 1	
	K39	P114	4	
	Q42	G101, C102, Y115, P114, Y112, G111	4, 1, 4, 9, 9, 6	
	N43	Y32	1	
	I45	G111, V110, V109	2, 2, 5	
	D46	Y115, S103, C102	9, 7, 3	
	T49	C102, S104, N106, C107, Y108, V109	2, 3, 1,1, 1, 2	
	N50	S103, S104	1, 4	
	V52	Y108	4	
	N53	N106, S104, T105, Y108	11, 6, 1, 3	
	I56	Y108	3	
	E57	T105	7	
V_K	K39	Y32, D92	1,7	HA2: helix A (8)

a. Numbers represent the number of atom-to-atom contacts between the antibody residues and the HA residues (the distance cutoff is 4.5 angstrom).

b. Numbers in parentheses represent the total number of atom-to-atom contacts.
