# Supplementary material for: Unique binding pattern for a lineage of human antibodies with broad reactivity against influenza A virus
Source: Nat Commun. 2022 May 2;13:2378. doi: 10.1038/s41467-022-29950-w (PMC9061721; doi:10.1038/s41467-022-29950-w)
Supplement: Supplementary file 3 — Reporting Summary [file 41467_2022_29950_MOESM3_ESM.pdf]

nature research

Corresponding author(s): Bing Sun

Last updated by author(s): Mar 29, 2022

Reporting Summary

Nature Research wishes to improve the reproducibility of the work that we publish. This form provides structure for consistency and transparency in reporting. For further information on Nature Research policies, see our Editorial Policies and the Editorial Policy Checklist.

Statistics

For all statistical analyses, confirm that the following items are present in the figure legend, table legend, main text, or Methods section.

n/a	Confirmed	
☐	☒	The exact sample size (n) for each experimental group/condition, given as a discrete number and unit of measurement
☐	☒	A statement on whether measurements were taken from distinct samples or whether the same sample was measured repeatedly
☒	☐	The statistical test(s) used AND whether they are one- or two-sided<br>*Only common tests should be described solely by name; describe more complex techniques in the Methods section.*
☒	☐	A description of all covariates tested
☒	☐	A description of any assumptions or corrections, such as tests of normality and adjustment for multiple comparisons
☐	☒	A full description of the statistical parameters including central tendency (e.g. means) or other basic estimates (e.g. regression coefficient) AND variation (e.g. standard deviation) or associated estimates of uncertainty (e.g. confidence intervals)
☒	☐	For null hypothesis testing, the test statistic (e.g. F, t, r) with confidence intervals, effect sizes, degrees of freedom and P value noted<br>*Give P values as exact values whenever suitable.*
☒	☐	For Bayesian analysis, information on the choice of priors and Markov chain Monte Carlo settings
☒	☐	For hierarchical and complex designs, identification of the appropriate level for tests and full reporting of outcomes
☒	☐	Estimates of effect sizes (e.g. Cohen's d, Pearson's r), indicating how they were calculated

Our web collection on statistics for biologists contains articles on many of the points above.

Software and code

Policy information about availability of computer code

Data collection	Olympus cellSens Entry, BD Influx Cell Sorter, Bio-Tek Epoch 2, OCTET DATA acquisition 9.0, FEI TEM user interface 2.15.3, SerialEM 3.7.0, GE healthcare ImageQuant LAS4000 mini biomolecular imager
Data analysis	Graphpad Prism 7.0, Flowlo V10, Fortebio Data Analysis 9.0, MotionCor2, goCTF, Relion 3.1, CryoSPARC v2.15.0, CTFFIND 4.1.8, Phenix 1.12-2829, Rosetta 2017, COOT 0.8.7, UCSF Chimera 1.10.2, UCSF ChimeraX 1.0, PDBePISA 1.48.

For manuscripts utilizing custom algorithms or software that are central to the research but not yet described in published literature, software must be made available to editors and reviewers. We strongly encourage code deposition in a community repository (e.g. GitHub). See the Nature Research guidelines for submitting code & software for further information.

Data

Policy information about availability of data

All manuscripts must include a data availability statement. This statement should provide the following information, where applicable:

- Accession codes, unique identifiers, or web links for publicly available datasets
- A list of figures that have associated raw data
- A description of any restrictions on data availability

All data presented in this study are available within the figures and in the Supplementary Information. Source data are provided with this paper. Cryo-EM maps for the 28-12-H3 complex and 28-12-H1 complex have been deposited at the Electron Microscopy Data Bank with accession codes EMD-33023 [https://www.ebi.ac.uk/pdbe/entry/emdb/EMD-33023], and EMD-33024 [https://www.ebi.ac.uk/pdbe/entry/emdb/EMD-33024], respectively. Associated atomic models have been deposited in the Protein Data Bank with accession codes 7X6L [https://doi.org/10.2210/pdb7X6L/pdb] and 7X6O [https://doi.org/10.2210/pdb7X6O/pdb] for 28-12-H3 and 28-12-H1, respectively. The sequences used in Figure 5 for analysis were: H3 A/HongKong/01/1968 (UniProtKB/Swiss-Prot: Q91MA7.1,https://www.ncbi.nlm.nih.gov/protein/Q91MA7/), H7 A/Netherlands/219/2003 (GenBank: AAR02640.2, https://www.ncbi.nlm.nih.gov/protein/AAR02640), H4 A/swine-

ontario/01911-1/1999 (GenBank: AAG17429.1, https://www.ncbi.nlm.nih.gov/protein/AAG17429.1), H14 A/Mallard/Astrakhan/263/1982 (UniProtKB/Swiss-Prot: P26136.1, https://www.ncbi.nlm.nih.gov/protein/P26136.1), H15 A/duck/AUS/341/1983 (GenBank: ABB88132.1, https://www.ncbi.nlm.nih.gov/protein/ABB88132.1), H1 A/California/06/2009 (GenBank: ACP41105.1, https://www.ncbi.nlm.nih.gov/protein/ACP41105.1) and H10 A/Jiangxi-Donghu/346/2013 (GISAID Accession:EPI497477, https://platform.epicov.org/epi3/frontend#412409).

Field-specific reporting

Please select the one below that is the best fit for your research. If you are not sure, read the appropriate sections before making your selection.

☒ Life sciences ☐ Behavioural & social sciences ☐ Ecological, evolutionary & environmental sciences

For a reference copy of the document with all sections, see nature.com/documents/nr-reporting-summary-flat.pdf

Life sciences study design

All studies must disclose on these points even when the disclosure is negative.

Sample size	For animal experiment, each group included five mice. All the mice used were 7 week-old female mice. The number of mice in each group meet the requirement for statistical analysis, which is sufficient given the technical reproducibility.
Data exclusions	No data was excluded.
Replication	Animals were randomly divided into experimental groups. All experiments were performed at least two times as independent experiments This is indicated in the figure legends for each experiments.
Randomization	Animals were randomly divided into experimental groups.
Blinding	The investigators were not blinded to allocation during experiments and outcome assessment. Data collection and analysis were performed by different people. Blinding was not relevant as the reported data were not based on subjective observations, but quantitative measurements, including BLI, ELISA, weight loss and survival etc.

Behavioural & social sciences study design

All studies must disclose on these points even when the disclosure is negative.

Study description	*Briefly describe the study type including whether data are quantitative, qualitative, or mixed-methods (e.g. qualitative cross-sectional, quantitative experimental, mixed-methods case study).*
Research sample	*State the research sample (e.g. Harvard university undergraduates, villagers in rural India) and provide relevant demographic information (e.g. age, sex) and indicate whether the sample is representative. Provide a rationale for the study sample chosen. For studies involving existing datasets, please describe the dataset and source.*
Sampling strategy	*Describe the sampling procedure (e.g. random, snowball, stratified, convenience). Describe the statistical methods that were used to predetermine sample size OR if no sample-size calculation was performed, describe how sample sizes were chosen and provide a rationale for why these sample sizes are sufficient. For qualitative data, please indicate whether data saturation was considered, and what criteria were used to decide that no further sampling was needed.*
Data collection	*Provide details about the data collection procedure, including the instruments or devices used to record the data (e.g. pen and paper, computer, eye tracker, video or audio equipment) whether anyone was present besides the participant(s) and the researcher, and whether the researcher was blind to experimental condition and/or the study hypothesis during data collection.*
Timing	*Indicate the start and stop dates of data collection. If there is a gap between collection periods, state the dates for each sample cohort.*
Data exclusions	*If no data were excluded from the analyses, state so OR if data were excluded, provide the exact number of exclusions and the rationale behind them, indicating whether exclusion criteria were pre-established.*
Non-participation	*State how many participants dropped out/declined participation and the reason(s) given OR provide response rate OR state that no participants dropped out/declined participation.*
Randomization	*If participants were not allocated into experimental groups, state so OR describe how participants were allocated to groups, and if allocation was not random, describe how covariates were controlled.*

Ecological, evolutionary & environmental sciences study design

All studies must disclose on these points even when the disclosure is negative.

Study description	*Briefly describe the study. For quantitative data include treatment factors and interactions, design structure (e.g. factorial, nested, hierarchical), nature and number of experimental units and replicates.*
Research sample	*Describe the research sample (e.g. a group of tagged Passer domesticus, all Stenocereus thurberi within Organ Pipe Cactus National Monument), and provide a rationale for the sample choice. When relevant, describe the organism taxa, source, sex, age range and any manipulations. State what population the sample is meant to represent when applicable. For studies involving existing datasets, describe the data and its source.*
Sampling strategy	*Note the sampling procedure. Describe the statistical methods that were used to predetermine sample size OR if no sample-size calculation was performed, describe how sample sizes were chosen and provide a rationale for why these sample sizes are sufficient.*
Data collection	*Describe the data collection procedure, including who recorded the data and how.*
Timing and spatial scale	*Indicate the start and stop dates of data collection, noting the frequency and periodicity of sampling and providing a rationale for these choices. If there is a gap between collection periods, state the dates for each sample cohort. Specify the spatial scale from which the data are taken*
Data exclusions	*If no data were excluded from the analyses, state so OR if data were excluded, describe the exclusions and the rationale behind them, indicating whether exclusion criteria were pre-established.*
Reproducibility	*Describe the measures taken to verify the reproducibility of experimental findings. For each experiment, note whether any attempts to repeat the experiment failed OR state that all attempts to repeat the experiment were successful.*
Randomization	*Describe how samples/organisms/participants were allocated into groups. If allocation was not random, describe how covariates were controlled. If this is not relevant to your study, explain why.*
Blinding	*Describe the extent of blinding used during data acquisition and analysis. If blinding was not possible, describe why OR explain why blinding was not relevant to your study.*

Did the study involve field work? ☐ Yes ☐ No

Field work, collection and transport

Field conditions	*Describe the study conditions for field work, providing relevant parameters (e.g. temperature, rainfall).*
Location	*State the location of the sampling or experiment, providing relevant parameters (e.g. latitude and longitude, elevation, water depth).*
Access & import/export	*Describe the efforts you have made to access habitats and to collect and import/export your samples in a responsible manner and in compliance with local, national and international laws, noting any permits that were obtained (give the name of the issuing authority, the date of issue, and any identifying information).*
Disturbance	*Describe any disturbance caused by the study and how it was minimized.*

Reporting for specific materials, systems and methods

We require information from authors about some types of materials, experimental systems and methods used in many studies. Here, indicate whether each material, system or method listed is relevant to your study. If you are not sure if a list item applies to your research, read the appropriate section before selecting a response.

Materials & experimental systems

n/a	Involved in the study	
☐	☒	Antibodies
☐	☒	Eukaryotic cell lines
☒	☐	Palaeontology and archaeology
☐	☒	Animals and other organisms
☐	☒	Human research participants
☒	☐	Clinical data
☒	☐	Dual use research of concern

Methods

n/a	Involved in the study	
☒	☐	ChIP-seq
☐	☒	Flow cytometry
☒	☐	MRI-based neuroimaging

Antibodies

Antibodies used	Influenza virus HA specific antibodies 28-12, MEDI8852, 39.29, CR9114, CR8020, CT149, 3I14 and FI6v3 were prepared in our lab. The isotype control antibody 8D6 was also prepared in our lab.<br>APC mouse anti-human IgG (Cat: 550931, Clone G18-145 (RUO), BD Biosciences, 20ul/test),<br>Cy3-Streptavidin (Product Number: S6402, Sigma-Aldrich, 1:100 dilution),<br>Mouse anti-6*His-HRP monoclonal antibody (HRP-66005,Proteintech, 1:10000 dilution),<br>Anti-Influenza NP polycolonal antibody was prepared in our lab previously (1:8000 dilution).<br>Goat anti-human IgG Fab specific antibody (Cat: I5260, Sigma-Aldrich, for ELISA coating: 5 μg/ml),<br>FITC mouse anti-human CD19 (Cat: 555412, Clone HIB19 (RUO), BD Biosciences, 20ul/test),<br>Anti-human IgG Fc-HRP (Cat: A0170, Sigma-Aldrich, 1:8000 dilution), Streptavidin-HRP (Cat: DY998, R&D systems, 1:200 dilution)
Validation	The validation of commercially available antibodies used in this study was described in technical data sheets provided by the manufacturers and/or on their websites. Antibody 28-12 was validated in this study. Antibodies MEDI8852 (Nicole L et al. Cell. 2016), 39.29 (Gerald Nakamura et al.Cell Host & Microbe. 2013), CR9114 (Cyrille Dreyfus et al. Science, 2012), CR8020 (Damian C. Ekiert et al. Science. 2011), CT149 (Ying Wu et al. Nature Communications. 2015), 3I14 (Ying Fu et al. Nature Communications. 2016) and FI6v3 (Davide Corti et al. Science. 2011) have described previously and also validated in this study. The isotype control antibody 8D6 was described previously (Chunyan Yi et al. Cellular& Molecular Immunology. 2020). Anti-Influenza NP polycolonal antibody has been used previouly in our study (Wenshuai Wang et al.Nature Communiations. 2016).

Eukaryotic cell lines

Policy information about cell lines

Cell line source(s)	HEK 293T cell, ATCC<br>MDCK cell,ATCC<br>ExpiCHO cell, Thermo Fisher Scientific<br>HeLa cell, ATCC<br>HEp-2 cell, ATCC
Authentication	All cell lines were frequently checked and used for cellular morphologies, growth rates and functions in our lab and were not commonly misidentified.
Mycoplasma contamination	The cell lines were not tested for Mycoplasma after purchase.
Commonly misidentified lines (See ICLAC register)	No commonly misidentified cell lines were used.

Palaeontology and Archaeology

Specimen provenance	*Provide provenance information for specimens and describe permits that were obtained for the work (including the name of the issuing authority, the date of issue, and any identifying information).*
Specimen deposition	*Indicate where the specimens have been deposited to permit free access by other researchers.*
Dating methods	*If new dates are provided, describe how they were obtained (e.g. collection, storage, sample pretreatment and measurement), where they were obtained (i.e. lab name), the calibration program and the protocol for quality assurance OR state that no new dates are provided.*

☐ Tick this box to confirm that the raw and calibrated dates are available in the paper or in Supplementary Information.

Ethics oversight	*Identify the organization(s) that approved or provided guidance on the study protocol, OR state that no ethical approval or guidance was required and explain why not.*

Note that full information on the approval of the study protocol must also be provided in the manuscript.

Animals and other organisms

Policy information about studies involving animals; ARRIVE guidelines recommended for reporting animal research

Laboratory animals	Wide-type 7 week-old BALB/c female mice were purchased from Shanghai Lingchang Biotechnology Co., Ltd and used to test the protective efficacy of 28-12. The mice were kept in SPF (specific pathogen free) facilities of BSL-2 lab with controlled temperature (20-26°C), humidity (40-70%) and lighting conditions (12h light/ 12h dark cycles).
Wild animals	The study did not involve wild animals.
Field-collected samples	The study did not involve samples collected from the field.
Ethics oversight	The animal studies were approved by the Institutional Animal Care and Use Committee at Institut Pasteur of Shanghai, China.

Note that full information on the approval of the study protocol must also be provided in the manuscript.

Human research participants

Policy information about studies involving human research participants

Population characteristics	For the memory B cell isolation, one female healthy donor aged about 26 in china was selected. After vaccination with a trivalent seasonal split vaccine in 2016, we collected the blood sample of the donor for HA specific memory B cells isolation. For the study of frequency of 28-12 epitope-targeting serum antibodies in human population, 50 healthy donors aged from 3 to 86 were vaccinated with the trivalent seasonal split vaccine in 2020. The blood sample were collected for binding and neutralization assays.
Recruitment	Study participants were recruited on the random basis from the healthy cases. There was no potential self-selection bias or other biases during the selection. All the donors agreed to provide the biospecimen for detection, further diagnostic and scientific research.
Ethics oversight	For the memory B cell isolation, the study was approved by the Ethics Review Committee of Institut Pasteur of Shanghai, Chinese Academic of Sciences. For the collection and study of the 50 vaccinated donors' sera were approved by the Ethical Review Committee of National Institute for Viral Disease Control and Prevention, China CDC.

Note that full information on the approval of the study protocol must also be provided in the manuscript.

Clinical data

Policy information about clinical studies

All manuscripts should comply with the ICMJEguidelines for publication of clinical research and a completedCONSORT checklist must be included with all submissions.

Clinical trial registration	*Provide the trial registration number from ClinicalTrials.gov or an equivalent agency.*
Study protocol	*Note where the full trial protocol can be accessed OR if not available, explain why.*
Data collection	*Describe the settings and locales of data collection, noting the time periods of recruitment and data collection.*
Outcomes	*Describe how you pre-defined primary and secondary outcome measures and how you assessed these measures.*

Dual use research of concern

Policy information about dual use research of concern

Hazards

Could the accidental, deliberate or reckless misuse of agents or technologies generated in the work, or the application of information presented in the manuscript, pose a threat to:

No	Yes	
☐	☐	Public health
☐	☐	National security
☐	☐	Crops and/or livestock
☐	☐	Ecosystems
☐	☐	Any other significant area

Experiments of concern

Does the work involve any of these experiments of concern:

No	Yes	
☐	☐	Demonstrate how to render a vaccine ineffective
☐	☐	Confer resistance to therapeutically useful antibiotics or antiviral agents
☐	☐	Enhance the virulence of a pathogen or render a nonpathogen virulent
☐	☐	Increase transmissibility of a pathogen
☐	☐	Alter the host range of a pathogen
☐	☐	Enable evasion of diagnostic/detection modalities
☐	☐	Enable the weaponization of a biological agent or toxin
☐	☐	Any other potentially harmful combination of experiments and agents

ChIP-seq

Data deposition

- [ ] Confirm that both raw and final processed data have been deposited in a public database such as GEO.
- [ ] Confirm that you have deposited or provided access to graph files (e.g. BED files) for the called peaks.

Data access links<br>*May remain private before publication.*	*For "Initial submission" or "Revised version" documents, provide reviewer access links. For your "Final submission" document, provide a link to the deposited data.*
Files in database submission	*Provide a list of all files available in the database submission.*
Genome browser session<br>(e.g. UCSC)	*Provide a link to an anonymized genome browser session for "Initial submission" and "Revised version" documents only, to enable peer review. Write "no longer applicable" for "Final submission" documents.*

Methodology

Replicates	*Describe the experimental replicates, specifying number, type and replicate agreement.*
Sequencing depth	*Describe the sequencing depth for each experiment, providing the total number of reads, uniquely mapped reads, length of reads and whether they were paired- or single-end.*
Antibodies	*Describe the antibodies used for the ChIP-seq experiments; as applicable, provide supplier name, catalog number, clone name, and lot number.*
Peak calling parameters	*Specify the command line program and parameters used for read mapping and peak calling, including the ChIP, control and index files used.*
Data quality	*Describe the methods used to ensure data quality in full detail, including how many peaks are at FDR 5% and above 5-fold enrichment.*
Software	*Describe the software used to collect and analyze the ChIP-seq data. For custom code that has been deposited into a community repository, provide accession details.*

Flow Cytometry

Plots

Confirm that:

- [ ] The axis labels state the marker and fluorochrome used (e.g. CD4-FITC).
- [ ] The axis scales are clearly visible. Include numbers along axes only for bottom left plot of group (a 'group' is an analysis of identical markers).
- [ ] All plots are contour plots with outliers or pseudocolor plots.
- [ ] A numerical value for number of cells or percentage (with statistics) is provided.

Methodology

Sample preparation	PBMCs were isolated from the collected blood by using the Ficoll-Paque gradient (GE Healthcare, cat. no. 17144002). The PBMCs were stained with a FITC-labelled mouse anti-human CD19 antibody (BD PharmingenTM, cat. no. 555412) and an APC-labelled mouse anti-human IgG antibody (BD PharmingenTM, cat. no. 550931). The recombinant HK/68 H3N2 HA protein was labelled with biotin using the EZ-Link Sμlfo-NHS-LC Biotin biotinylation reagent (Thermo Fisher Scientific, cat. no. 21335), which could bind to the streptavidin-Cy3 conjugate (Sigma-Aldrich, cat. no. S6402). Single FITC-CD19+/APC-IgG+/ streptavidin-Cy3 conjugate-HA-specific memory B cells were isolated with a BD Influx Cell Sorter and sorted into 96-well plates.
Instrument	BD Influx Cell Sorter
Software	FlowJo v10 software
Cell population abundance	No post-sort cell fraction were used for further analyze.
Gating strategy	H3N2 HA-specific B cells were gated as CD19+IgG+H3N2 HA+ . More information are available in Methods section.

- [x] Tick this box to confirm that a figure exemplifying the gating strategy is provided in the Supplementary Information.

Magnetic resonance imaging

Experimental design

Design type	*Indicate task or resting state; event-related or block design.*
Design specifications	*Specify the number of blocks, trials or experimental units per session and/or subject, and specify the length of each trial or block (if trials are blocked) and interval between trials.*
Behavioral performance measures	*State number and/or type of variables recorded (e.g. correct button press, response time) and what statistics were used to establish that the subjects were performing the task as expected (e.g. mean, range, and/or standard deviation across subjects).*

Acquisition

Imaging type(s)	*Specify: functional, structural, diffusion, perfusion.*
Field strength	*Specify in Tesla*
Sequence & imaging parameters	*Specify the pulse sequence type (gradient echo, spin echo, etc.), imaging type (EPI, spiral, etc.), field of view, matrix size, slice thickness, orientation and TE/TR/flip angle.*
Area of acquisition	*State whether a whole brain scan was used OR define the area of acquisition, describing how the region was determined.*

Diffusion MRI ☐ Used ☐ Not used

Preprocessing

Preprocessing software	*Provide detail on software version and revision number and on specific parameters (model/functions, brain extraction, segmentation, smoothing kernel size, etc.).*
Normalization	*If data were normalized/standardized, describe the approach(es): specify linear or non-linear and define image types used for transformation OR indicate that data were not normalized and explain rationale for lack of normalization.*
Normalization template	*Describe the template used for normalization/transformation, specifying subject space or group standardized space (e.g. original Talairach, MNI305, ICBM152) OR indicate that the data were not normalized.*
Noise and artifact removal	*Describe your procedure(s) for artifact and structured noise removal, specifying motion parameters, tissue signals and physiological signals (heart rate, respiration).*
Volume censoring	*Define your software and/or method and criteria for volume censoring, and state the extent of such censoring.*

Statistical modeling & inference

Model type and settings	*Specify type (mass univariate, multivariate, RSA, predictive, etc.) and describe essential details of the model at the first and second levels (e.g. fixed, random or mixed effects; drift or auto-correlation).*
Effect(s) tested	*Define precise effect in terms of the task or stimulus conditions instead of psychological concepts and indicate whether ANOVA or factorial designs were used.*

Specify type of analysis: ☐ Whole brain ☐ ROI-based ☐ Both

Statistic type for inference (See Eklund et al. 2016)	*Specify voxel-wise or cluster-wise and report all relevant parameters for cluster-wise methods.*
Correction	*Describe the type of correction and how it is obtained for multiple comparisons (e.g. FWE, FDR, permutation or Monte Carlo).*

Models & analysis

n/a	Involved in the study
☐	☐ Functional and/or effective connectivity
☐	☐ Graph analysis
☐	☐ Multivariate modeling or predictive analysis

Functional and/or effective connectivity	*Report the measures of dependence used and the model details (e.g. Pearson correlation, partial correlation, mutual information).*
Graph analysis	*Report the dependent variable and connectivity measure, specifying weighted graph or binarized graph, subject- or group-level, and the global and/or node summaries used (e.g. clustering coefficient, efficiency, etc.).*

Multivariate modeling and predictive analysis

Specify independent variables, features extraction and dimension reduction, model, training and evaluation metrics.
