# Supplementary material for: Unique binding pattern for a lineage of human antibodies with broad reactivity against influenza A virus
Source: Nat Commun. 2022 May 2;13:2378. doi: 10.1038/s41467-022-29950-w (PMC9061721; doi:10.1038/s41467-022-29950-w)
Supplement: Supplementary file 2 — Peer Review File [file 41467_2022_29950_MOESM2_ESM.pdf]

nature portfolio

Peer Review File

Unique binding pattern for a lineage of human antibodies with broad reactivity against influenza A virus

Open Access This file is licensed under a Creative Commons Attribution 4.0 International License, which permits use, sharing, adaptation, distribution and reproduction in any medium or format, as long as you give appropriate credit to the original author(s) and the source, provide a link to the Creative Commons license, and indicate if changes were made. In the cases where the authors are anonymous, such as is the case for the reports of anonymous peer reviewers, author attribution should be to 'Anonymous Referee' followed by a clear attribution to the source work. The images or other third party material in this file are included in the article's Creative Commons license, unless indicated otherwise in a credit line to the material. If material is not included in the article's Creative Commons license and your intended use is not permitted by statutory regulation or exceeds the permitted use, you will need to obtain permission directly from the copyright holder. To view a copy of this license, visit http://creativecommons.org/licenses/by/4.0/.

REVIEWER COMMENTS, first round

Reviewer #1 (Remarks to the Author):

In this study Sun et al. present a lineage of broadly neutralizing antibodies (Bnabs) that neutralize group 1 and 2 strains. Amongst four members of the lineage that shared the same germ-line VH3-48/VK1-12,Bnab 28-12 was the most potent and further characterized. Employing biochemical and structural analyses, the authors discovered that 28-12 has distinct binding modes for H3 (group 2) and H1 (group 1) viruses, binding a continuos epitope within H3 but requiring conformational binding for H1. Analysis of germline-reverted Ab and intermediate mutants suggests that 28-12 was initially selected against an H3 strain and then evolved to have H1 binding ability. Overall, this is an intriguing result, but there are several aspects that deserve further attention.

Specific points:
A comprehensive comparison to other bnAbs that interact with Helix A would be important to include throughout the mansucript. As the authors particularly underline that their mAb has a long CDRH3 (23aa) they should include bnAb 3I14 in this comparison as this mAb appears to have similar features, including a 23aa CDRH3.

The last part of the introduction contains a very long summary of the results, which are again summarized in the discussion. The actual discussion part is however relatively short. A more in depth discussion how 28-12 compares to existing bnAbs would be important.

To ease the reader, the full IAV strains tested should be introduced on first mentioning and noted which strains are human infecting and which not.

If I understand the presented data correctly, 19 antibodies from this donor were successfully sorted and cloned using. Four of these belonged to the same lineage. This seems a very high proportion. Was the sample collected shortly after vaccination? The authors should try to quantify the contribution of this lineage further eg by bulk heavy chain NGS of HA bait sorted cells.

Fig1: The methods and legend do not state which HA as was used as bait. Strain/Group needs to be added.

There is very little information on the donor provided: "Blood was collected from a female volunteer previously inoculated with seasonal split influenza vaccine after she had signed the informed consent form." The authors need to specific the precise vaccine (manufacturer, year, which precise HA antigens were included). Time between vaccination and sampling should be stated. Was this the first (ever) vaccination that the person received? History of vaccination and prior IAV infection should be listed. How was the neutralization capacity of the donor plasma?

Line 115-117: The authors state: "The results showed that 28-2, 28-4, 28-6, and 28-12 exhibited cross-neutralizing activity against H1N1 and H3N2, with IC50 values ranging from 0.6382 μg/ml to 7.358 μg/ml (Fig. 1d)." however, according to figure 1d, Abs 28-8, 28-15, 28-16, 28-19, 28-27, and 28-28 also show cross-neutralization. Why where these not followed up further? So in all only 5 of the 19 analyzed Abs did not show cross neutralization activity. This is quite astonishing that so many of the antibodies are neutralizing. The authors should discuss this and also include published bnAbs as reference in their analysis

Figure 1d: the full neutralization data and IC50 should be shown.

To underline the claim that 28-12 recognizes a novel epitope the authors should conduct competition mapping and/ or docking analyses with other bnAbs.

Line 122/123 "…indicating a positive correlation between this lineage and neutralizing activity". This needs to be rephrased, no test for correlation has been performed

Line 135 "significant" cannot be used in this context as no statistical test was performed.

Line 167 Therapeutic dose needs to be stated in the text

Line 209 Typo: "In contrast" instead of "In contract"

Line 268 The subtitle needs to be shortened and rephrased. "was expected" does not fit in this context.

Lines 309-312: "28-2, 28-4, 28-6 and 28-12 are unique, as they belong to the same lineage VH3-48/DH2-2/JH6 and VK1-12/JK5. The usage of germline gene VH3-48, together with previously reported influenza bnAb germline genes, indicates the diverse germline selection of broad-spectrum antibodies against IAVs." This sentence needs to be rephrased to make sense.

Reviewer #2 (Remarks to the Author):

In this study, Sun, Liu, Lu and colleagues, describe a novel class of neutralizing antibodies
The authors use binding, neutralization and structural determination experiments to show that these Abs are relatively broad in their binding breadth, that they neutralize H1N! and H3N2 viruses and that they bind the HA stem epitope. Additionally, these antibodies utilize VH3-48/VK1-12 variable immunoglobulin genes – a hitherto undescribed combination for HA stem binding antibodies. Particularly interesting is the finding that a member of this lineage cross-reacts with an H1 and an H3 HA and that its binding mode is slightly different on a different subtype, as it engages a single alpha-helix on an H3, but it requires a conformational epitope consisting of HA1 and HA2 subdomains.

While the structural information provided is very useful as it compares the binding to both an H1 (group 1 influenzas) and an H3 (group 2), the study is limited to a single donor and it is unclear how prevalent this class of antibodies is in the general population. The donor's exposure history is not reported so it is impossible to appreciate whether these antibodies were generated under uncommon antigenic exposure conditions. Additionally, the authors call these antibodies broadly neutralizing, but the panel of viruses/HAs is quite limited. Indeed, the authors show that the Abs bind to heterosubtypic HAs, but binding to historical isolates needs to be demonstrated for the Abs to be defined as broadly neutralizing.

Major concerns:

1- Lines 129-130 – "among which 28-4, 28-12 exhibited stronger binding activity than MEDI88529 and 39.29" – binding signal in ELISA does not equate to "stronger binding". For example, MEDI8852 saturates at lower concentration than 28-12. The authors should provide comparative Kd values, preferably from kinetic measurements.
2- Do you know the exposure history of this individual? This would help corroborate the idea that this lineage was initiated with an H3N2 and affinity matured against an H1N1. In any event, a binding experiment against a panel of historical H3 and H1 HAs with both the mature antibody and the germline-reverted one needs to be performed.
3- Were these antibodies tested for auto/polyreactivity? Stem mAbs are typically prone to polyreactivity (see for ex. PMID: 26631631 and PMID: 30837606) and this needs to be tested.

Minor comments:

1 - Figure 1 – legend is inverted – should say group 1 SC/09 H1N1 and group 2 HK/68 H3N2

2 - Line 119-120 "Unexpectedly, a large portion of antibodies utilized the VH3-48 germline genes" Why was it unexpected? It is clear to an influenza aficionado that stem mAbs typically do not utilize this IGHV, but the structure is not presented at this point in the text. Perhaps a few sentences of general IGHV usage for HA-directed mAbs would be useful here.

3 - Binding experiments, in general – it is not clear what values are plotted. For ELISAs (e.g. Fig 1 b,c; Fig 2 all panels) it looks like single experiments with no replicas. This should be stated.

4- Figure 2 should be summed up as a table (typically as a heatmap) and the individual binding curves put into Supplementary.

5 - Figure 3 – it seems as if 28-12 protects better against an H3N2 than against an H1N1 – how do you explain this?

6- Were the HAs made as HA0 or were they activated? Any differential binding especially for H1 HAs since the epitope spans the HA1/HA2?

7 - I don't understand why the authors use the word pseudoatomic for their model. It's an atomic model from a cryo-EM map at near-atomic resolution.

Reviewer #3 (Remarks to the Author):

The manuscript " Unique binding pattern for a lineage of human antibodies with broad reactivity against influenza A virus" by Xiaoyu Sun et al. has tried to understand how naturally occurring human antibodies recognize H1 and H3 subtypes of annually circulating influenza viruses. The authors isolated six independent antibodies cross-reacting with H3N2 and H1N1 HA and selected four antibodies with neutralization activity. The sequence analysis of BCR indicated that all four clones sheared the identical VH and VL carrying VH3-48/DH2-2/JH6 and VK1-12/JK5 gene. The authors focused on the clone 28-12, which showed resemble binding pattern with the control antibodies contained cross-reactivity, MEDI8852 and 39.29. In the second part, the authors did the structural-based analysis to understand the recognition pattern of 28-12 to H3 and H1. Helix A is a critical element for 28-12 to recognize both H3 and H1. However, N49HA2 is essential for H3 HA, but not H1 HA, binding to 28-12, indicating H3 needs epitope residues outside helix A to interact with 28-12. Then, the authors proposed that 28-12 mainly by recognizing a contiguous epitope of H3N2 HA, and a conformational epitope of H1N1 HA. In the end, the authors concluded that the antibody-containing germline sequence of VH3-48/DH2-2/JH6 and VK1-12/JK5 would be initially selected by H3 and evolved via somatic hypermutation to cross-neutralize H1 virus. Overall, the manuscript is very well written. The experiments and observations are conducted and explained in a logical fashion. Nonetheless, it remains unclear how specific antibodies acquire the cross-reactivity. This reviewer concerns about how somatic hypermutation contributes to the cross-neutralizing antibodies. Therefore, few issues need to be addressed before this paper can be considered suitable for publication to Nature communication.

Major concerns,

1）The authors isolated six independent antibodies cross-reacting with H3N2 and H1N1 HA from an individual vaccinated with the trivalent inactivated vaccine. However, a detailed vaccination schedule in this individual remains unclear, and whether trivalent inactivated vaccines have contained H3 and H1 was also unclear. If the trivalent inactivated vaccines contained both H3 and H1 antigens, how the authors concluded that these antibodies were initially selected by H3. The authors also need to declare how antigen-specific B cells were isolated from PBMC.

2）The authors showed that all isolated four clones were carrying VH3-48/DH2-2/JH6 and VK1-12/JK5 genes. The authors should indicate whether this is a unique case only observed in this

particular individual or this generally happened when the trivalent inactivated vaccine was used for immunization.

3）The authors concluded that H3 would initially select the germline-type antibody of VH3-48/DH2-2/JH6 and VK1-12/JK5. If so, the germline-type antibody also binds to H3 but not H1. The authors should show the binding pattern of the germline sequence of VH3-48/DH2-2/JH6 and VK1-12/JK5.

Response to Reviewer 1's comments

In this study Sun et al. present a lineage of broadly neutralizing antibodies (Bnabs) that neutralize group 1 and 2 strains. Amongst four members of the lineage that shared the same germ-line VH3-48/VK1-12, Bnab 28-12 was the most potent and further characterized. Employing biochemical and structural analyses, the authors discovered that 28-12 has distinct binding modes for H3 (group 2) and H1 (group 1) viruses, binding a continuos epitope within H3 but requiring conformational binding for H1. Analysis of germline-reverted Ab and intermediate mutants suggests that 28-12 was initially selected against an H3 strain and then evolved to have H1 binding ability. Overall, this is an intriguing result, but there are several aspects that deserve further attention.

Specific points:

1, A comprehensive comparison to other bnAbs that interact with Helix A would be important to include throughout the mansucript. As the authors particularly underline that their mAb has a long CDRH3 (23aa) they should include bnAb 3I14 in this comparison as this mAb appears to have similar features, including a 23aa CDRH3. The last part of the introduction contains a very long summary of the results, which are again summarized in the discussion. The actual discussion part is however relatively short. A more in depth discussion how 28-12 compares to existing bnAbs would be important.

Answer:

Thanks for the reviewer's valuable suggestion. We fully agree with the reviewer that a more in- depth discussion on how 28-12 compares to existing bnAbs would be important. Following the reviewer's suggestion, we have added a comprehensive comparison of 28-12 to other representative bnAbs that interact with helix A, including CR9114, CR6261, 39.29, CT149, FI6v3 and 3I14.

The Germlines V_H1-69, V_H1-18, and V_H3-30 have shown biased use for the

stem-directed bnAbs, for example, V_H1-69 for CR9114[1] and CR6261[2], V_H1-18 for CT149[3], V_H3-30 for 3I14[4], FI6v3[5] and 39.29[6]. 28-12 is encoded by V_H3-48 germline gene, which is rarely reported and may work differently from the existing bnAbs **(Supplementary Fig. 7a)**.

Comparison of 28-12 with previously reported cross-group anti-HA stem bnAbs revealed that these mAbs recognize an overlapping region in HA stem. However, the approaching angle for each mAb towards the HA stem region is different. Compared with 28-12/H1 complex, the Fabs for CR6261, CR9114 and FI6v3 are rotated clockwise by 66.2, 80.1 and 39.3°, respectively. While for 39.29, CT149 and 3I14, the Fabs are rotated counterclockwise by 49.1,73.4 and 16.5°, respectively, indicating the diversity of antibody binding. Although 28-12 epitope overlaps with other reported stem-directed mAbs, their detailed binding sites and interactions are distinct. For one single HA protomer (for the primary protomer), antibodies CR6261, CT149, 39.29, CR9114 and FI6v3 epitopes all span HA1 and HA2 regions, while 3I14 only binds HA2. However, for the primary HA protomer, here 28-12 shows different binding dependency on HA1 epitope residues (V40, S299 and L300), which is important for binding to H1N1 HA, but not to H3N2 HA **(Supplementary Fig. 7 and Fig. 4c-e)**. The cross-binding on HA1 and HA2 on one protomer probably makes the epitope on H1 HA more conformational. Consistent with the structural analysis, our biochemical assay revealed that both 28-12 and 3I14, the non-HA1/HA2 spanning mAbs (for the HA primary protomer) showed binding activity to the continuous epitope of H3 peptide $36\text{-}57_{HA2}$, but not to H1 **(Supplementary Fig. 8)**. A critical reason for this unique binding feature could also be attributed to residue $N49_{HA2}$ in H3 for both 28-12 and 3I14, which is $T49_{HA2}$ in H1 **(Fig. 5a)**. Of noting, 28-12 displayed different germline with 3I14 (V_H3-30) and engaged less epitopes in the adjacent protomer **(Supplementary Fig. 7)**. Thus, 28-12 represents a unique example among the helix A-targeting bnAbs.

In addition, we also compared the contribution of heavy/light chains and CDR loops on antibody recognition. Similar to 3I14, CT149, FI6v3 and 39.29, 28-12 uses

both heavy chain and light chain to recognize HA protein with prominent contributions of the heavy chain **(Supplementary Fig. 9)**. 28-12 has a long HCDR3, which makes much more extensive interaction with HA than other bnAbs. As the reviewer suggested, we also compared the HCDR3 between 28-12 and 3I14 which both have a long HCDR3 (23aa) in length **(Supplementary Fig. 10a)**. 3I14 HCDR3 mainly forms hydrophobic interactions and hydrogen-bonds with helix A and the fusion peptide of H3 or H6 HAs, which is similar to 28-12 HCDR3 in recognizing H3 HA. In addition, 28-12 HCDR3 also contacts with several HA1 residues of H1 HA on the primary protomer. It seems that the HCDR3 of 28-12 is more significant than 3I14 to recognize HA as it contributes more amino acids to interact with H3N2 HA (12) and H1N1 HA (11), respectively, while 3I14 devotes only 7 HCDR3 amino acids to interact with H1 or H6 HAs **(Supplementary Fig. 10b-c)**.

In conclusion, 28-12, encoded by the rarely reported germline gene VH3-48 is special as it showed different binding pattern between group 1 H1 and group 2 H3 HAs. The helix A-targeting bnAbs contribute differently to bind HA proteins with overlapping but distinct epitopes and multiple approaching angles towards the HA stem region. For some bnAbs such as 3I14, compared with other structural elements, the longer HCDR3 contributes much more interactions with the epitopes on HA. Among these bnAbs, 28-12 is unique as it devotes much more HCDR3 amino acids to react with HA. The HCDR3 of 28-12 might be developed as a potential template to produce small proteins or peptide-based antivirals. **(Main text, lines 304-347; Supplementary data, lines 61-89)**

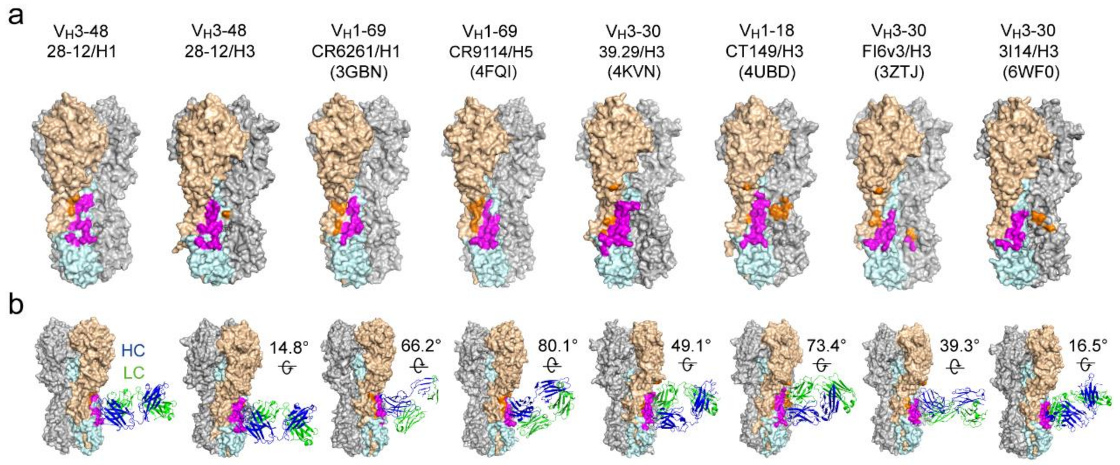

Supplementary Figure 7. Comparison of epitope and approaching angle for bnAbs that interact with Helix A. a, The epitope residues on the HA. HA is displayed in surface representation with HA1 and HA2 from the primary protomer in wheat and light blue, respectively. The epitope residues in HA1 are colored in orange and those in HA2 are colored in magenta. The color scheme is followed throughout. The V_H germline and PDB number of each bnAb is also listed. **b,** The change of the Fab approaching angle compared with that of 28-12/H1. Each Fab is shown in cartoon representation with the heavy chain (HC) and light chain (LC) colored in blue and green, respectively.

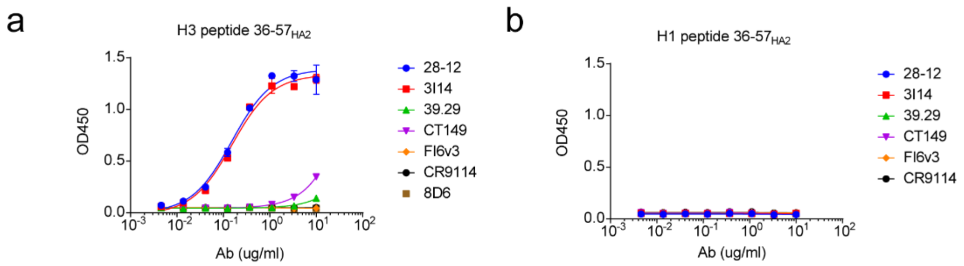

Supplementary Figure 8. The binding pattern of helix A-targeting bnAbs to the peptides 36-57$_{HA2}$ of H1 and H3. a-b, The reactivity of multiple reported stem bnAbs to peptide 36-57$_{HA2}$ of H3N2 **(a)** and H1N1 **(b)**.

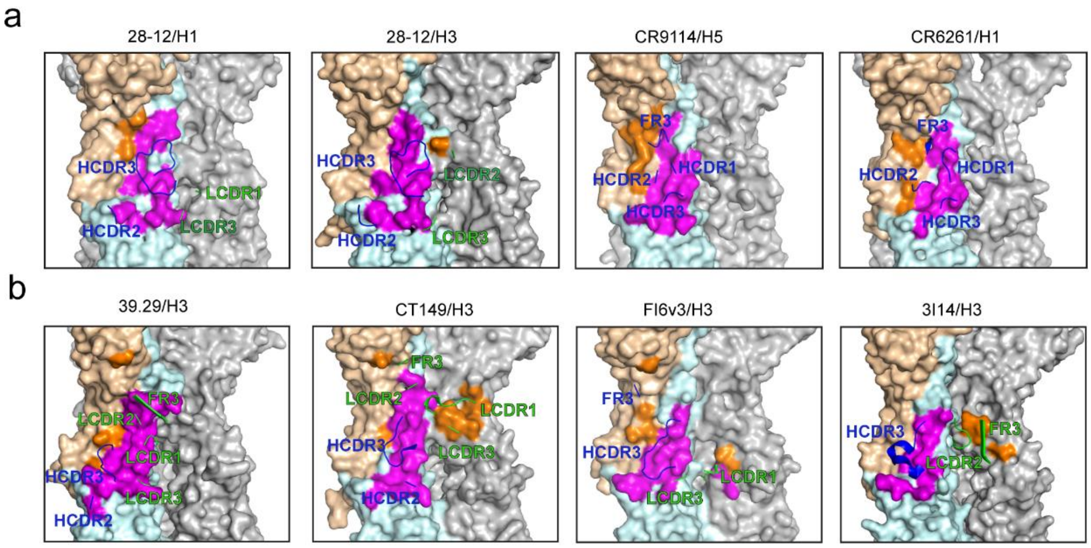

Supplementary Figure 9. Interactions between 28-12 and H3/H1 HAs and comparison with other helix A bounding antibodies. The epitope residues in HA1 are colored in orange and those in HA2 are colored in magenta, with the heavy chains in blue and light chains in green.

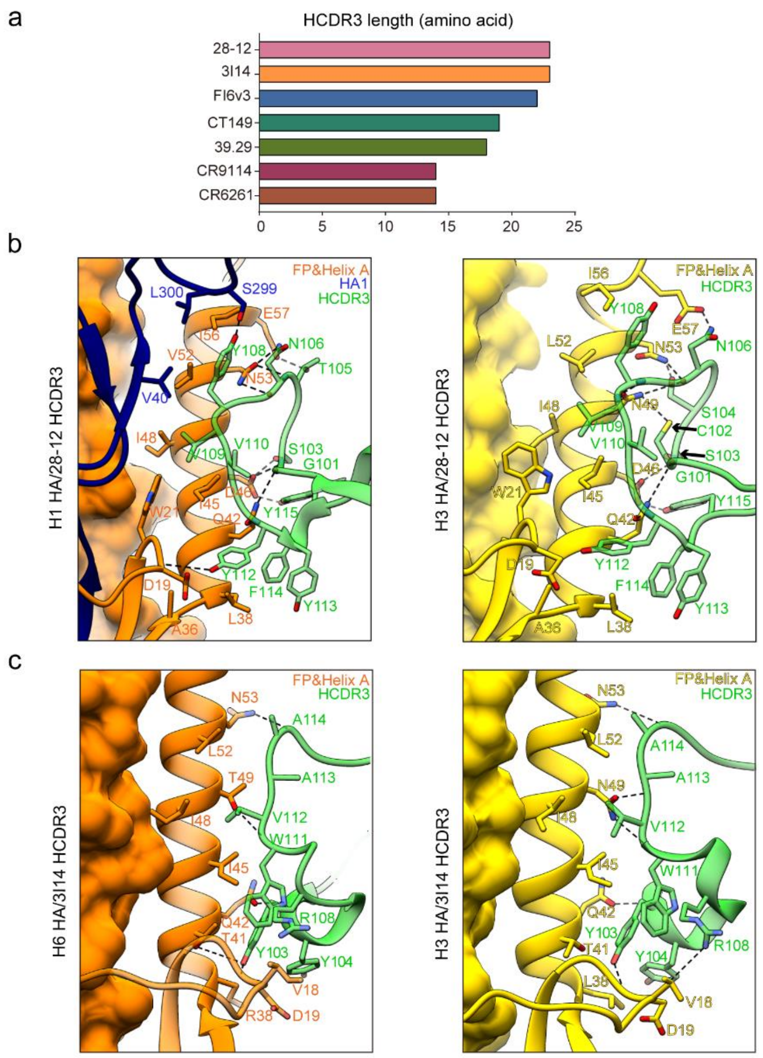

Supplementary Figure 10. The interaction network between HA and the HCDR3 of 28-12 or 3I14. a, The length of HCDR3 of the helix A-targeting bnAbs. **b,** The interactions network between HCDR3 of 28-12 and group 1 H1 or group 2 H3 HAs. **c,** The interactions network between HCDR3 of 3I14 and group 1 H6 or group 2 H3 HAs. HCDR3 is shown in cartoon and colored in green. HA1 is shown in dark blue. The fusion peptide and helix A (HA2) of group 1 and group 2 is colored in orange and gold, respectively. Residues that make either hydrogen bonds or hydrophobic interactions are shown as sticks. Dashed lines indicate hydrogen bonds.

2, To ease the reader, the full IAV strains tested should be introduced on first mentioning and noted which strains are human infecting and which not.

A：Thanks for the reviewer's valuable suggestions. We have modified the description

of the IAV strains on first mentioning in the main text and Figure 2. The hosts of non-human infecting IAV strains have been noted in the full name of IAV strains, while the other IAV strains without host annotation are human infecting which is illustrated in the Figure 2 legend. **(Main text, lines 109, 112, 135-140, 686-694)**

3, If I understand the presented data correctly, 19 antibodies from this donor were successfully sorted and cloned using. Four of these belonged to the same lineage. This seems a very high proportion. Was the sample collected shortly after vaccination? The authors should try to quantify the contribution of this lineage further eg by bulk heavy chain NGS of HA bait sorted cells.

Answer:

The sample was collected ~4 weeks after vaccination with seasonal trivalent influenza vaccine in 2016. We quantified the contribution of this V_H germline in broad-reactive B cells by analyzing four reported datasets. As shown in the following table, antibodies using V_H3-48 germline are rare with 0.155% (1 out of 647) in Joyce et al. (2016)[7], 4.575% (14 out of 306) in Andrews et al. (2017)[8], 1.818% (1 out of 55) in McCarthy et al. (2018)[9] and 2.381% (1 out of 42) in Andrews et al. (2015)[10]. Totally, 1.619% (17 out of 1050) cross-reactive B cells utilized the VH3-48 germline genes among all four analyzed datasets. However, antibodies using both V_H3-48 and V_K1-12 germline genes are not found in the four analyzed datasets. We further analyzed the frequency of V_H3-48 and V_K1-12 germline genes in the B cell repertoires of healthy donors with paired heavy chain and light chain using a dataset from DeKosky et al., which contained 134,345 sequences in total from three healthy donors[11]. The frequency of V_H3-48 germline genes in healthy donors (2.144%-4.684%) is similar with that in the HA cross-reactive B cell repertoires from the vaccinated donors (0.155%-4.575%). B cells engaging V_H3-48 and V_K1-12 concurrently are extremely rare with frequency ranging from 0.060% to 0.078% (**Reply Table 1**). We thus speculate that V_H3-48 &V_K1-12 germline genes engage a low frequency in human B cell repertoire and the donor in this study maybe a special case with a high proportion of this lineage antibodies. **(Main text, lines 348-356)**

Reply Table 1. The frequency of VH3-48 germline genes in the B cell repertoire from vaccinated or healthy donors

Ref	Donor vaccination	HA reactive specificity	Total Ab	VH3-48 Ab	VH3-48 & VK1-12 Ab
Joyce et al., 2016, 6 subjects	H5N1 DNA/MIV-prime-boost flu vaccine trial	H5 and H3 HAs	647	1 (0.155%)	0
Andrews et al., 2017, 14 subjects	H7N9 MIV vaccination	H1/H3/H7	306	14 (4.575%)	0
McCarthy et al., 2018, 3 subjects	2014-15 TIV or 2015-16 TIV	H1/H3	55	1 (1.818%)	0
Andrews et al., 2015	n	HA stem-specific B cells	42	1 (2.381)	0
DeKosky et al., 2014	healthy donor 1	not determined	129097	4908 (3.802%)	77 (0.060%)
	healthy donor 2	not determined	53679	1151 (2.144%)	22 (0.041%)
	healthy donor 3	not determined	15372	720 (4.684%)	12 (0.078%)

4, Fig1: The methods and legend do not state which HA as was used as bait. Strain/Group needs to be added.

Answer:

We are sorry for the inaccurate description. We used HA proteins from a group 2 strain A/HongKong/01/1968 H3N2 as the bait. The manuscript has been modified. **(Main text, lines 108-109, 426-427)**

5, There is very little information on the donor provided: "Blood was collected from a female volunteer previously inoculated with seasonal split influenza vaccine after she had signed the informed consent form." The authors need to specific the precise vaccine (manufacturer, year, which precise HA antigens were included). Time between vaccination and sampling should be stated. Was this the first (ever) vaccination that the person received? History of vaccination and prior IAV infection should be listed. How was the neutralization capacity of the donor plasma?

Answer:

We are sorry for the inaccurate description and we have further modified the description. We collected blood from a female volunteer ~4 weeks after vaccination with the seasonal trivalent influenza vaccine produced by Shanghai Institute of

Biological Products Co., Ltd. in 2016, which contains three components, A/California/7/2009(H1N1) pdm09-like virus, A/Victoria/361/2011(H3N2)-like virus and B/Wisconsin/1/2010-like virus. According to the description of the donor, she has been living in urban area of a city and this was her first influenza vaccination. The prior IAV infections of the donor is not clear, as influenza test was not included in the standard outpatient process. **(Main text, lines 106-107, 417-422)**

Following the reviewer's suggestion, we have tested the neutralization capacity of the donor's plasma using A/Jiangxi-Donghu/316/2006 H3N2 (JD/06 H3N2) and A/Sichuan/01/2009 H1N1 (SC/09 H1N1) viruses. The donor's plasma showed neutralizing capacity against both SC/09 H1N1 and JD/06 H3N2 with 50% neutralizing titer (NT50) of 31702 and 841.8, respectively, which indicates the possibility of presence of cross-reactive antibodies in this donor **(Reply Fig.1)**.

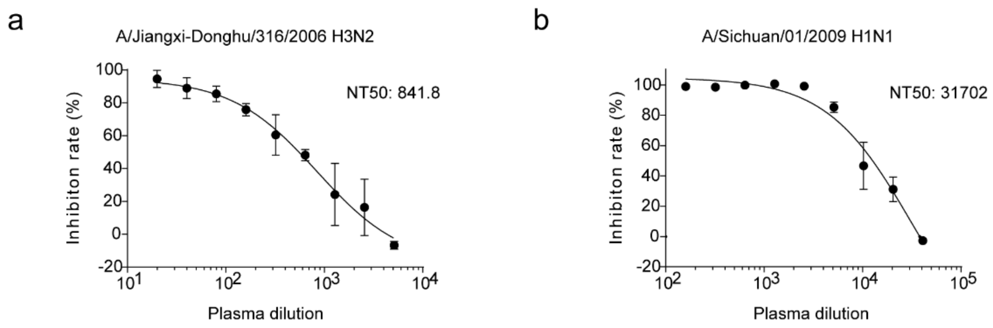

Reply Fig.1. The neutralizing activity of the donor's plasma against H1N1 and H3N2 viruses in MDCK cells. 50% neutralizing titers (NT50) was analyzed by GraphPad Prism 6. Representative data are shown with two replicates from two independent experiments. Results are depicted as the mean ± SD.

6, Line 115-117: The authors state: "The results showed that 28-2, 28-4, 28-6, and 28-12 exhibited cross-neutralizing activity against H1N1 and H3N2, with IC50 values ranging from 0.6382 μg/ml to 7.358 μg/ml (Fig. 1d)." however, according to figure 1d, Abs 28-8, 28-15, 28-16, 28-19, 28-27, and 28-28 also show cross-neutralization. Why where these not followed up further? So in all only 7 of the 19 analyzed Abs did not show cross neutralization activity. This is quite astonishing that so many of the

antibodies are neutralizing. The authors should discuss this and also include published bnAbs as reference in their analysis.

Answer:

We didn't follow up the other cross-reactive antibodies due to their relatively lower neutralizing activities compared with antibody 28-12. We mainly focus on 28-2, 28-4, 28-6, and 28-12 as they are unique by engaging the same germline genes, V_H3-48/V_K1-12, which is rarely reported and their neutralizing activities are relatively higher than other antibodies isolated in this project, especially for 28-12.

Regarding why "so many of the antibodies are cross-neutralizing" in this study. As from the published paper, Kevin R. McCarthy reported that memory B cells that cross-react with group 1 and group 2 influenza A viruses are abundant in adult human repertoires. Most KEL01 memory B cells (89/91) were specific for H3 WI-05 and other H3 subtypes, but two (2/91; 2%) avidly bound H3, H1, and H5 HAs. Most mem B clones from donor KEL03 were H3 specific, but 13% of them also bound H1. For subject KEL06, over one-third of the H3+IgG+ mem B cells (18/49) also bound HAs from H1 subtype[9]. We thus consider the percentage of the cross-group reactive memory B cells varied case by case. The donor in our study may happen to contain more cross-H1 and H3 memory B cells.

Additionally, following the reviewer's suggestions, we have included published bnAbs MEDI8852 and 39,29 as reference in the neutralizing analysis **(Figure 2b)** and CR9114, CR6261, CT149, 39.29, FI6v3 and 3I14 as reference in the structural analysis **(Supplementary Figs 7-10)**.

7, Figure 1d: the full neutralization data and IC50 should be shown.

Answer:

Thanks for the reviewer's suggestion. The full neutralization data has been shown in **Source data** and the IC50 values have been presented in **Figure 1b**.

8, To underline the claim that 28-12 recognizes a novel epitope the authors should conduct competition mapping and/ or docking analyses with other bnAbs.

Answer:

Thanks for the reviewer's valuable suggestion. We have compared the epitope, approaching angles towards HA, interacting heavy/light chain regions and HCDR3 interactions of 28-12 with other helix A-targeting bnAbs in **Supplementary figures 7-10** as shown in the reply to the first question. **(Main text, lines 304-358; Supplementary data, 61-89)**

9, Line 122/123 "…indicating a positive correlation between this lineage and neutralizing activity". This needs to be rephrased, no test for correlation has been performed

Answer:

We are sorry for the inaccurate description and we have modified the manuscript. **(Main text, lines 120-121)**

10, Line 135 "significant" cannot be used in this context as no statistical test was performed.

Answer:

Thanks for the reviewer's suggestion and we have deleted "significant" in the revised manuscript. **(Main text, lines 125-131)**

11, Line 167 Therapeutic dose needs to be stated in the text

Answer:

Thanks for the reviewer's suggestion and we have stated the therapeutic dose (25mg/kg) of antibody 28-12 in the main text and Figure 3 legend. **(Main text, line 166 and 706)**

12, Line 209 Typo: "In contrast" instead of "In contract"

Answer:

Thanks for the reviewer's suggestion and we have modified this description in the revised manuscript. **(Main text, line 204)**

13, Line 268 The subtitle needs to be shortened and rephrased. “was expected” does not fit in this context.

Answer:

Thanks for the reviewer’s suggestion. The subtitle is rephased into “Role of somatic mutations in shaping antibody 28-12” in the revised manuscript. **(Main text, line 265)**

14, Lines 309-312: “28-2, 28-4, 28-6 and 28-12 are unique, as they belong to the same lineage VH3-48/DH2-2/JH6 and VK1-12/JK5. The usage of germline gene VH3-48, together with previously reported influenza bnAb germline genes, indicates the diverse germline selection of broad-spectrum antibodies against IAVs.” This sentence needs to be rephrased to make sense.

Answer:

Thanks for the reviewer’s suggestion. We have modified the description in the revised manuscript. “28-2, 28-4, 28-6 and 28-12 all belong to the same lineage V_H3-48/D_H2-2/J_H6 and V_K1-12/J_K5, which is rarely reported. In addition to V_H3-48, bnAbs using V_H1-18, V_H1-69, V_H6-1 and V_H3-30 germline genes have been reported, which indicates the diverse germline selection of broad-spectrum antibodies against IAVs.” **(Main text, lines 304-308)**

Response to Reviewer 2’s comments

In this study, Sun, Liu, Lu and colleagues, describe a novel class of neutralizing antibodies. The authors use binding, neutralization and structural determination experiments to show that these Abs are relatively broad in their binding breadth, that they neutralize H1N! and H3N2 viruses and that they bind the HA stem epitope. Additionally, these antibodies utilize VH3-48/VK1-12 variable immunoglobulin genes – a hitherto undescribed combination for HA stem binding antibodies. Particularly interesting is the finding that a member of this lineage cross-reacts with

an H1 and an H3 HA and that its binding mode is slightly different on a different subtype, as it engages a single alpha-helix on an H3, but it requires a conformational epitope consisting of HA1 and HA2 subdomains.

While the structural information provided is very useful as it compares the binding to both an H1 (group 1 influenza) and an H3 (group 2), the study is limited to a single donor and it is unclear how prevalent this class of antibodies is in the general population. The donor's exposure history is not reported so it is impossible to appreciate whether these antibodies were generated under uncommon antigenic exposure conditions. Additionally, the authors call these antibodies broadly neutralizing, but the panel of viruses/HAs is quite limited. Indeed, the authors show that the Abs bind to heterosubtypic HAs, but binding to historical isolates needs to be demonstrated for the Abs to be defined as broadly neutralizing.

Answer:

Thanks for the reviewer's comments and valuable suggestions. We have modified the detailed description of the vaccine and vaccination procedure of the donor in the revised manuscript. In addition, we have carried out additional experiments to study the frequency of 28-12 epitope-targeting serum antibodies in 50 donors vaccinated with seasonal influenza vaccines in 2020. We also included additional HA subtypes (H6N2, H8N4, H9N2, H4N2 and H4N8), H1 historical isolates (A/Brevig mission/1/1918 H1N1, A/WSN/1933 H1N1 and A/Florida/04/2017 H1N1) and H3 historical isolates (A/Missouri/09/2014 H3N2 and A/Netherlands/178/1995 H3N2) for the binding affinity assay of V_H3-48/V_K1-12 antibodies.

Major concerns:

1, Lines 129-130 – "among which 28-4, 28-12 exhibited stronger binding activity than MEDI88529 and 39.29" – binding signal in ELISA does not equate to "stronger binding". For example, MEDI8852 saturates at lower concentration than 28-12. The authors should provide comparative Kd values, preferably from kinetic measurements.

Answer:

Thanks for the reviewer's suggestion. We have conducted additional biolayer interferometry using an Octet Red instrument to measure the Kd values of V_H3-48/V_K1-12 antibodies as shown in **Figure 2a** and **Source data**. **(Main text, lines 125-131 and 686-695)**

2, Do you know the exposure history of this individual? This would help corroborate the idea that this lineage was initiated with an H3N2 and affinity matured against an H1N1. In any event, a binding experiment against a panel of historical H3 and H1 HAs with both the mature antibody and the germline-reverted one needs to be performed.

Answer:

We are sorry for the incomplete description of the vaccination details of the donor recruited in this project. We collected blood sample from this donor ~4 weeks after vaccination with the seasonal trivalent influenza vaccine produced by Shanghai Institute of Biological Products Co., Ltd. in 2016, which contains three components, A/California/7/2009(H1N1) pdm09-like virus, A/Victoria/361/2011(H3N2)-like virus and B/Wisconsin/1/2010-like virus. According to the description of the donor, she has been living in urban area of the city and this was her first influenza vaccination. The prior IAV infections of the donor is not clear, as influenza test was not included in the standard outpatient process. **(Main text, lines 103-109 and 416-422)**

To figure out the original reactivity of the B cells engaging the germline genes of antibody 28-12, we performed additional binding experiments against a panel of historical H3 and H1 HAs with different versions of 28-12 in which all or individual somatic mutations in the V_H or V_L chain were reverted to the germline. The fully germline version of 28-12 reacted with a panel of tested historical H3 HAs, but failed to recognize the tested historical H1 HAs. H3/H1 HAs binding required somatic mutations in both heavy and light chains **(Figure 6b-c)**. These findings indicate that the naive B cell that generated 28-12 was specific for H3 HA and that the enhanced binding activity and the cross-reactivity to H1 HA was acquired through somatic

mutations.

(Main text, lines 266-276 and 761-762)

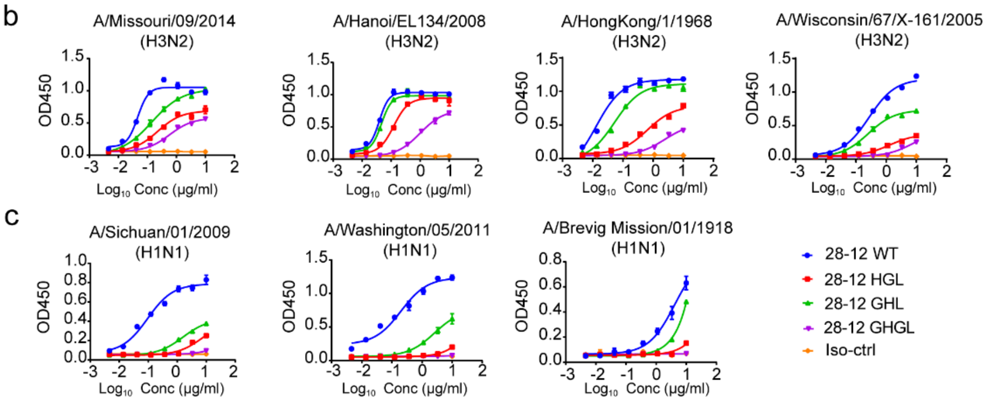

Figure 6b-c. Somatic mutations enhance the 28-12 reactivity and broaden its specificity. The ELISA-based reactivity of 28-12 germline variants to the HA proteins of historical H3N2 isolates **(b)** and historical H1N1 isolates **(c).** GHGL, germline VH paired with germline VL. HGL, matured VH paired with germline VL. GHL, germline VH paired with matured VL. Iso-ctrl, isotype control. Representative data are shown from two independent experiments. Results are depicted as the mean ± SD.

3, Were these antibodies tested for auto/polyreactivity? Stem mAbs are typically prone to polyreactivity (see for ex. PMID: 26631631 and PMID: 30837606) and this needs to be tested.

Answer:

Thanks for the reviewer's suggestion. We performed two experiments to study the polyreactivity of V_H3-48/V_K1-12 antibodies with two polyreactive positive control antibodies, an influenza HA stem mAb CR9114 and a prototypical lupus-associated autoantibody 3H9[10]. From the ELISA based assay, we found the V_H3-48/V_K1-12 mAbs and the germline mAb were not polyreactive to polyinosinic-polycytidylic acid (poly I:C), insulin, and lipopolysaccharide (LPS) compared to CR9114 and 3H9. Similarly, none of the V_H3-48/V_K1-12 mAbs bound at a significant level to HEp-2 cells by immunofluorescence while the control antibody 3H9 showed higher intensity of immunofluorescence. Although CR9114 is polyreactive, it doesn't appear to be significantly reactive to HEp-2 cells **(Supplementary Fig. 3)**. **(Main text, lines 155-**

158; Supplementary data, 22-31)

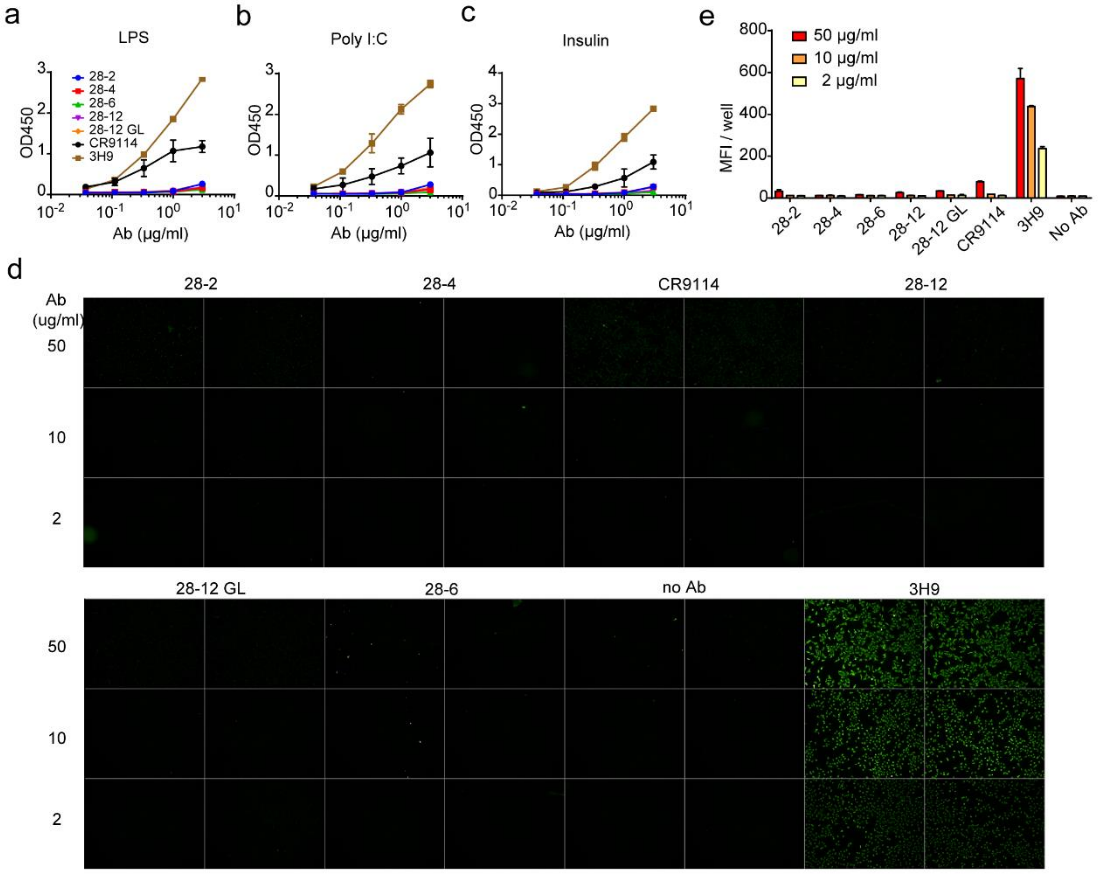

Supplementary figure 3. VH3-48/VK1-12 mAbs are not polyreactive. ELISA-based binding curves of each mAb to antigens LPS **(a)**, Poly I:C **(b)** or insulin **(c)**. 28-12 GL, the germline version of 28-12. CR9114, a reported influenza HA stem mAb with polyreactivity to above antigens. 3H9, high-affinity anti-nuclear antibody 3H9. **d,** Binding image of the each mAb at a concentration of 2, 10 or 50 μg/ml to HEp-2 cells by immunofluorescence **e,** Mean fluorescence intensity (MFI) of each mAb binding to HEp-2 cells by immunofluorescence. Values represent the average of two replicates. Representative data are shown from at least two independent experiments. Results are depicted as the mean ± SD.

Minor comments:

4, Figure 1 – legend is inverted – should say group 1 SC/09 H1N1 and group 2 HK/68 H3N2

Answer: We are sorry for our mistakes, and we have modified the description. **(Main**

text, line 677)

5, Line 119-120 "Unexpectedly, a large portion of antibodies utilized the VH3-48 germline genes" Why was it unexpected? It is clear to an influenza aficionado that stem mAbs typically do not utilize this IGHV, but the structure is not presented at this point in the text. Perhaps a few sentences of general IGHV usage for HA-directed mAbs would be useful here.

Answer:

Thanks for the reviewer's suggestion. We have included a few sentences of general IGHV usage for HA-directed mAbs in this section as follows: The identification of anti-HA bnAb categories that utilize the human IGHV1-69, IGHV1-18, IGHV3-30 and IGHV6-1 indicates the dominance of these germline genes. IGHV3-48 is unique as it is rarely reported for influenza bnAbs, which indicates germline gene usage is highly diversified among HA stem antibodies. **(Main text, lines 304-308)**

6, Binding experiments, in general – it is not clear what values are plotted. For ELISAs (e.g. Fig 1 b,c; Fig 2 all panels) it looks like single experiments with no replicas. This should be stated.

Answer:

Thanks for the reviewer's suggestion. We have summarized the antibody EC50s and IC50s as a table in **Figure 1b** and the ELISA binding and neutralizing curves have been put into **Source data**. For the binding curves, values represent the average of two replicates. Representative data are shown from two independent experiments. Results are depicted as the mean ± SD. In addition, as reviewer 1 suggested, we have replaced the ELISA binding experiments in **Figure 2a** with kinetic measurements showing with KD values. The manuscript has been modified. **(Main text, lines 673-696)**

7, Figure 2 should be summed up as a table (typically as a heatmap) and the individual binding curves put into Supplementary.

Answer:

Thanks for the reviewer's suggestion. We have replaced the ELISA binding experiments in Figure 2a with kinetic measurements showing as KD values. As the reviewer suggested, the KD values were summed up into a figure in **Figure 2a** and a table showing as a heatmap in the **Source data**. **(Main text, lines 686-696)**

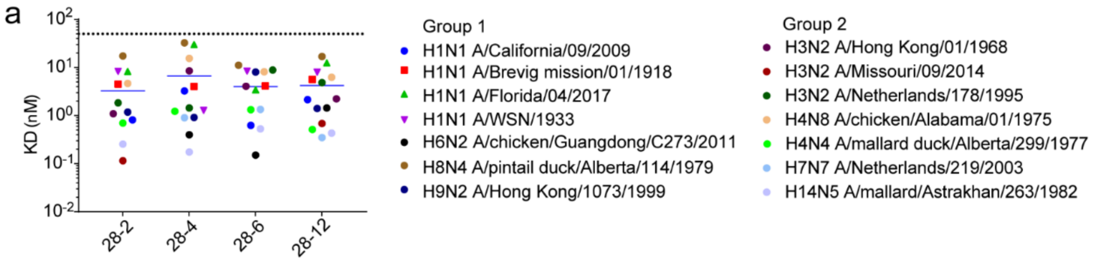

Figure 2a. Binding affinity (Kd) of each mAb to HAs from a panel of group 1 and group 2 influenza A isolates. as measured by BLI. Dashed line indicates 50 nM. Solid line indicates the mean KD values.

Source data. The Kd values (nM) of the V_H3-48/V_K1-12 linage antibodies.

Group	Subtype	Strains	28-2	28-4	28-6	28-12
1	H1N1	A/California/09/2009	0.812	3.24	0.625	2.14
		A/Brevig mission/01/1918	4.5	4.03	4.15	5.62
		A/Florida/04/2017	8.26	30	3.43	12.5
		A/WSN/1933	8.26	1.28	8.4	7.97
	H6N2	A/chicken/Guangdong/C273/2011	<0.001	0.398	0.15	1.44
	H8N4	A/pintail duck/Alberta/114/1979	17.3	32.50	11.1	16.9
	H9N2	A/Hong Kong/1073/1999	1.18	0.91	8.01	1.4
2	H3N2	A/Hong Kong/01/1968	1.09	8.47	4.07	2.22
		A/Missouri/09/2014	0.115	<0.001	<0.001	0.684
		A/Netherlands/178/1995	1.83	1.44	8.82	4.84
	H4N8	A/chicken/Alabama/01/1975	4.66	15.4	8.13	6.23
	H4N4	A/mallard duck/Alberta/299/1977	0.698	1.22	1.32	0.51
	H7N7	A/Netherlands/219/2003	<0.001	0.9	1.34	0.349
	H14N5	A/mallard/Astrakhan/263/1982	0.254	0.174	0.529	0.429

8, Figure 3 – it seems as if 28-12 protects better against an H3N2 than against an H1N1 – how do you explain this?

Answer:

We all used a lethal dose of HK/68 H3N2 and SC/09 H1N1 viruses in the animal experiments. The mice were intranasally challenged H1N1 virus with a dosage of 5

LD50/mouse. However, the original H3N2 virus stocks in our lab were just right to cause 100% death in mice, while dilution of the H3N2 virus stocks resulted in partial death. We speculate the dosage of H3N2 virus used in the mice experiment was 2~3 LD50/mouse. This may be a possible reason.

9, Were the HAs made as HA0 or were they activated? Any differential binding especially for H1 HAs since the epitope spans the HA1/HA2?

Answer:

That's a good question. The HAs used in our project were made as uncleaved HA0. We conducted additional ELISA-based experiments to study "whether there is any differential binding especially for H1 HAs since the epitope spans the HA1/HA2". The HA0 of both H3N2 and H1N1 can be cleaved under TPCK treatment. Cleavage of HA0 does not impair the binding activity of 28-12 to H3N2 HA, but significantly affects the reactivity to H1N1 HA. We also included other HA stem mAbs CR9114, FI6v3, CT149 and 39.29, whose epitope span the HA1/HA2 on the primary protomer in both H1N1 and H3N2 HAs for comparison. 3I14 was another control mAb with epitopes only in the HA2 region (for the primary protomer) of both H3N2 and H6N2 HAs. The results showed that Cleavage of HA0 doesn't affect the binding activity of all the antibodies tested to H3N2, but obviously impairs the reactivity to H1N1 **(Ref Figure 2)**. We hypothesis that H1N1 HA is more sensitive to protease, which may cause conformational change and destroy the complete epitope in H1 HA, thus resulting in loss of binding to antibodies. However, cleavage of H3N2 HA may not change the overall epitope structure, therefore the cleaved H3N2 HA still maintains the reactivity to antibodies. These differences between H1 and H3 HAs may be attributed to the distinct intrinsic properties of HAs, which is not unique for 28-12, but for most of the HA stem mAbs. Although this point is interesting, we didn't include these data in the revised manuscript as it needs more further study to illustrate the different working mechanisms between H1 and H3 HAs.

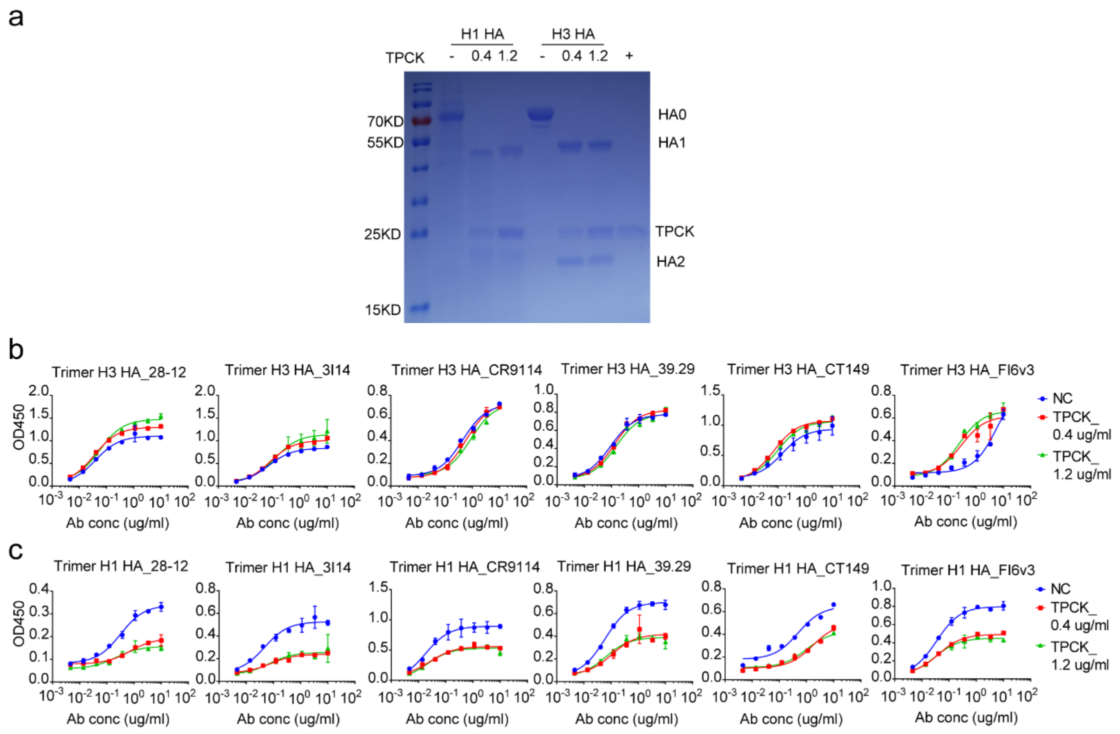

Ref Figure 2. Differential binding activity of HA stem mAbs to the cleaved HAs between H1 and H3 subtypes. a, Cleavage of HA0 run by SDS-PAGE. **b- c,** ELISA-based binding activity of each HA stem mAb to HA0 or cleaved HA of H1N1 **(b)** or H3N2 **(c)**. HA0 proteins (200 ng/well) coated onto the ELISA plates were treated with TPCK (40 or 120 ng/well) for 15min at room temperature. After blocking with 1%BSA and washing three times with PBST, the plates were incubated with serial diluted antibodies, followed by detection with an anti-human IgG Fc-HRP antibody.

10, I don't understand why the authors use the word pseudoatomic for their model. It's an atomic model from a cryo-EM map at near-atomic resolution.

Answer:

Thanks for the comment from our reviewer. Traditionally, since the cryo-EM maps were usually limited to intermediate or low resolutions, the models built against those cryo-EM maps were termed "pseudoatomic model". In our original manuscript, we used this traditional name. The reviewer is right, since our cryo-EM maps are now at near-atomic resolutions, we then decide to use "atomic model" in the revised manuscript.

Response to Reviewer 3's comments

The manuscript " Unique binding pattern for a lineage of human antibodies with broad reactivity against influenza A virus" by Xiaoyu Sun et al. has tried to understand how naturally occurring human antibodies recognize H1 and H3 subtypes of annually circulating influenza viruses. The authors isolated six independent antibodies cross-reacting with H3N2 and H1N1 HA and selected four antibodies with neutralization activity. The sequence analysis of BCR indicated that all four clones sheared the identical VH and VL carrying VH3-48/DH2-2/JH6 and VK1-12/JK5 gene. The authors focused on the clone 28-12, which showed resemble binding pattern with the control antibodies contained cross-reactivity, MEDI8852 and 39.29. In the second part, the authors did the structural-based analysis to understand the recognition pattern of 28-12 to H3 and H1. Helix A is a critical element for 28-12 to recognize both H3 and H1. However, $N49_{HA2}$ is essential for H3 HA, but not H1 HA, binding to 28-12, indicating H3 needs epitope residues outside helix A to interact with 28-12. Then, the authors proposed that 28-12 mainly by recognizing a contiguous epitope of H3N2 HA, and a conformational epitope of H1N1 HA. In the end, the authors concluded that the antibody-containing germline sequence of VH3-48/DH2-2/JH6 and VK1-12/JK5 would be initially selected by H3 and evolved via somatic hypermutation to cross-neutralize H1 virus.

Overall, the manuscript is very well written. The experiments and observations are conducted and explained in a logical fashion. Nonetheless, it remains unclear how specific antibodies acquire the cross-reactivity. This reviewer concerns about how somatic hypermutation contributes to the cross-neutralizing antibodies. Therefore, few issues need to be addressed before this paper can be considered suitable for publication to Nature communication.

Major concerns,

1, The authors isolated six independent antibodies cross-reacting with H3N2 and H1N1 HA from an individual vaccinated with the trivalent inactivated vaccine.

However, a detailed vaccination schedule in this individual remains unclear, and whether trivalent inactivated vaccines have contained H3 and H1 was also unclear. If the trivalent inactivated vaccines contained both H3 and H1 antigens, how the authors concluded that these antibodies were initially selected by H3. The authors also need to declare how antigen-specific B cells were isolated from PBMC.

Answer:

We are sorry for the incomplete description on the detailed vaccination schedule of this individual and we have further modified the manuscript. We collected blood sample from a female adult ~4 weeks after vaccination with the seasonal trivalent influenza vaccine produced by Shanghai Institute of Biological Products Co., Ltd. in 2016, which contains three components, A/California/7/2009(H1N1) pdm09-like virus, A/Victoria/361/2011(H3N2)-like virus and B/Wisconsin/1/2010-like virus. This was the first influenza vaccination that the donor has ever received. The prior IAV infections of the donor is not clear, as influenza test was not included in the standard outpatient process. This donor may have been infected by IAV many times according to the frequency of flu-like symptoms described by her. Fresh PBMCs were isolated from the collected blood sample by using the Ficoll-Paque gradient. Single CD19+IgG+ HK/68 H3 HA + specific memory B cells were isolated with a BD Influx^{TM} Cell Sorter. Heavy- and light-chain genes encoding each mAb were cloned and expressed transiently in CHO cells. The mAbs were screened by ELISA and microneutralization assays against group 1 SC/09 H1N1 and group 2 HK/68 H3N2 viruses. We have added this description in the main text. **(Main text, lines 103-114 and lines 415-431)**

As the reviewer interested in "how the authors concluded that these antibodies were initially selected by H3", we conducted additional experiments to figure out the original reactivity of the B cells engaging the germline genes of antibody 28-12. ELISA-based binding activity of a panel of historical H3 and H1 HAs to different versions of 28-12, in which, all or individual somatic mutations in the VH or VL chain were reverted to the germline. The fully germline version (GHGL) of 28-12 reacted with a panel of tested historical H3 HAs, but failed to recognize the tested

historical H1 HAs. H3/H1 HAs binding required somatic mutations in both heavy and light chains **(Figure 6b-c)**. These findings indicate that the naive B cell that generated 28-12 was specific for H3 HA and that the cross-reactivity to H1 HA and the enhanced reactivity to both H1 and H3 were acquired through somatic mutations. **(Main text, lines 266-274 and lines 758-764)**

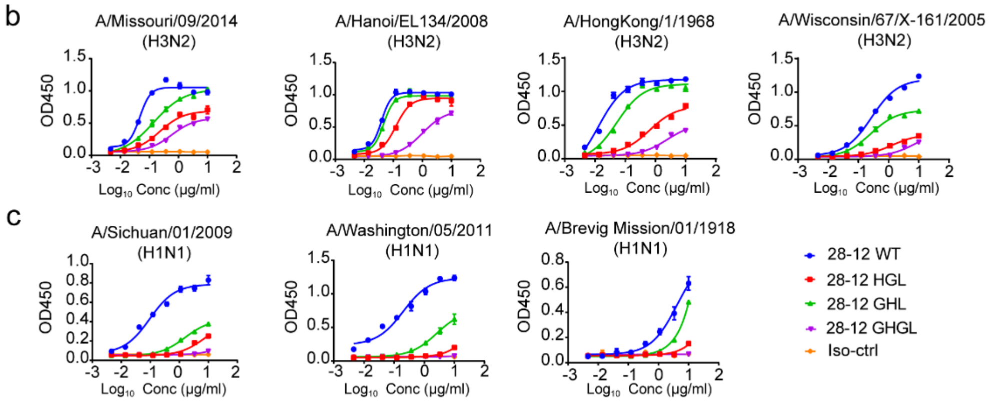

Figure 6b-c. Somatic mutations enhance the 28-12 reactivity and broaden its specificity. The ELISA-based reactivity of 28-12 germline variants to the HA proteins of historical H3N2 isolates **(b)** and historical H1N1 isolates **(c).** GHGL, germline VH paired with germline VL. HGL, matured VH paired with germline VL. GHL, germline VH paired with matured VL. Iso-ctrl, isotype control. Representative data are shown from two independent experiments. Results are depicted as the mean ± SD.

2, The authors showed that all isolated four clones were carrying VH3-48/DH2-2/JH6 and VK1-12/JK5 genes. The authors should indicate whether this is a unique case only observed in this particular individual or this generally happened when the trivalent inactivated vaccine was used for immunization.

Answer:

Thanks for the reviewer's suggestion. To answer this question, we first quantified the contribution of this lineage in broad-reactive B cells by analyzing four reported HA specific datasets. As shown in the following table, antibodies using V_H3-48 germlines are rare with 0.155% (1 out of 647) in Joyce et al. (2016), 4.575% (14 out of 306) in Andrews et al. (2017), 1.818% (1 out of 55) in McCarthy et al. (2018) and 2.381% (1 out of 42) in Andrews et al. (2015). Totally, 1.619% (17 out of 1050) cross-

reactive B cells utilized the V_H3-48 germline genes among all four analyzed datasets. However, antibodies using both V_H3-48 and V_K1-12 germline genes are not found in the four analyzed datasets. We further analyzed the frequency of V_H3-48&V_K1-12 germline genes in the B cell repertoires of healthy donors with paired heavy chain and light chain using a dataset from DeKosky et al., which contained 134,345 sequences in total from three healthy donors. The frequency of V_H3-48 germline genes in healthy donors (2.144%-4.684%) is similar with that in the HA cross-reactive B cell repertoires from the vaccinated donors (0.155%-4.575%) **(Reply Table 1)**. B cells engaging V_H3-48 and V_K1-12 concurrently are rare with frequency ranging from 0.060% to 0.078%. We thus think this is a unique case. **(Main text, lines 348-356)**

In addition, to investigate how frequently the 28-12 epitope is targeted following vaccination, we tested the binding of sera from 50 donors vaccinated with seasonal trivalent inactivated vaccine in 2020 to peptide 36-57_{HA2} of H3N2 and H1N1. Of which, 10 sera showed higher binding activity to the peptide 36-57_{HA2} of H3N2 than H1N1. The donor LU, the source of 28-12, in this project was included as a positive control. As $N49_{HA2}$ is critical for 28-12 binding to H3N2, mutation ($N49T_{HA2}$) of which significantly destructs this reactivity of antibody 28-12 and sera LU. We only observed a notable reduced binding to H3N2 peptide 36-57_{HA2} with N49T mutation of two sera (41 and 47). To assess the importance of 28-12 epitope in controlling virus infection with sera 41 and 47, antibody depletion assays were performed against H3N2 peptide 36-57_{HA2}. H3 peptide-depleted sera reduced the ability to neutralize H3N2 virus infection, as compared with the unrelated control peptide-depleted sera **(Supplementary Fig. 11)**. These data indicate the 28-12 epitope-targeting serum antibodies in vaccinated human donors can be found but exist as a relatively low frequency. **(Main text, lines 356-358; Supplementary data, lines 90-101)**

Reply Table 1. The frequency of VH3-48 germline genes in the B cell repertoires from vaccinated or healthy donors

Ref	Donor vaccination	HA reactive specificity	Total Ab	VH3-48 Ab	VH3-48 & VK1-12 Ab
Joyce et al., 2016, 6 subjects	H5N1 DNA/MIV-prime-boost flu vaccine trial	H5 and H3 HAs	647	1 (0.155%)	0
Andrews et al., 2017, 14 subjects	H7N9 MIV vaccination	H1/H3/H7	306	14 (4.575%)	0
McCarthy et al., 2018, 3 subjects	2014-15 TIV or 2015-16 TIV	H1/H3	55	1 (1.818%)	0
Andrews et al., 2015	n	HA stem-specific B cells	42	1 (2.381)	0
DeKosky et al., 2014	healthy donor 1	not determined	129097	4908 (3.802%)	77 (0.060%)
	healthy donor 2	not determined	53679	1151 (2.144%)	22 (0.041%)
	healthy donor 3	not determined	15372	720 (4.684%)	12 (0.078%)

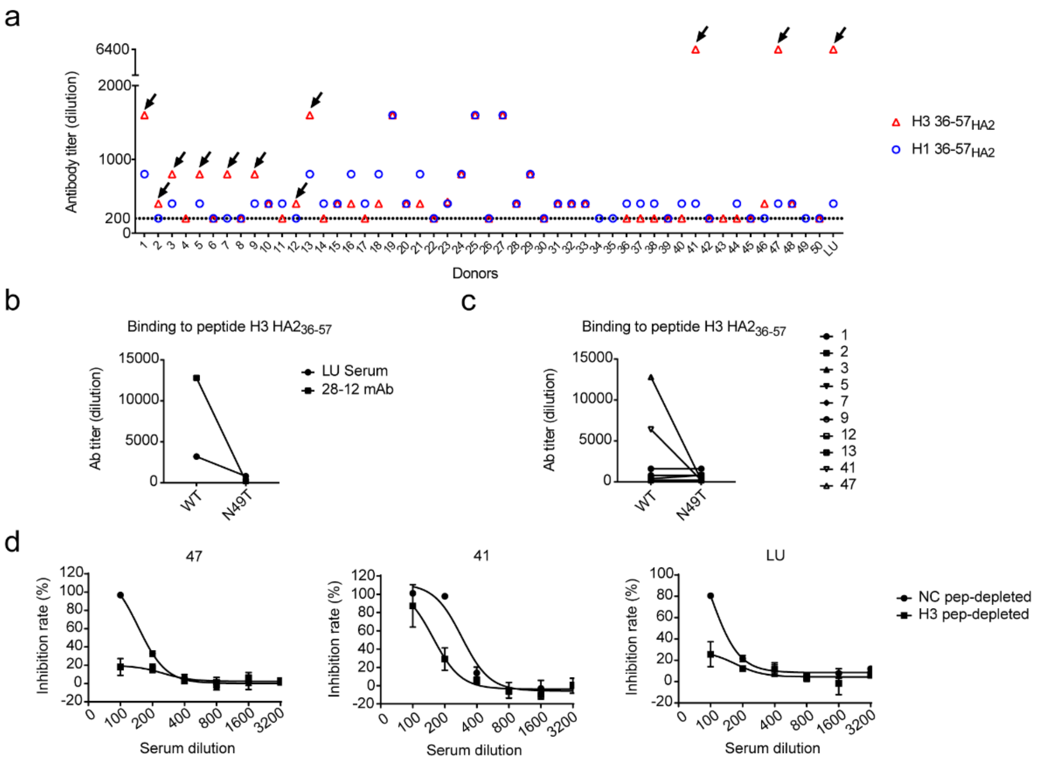

Supplementary Figure 11. Frequency of 28-12 epitope-targeting serum antibodies in vaccinated donors. a, ELISA-based binding activity of sera from 50 donors vaccinated with seasonal trivalent inactivated vaccine in 2020 to peptides 36-57_{HA2} of H3N2 and H1N1, showing as antibody titer. 10 sera showed higher binding activity to the peptide 36-57_{HA2} of H3N2 than H1N1, which is marked with black arrows. Serum LU, the source of 28-12, was the positive control. **b,** ELISA-based binding activity of antibody 28-12 and serum LU to H3N2 peptide 36-

57_{HA2} with N49T mutation as compared to WT peptide, showing as antibody titer. **c,** ELISA-based binding activity of 10 sera to H3N2 peptide 36-57_{HA2} with N49T mutation as compared to WT peptide. **d,** Neutralization curves of serial diluted H3 peptide-depleted sera against H3N2 virus as compared with the unrelated NC peptide-depleted sera. Figure is representative of two independent experiments. Data represent as mean±SD.

3, The authors concluded that H3 would initially select the germline-type antibody of VH3-48/DH2-2/JH6 and VK1-12/JK5. If so, the germline-type antibody also binds to H3 but not H1. The authors should show the binding pattern of the germline sequence of VH3-48/DH2-2/JH6 and VK1-12/JK5.

Answer:

To figure out the original reactivity of the B cells engaging the germline genes of antibody 28-12, we performed additional binding experiments against a panel of historical H3 and H1 HAs with different versions of 28-12 in which all or individual somatic mutations in the V_H or V_L chain were reverted to the germline. The fully germline version (GHGL) of 28-12 reacted with a panel of tested historical H3 HAs, but failed to recognize the tested historical H1 HAs. H3/H1 HAs binding required somatic mutations in both heavy and light chains **(Figure 6b-c)**. These findings indicate that the naive B cell that generated 28-12 was specific for H3 HA and that the enhanced binding activity and the cross-reactivity to H1 HA was acquired through somatic mutations. **(Main text, lines 266-274 and lines 758-764)**

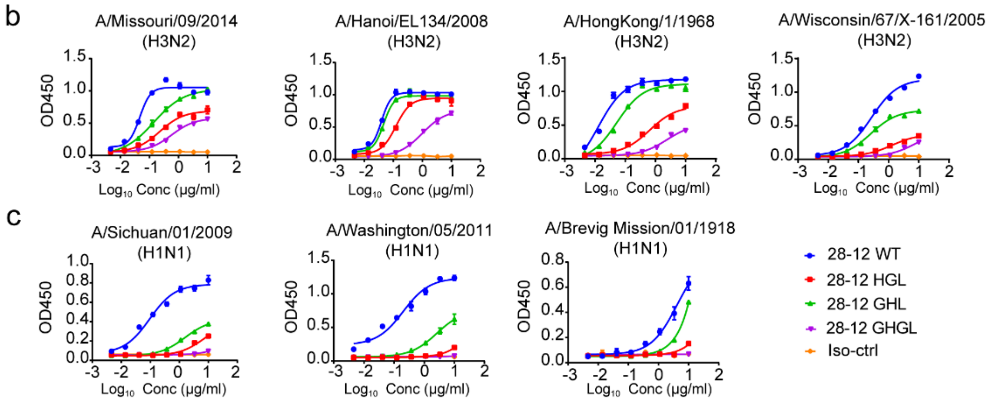

Figure 6b-c. Somatic mutations enhance the 28-12 reactivity and broaden its specificity. The

ELISA-based reactivity of 28-12 germline variants to the HA proteins of historical H3N2 isolates **(a)** and historical H1N1 isolates **(b).** GHGL, germline VH paired with germline VL. HGL, matured VH paired with germline VL. GHL, germline VH paired with matured VL. Iso-ctrl, isotype control. Representative data are shown from at least two independent experiments. Results are depicted as the mean ± SD.

References

1. Dreyfus, C., *et al.* Highly conserved protective epitopes on influenza B viruses. *Science* **337**, 1343-1348 (2012).
2. Ekiert, D.C., *et al.* Antibody recognition of a highly conserved influenza virus epitope. *Science* **324**, 246-251 (2009).
3. Wu, Y., *et al.* A potent broad-spectrum protective human monoclonal antibody crosslinking two haemagglutinin monomers of influenza A virus. *Nat Commun* **6**, 7708 (2015).
4. Harshbarger, W.D., *et al.* Unique structural solution from a VH3-30 antibody targeting the hemagglutinin stem of influenza A viruses. *Nat Commun* **12**, 559 (2021).
5. Corti, D., *et al.* A neutralizing antibody selected from plasma cells that binds to group 1 and group 2 influenza A hemagglutinins. *Science* **333**, 850-856 (2011).
6. Nakamura, G., *et al.* An in vivo human-plasmablast enrichment technique allows rapid identification of therapeutic influenza A antibodies. *Cell Host Microbe* **14**, 93-103 (2013).
7. Joyce, M.G., *et al.* Vaccine-Induced Antibodies that Neutralize Group 1 and Group 2 Influenza A Viruses. *Cell* **166**, 609-623 (2016).
8. Andrews, S.F., *et al.* Preferential induction of cross-group influenza A hemagglutinin

stem-specific memory B cells after H7N9 immunization in humans. *Sci Immunol* **2**(2017).

9. McCarthy, K.R.*, et al.* Memory B Cells that Cross-React with Group 1 and Group 2 Influenza A Viruses Are Abundant in Adult Human Repertoires. *Immunity* **48**, 174-184 e179 (2018).

10. Andrews, S.F.*, et al.* Immune history profoundly affects broadly protective B cell responses to influenza. *Sci Transl Med* **7**, 316ra192 (2015).

11. DeKosky, B.J.*, et al.* In-depth determination and analysis of the human paired heavy- and light-chain antibody repertoire. *Nat Med* **21**, 86-91 (2015).

REVIEWER COMMENTS, second round

Reviewer #2 (Remarks to the Author):

The revisions in the current version of the MS largely improved the quality and scope of the study.

I believe that the data presented in Ref Figure 2. "Differential binding activity of HA stem mAbs to the cleaved HAs between H1 and H3 subtypes." present important observations about the reactivity of the presented mAbs to this particular epitope. I think these data should be included in the MS (at least as Supplementary Data) and a paragraph on the implications of these observations (i.e. differential binding to H1 vs H3 cleaved HAs) should be included in the Discussion section.

Reviewer #3 (Remarks to the Author):

The manuscript " Unique binding pattern for a lineage of human antibodies with broad reactivity against influenza A virus" by Xiaoyu Sun et al. shows a novel class of neutralizing antibodies having relatively broad binding breadth to neutralize both H3N2 and H1N1viruses. The authors proposed that 28-12 antibodies acquired these binding abilities in two steps. In this antibody, the germline sequence of VH3-48/VK1-12 would be initially selected by the contiguous epitope of H3N2 HA and evolved via somatic hypermutation enable to cross-react with a conformational epitope of H1N1 HA. After being amended, I can see that the revised version has dealt carefully with the points raised by three referees by dedicated efforts. This manuscript has been improved clearly, and all additional data and text support the points of argumentation. Therefore, this work warrants publication in Nature communication.

REVIEWERS' COMMENTS

Reviewer #2:

The revisions in the current version of the MS largely improved the quality and scope of the study.

I believe that the data presented in Ref Figure 2. "Differential binding activity of HA stem mAbs to the cleaved HAs between H1 and H3 subtypes." present important observations about the reactivity of the presented mAbs to this particular epitope. I think these data should be included in the MS (at least as Supplementary Data) and a paragraph on the implications of these observations (i.e. differential binding to H1 vs H3 cleaved HAs) should be included in the Discussion section.
Answer:
Many thanks for the reviewer's constructive comments and suggestions. We have presented the data (differential binding to H1 vs H3 cleaved HAs of antibody 28-12) in the Supplementary Figure 8 and included several sentences on the data description and discussion in the Results and Discussion sections. **(Article, lines 252-256 and lines 331-336)**

Reviewer #3 (Remarks to the Author):

The manuscript " Unique binding pattern for a lineage of human antibodies with broad reactivity against influenza A virus" by Xiaoyu Sun et al. shows a novel class of neutralizing antibodies having relatively broad binding breadth to neutralize both H3N2 and H1N1viruses. The authors proposed that 28-12 antibodies acquired these binding abilities in two steps. In this antibody, the germline sequence of VH3-48/VK1-12 would be initially selected by the contiguous epitope of H3N2 HA and evolved via somatic hypermutation enable to cross-react with a conformational epitope of H1N1 HA.
After being amended, I can see that the revised version has dealt carefully with the points raised by three referees by dedicated efforts. This manuscript has been improved clearly, and all additional data and text support the points of argumentation. Therefore, this work warrants publication in Nature communication.
Answer:
Many thanks for the reviewer's constructive comments and suggestions previously, which have helped us a lot to improve this manuscript.
